# Supplementary material for: Organisational and individual readiness for change to respectful maternity care practice and associated factors in Ibadan, Nigeria: a cross-sectional survey
Source: BMJ Open. 2022 Nov 22;12(11):e065517. doi: 10.1136/bmjopen-2022-065517 (PMC9685001; doi:10.1136/bmjopen-2022-065517)
Supplement: Supplementary data [file bmjopen-2022-065517supp001.pdf]

Additional file 1

What is Respectful Maternity Care?

Respectful Maternity Care (RMC) is a human rights approach to childbirth care practice. It is a new strategy for caring for women in labour which we are yet to commence implementing in this health facility. We are interested in knowing how health facilities offering childbirth
services, their managers and individual workers are READY to integrate respectful maternity care into their routine childbirth services.

What is Respectful Maternity care?
Simply, it means the following

1. The preferences of the client must be respected, and she must be involved in the decision making regarding her health.
2. She must be allowed a companion during birth as recommended by the WHO
3. She must be free to move about during labour if she so wishes even in the second stage before the urge to deliver and not restricted to one position.
4. If classified as a low risk pregnant woman, she should be allowed oral fluids or food while in labour as evidence has shown no negative outcomes following this.
5. Her privacy must be ensured by providing one private cubicle or space per woman in labour and information about her should not be shared openly.
6. If she prefers to deliver her child squatting, the health care provider must be willing to support her in the decision.
7. Equitable services must be delivered to her regardless of her personal characteristics.
8. When she calls for help during labour, she must not be denied nor neglected.
9. If she is unable to pay her bills, a consensus must be reached with her on how to pay rather than detaining her illegally for the inability to pay.
10. Overall, she must receive the utmost respectful and dignified care, that she deserves as her fundamental human rights.
