# Supplementary material for: Organisational and individual readiness for change to respectful maternity care practice and associated factors in Ibadan, Nigeria: a cross-sectional survey
Source: BMJ Open. 2022 Nov 22;12(11):e065517. doi: 10.1136/bmjopen-2022-065517 (PMC9685001; doi:10.1136/bmjopen-2022-065517)
Supplement: Supplementary data [file bmjopen-2022-065517supp002.pdf]

Confidential

Rmc Readiness Assessment Of Health Care Providers

Record ID ________________

Longitude ________________

Latitude ________________

Time of start of interview ________________

1. RMC Record ID (For example Adeoyo/HW/001) ________________

2. Which Local Government Area (LGA) is this facility situated
   - ◯ Ibadan North
   - ◯ Ibadan North East
   - ◯ Ibadan North West
   - ◯ Ibadan South East
   - ◯ Ibadan South West

3. Level of Public Health Facility ◯ Primary ◯ Secondary

4. Date of Birth (Example Day-Month-Year ________________

Today's date ________________

5. Age ________________

6. Highest Level of education completed
   - ◯ Secondary education
   - ◯ School of Health Technology
   - ◯ Nursing School
   - ◯ Nurse/Midwifery School
   - ◯ Medical School
   - ◯ Master's program / Part 1 Fellowship Exams
   - ◯ Ph.D. program/ Part 2 Fellowship Exams

7. Professional type
   - ◯ Specialist Consultant
   - ◯ Resident doctor
   - ◯ Medical Officer
   - ◯ House Officer
   - ◯ Nurse
   - ◯ Nurse/Midwife
   - ◯ CHO
   - ◯ CHEW
   - ◯ Health Assistant/ Nurse Auxilliary/ Nurse Aide

In which sub-unit are you working in this health facility or do you work across all the units?
- ◯ I work in the antenatal unit only
- ◯ I work in the labour room only
- ◯ I work in the post-natal unit only
- ◯ I work in all the units

8. Your total income in a month (from all sources) may fall within which of these ranges	◯ < ₦30,000 (83.3USD)<br>◯ ₦30, 000 - ₦50,000 (138.9 USD)<br>◯ ₦51,000 - ₦70,000 (194.4 USD)<br>◯ ₦71, 000 - ₦90,000 (250 USD)<br>◯ ₦91,000 - ₦110,000 (305.5 USD)<br>◯ ₦110,000- ₦200,000 (555.5 USD)<br>◯ ₦200,000 - ₦400,000 (1,111 USD)<br>◯ More than ₦400,000 (1,111 USD)
9. Specifically, these total Income from (all sources) per month will be about ___ in Naira (write only in figures)	________________
10. The year you completed pre-service training (graduated from school) for your current profession	________________
11. Years of professional experience after pre-service training	________________
12. The year you started working in this facility as one of the following (nurse/midwife//CHO/CHEW)	________________
13. Years of experience working in this facility as one of the following (nurse/midwife/doctor/CHO/CHEW)	________________
14. Have you ever been promoted in your current job?	◯ Yes ◯ No
15. In which year were you last promoted in your current job (OR Moved to the next level or higher level for doctors?	________________
16. No of years ago you were last promoted	________________
17. The current level of the respondent in the Oyo State civil service employment (based on ranks like level 12. Write only the figure, eg. 12)	________________
18. Sex of the health care provider	◯ Male ◯ Female

Which of these do you think women should have as a Fundamental Human Right to during childbirth and in what frequency? If they should not have a right to it, please, select "Never". If they should, then select best of the other options. (A right is something they can demand for if not given)

	Always (Nigbogbo igba)	Very frequently (Ni lemolemo)	Occasionally (Ni eknankan)	Rarely (Ko wopo rara)	Never (Ko sele ri)

Confidential

1. Right to know / ask about the professional identity and qualifications of those involved with her care	○	○	○	○	○
2. Right to communicate with caregivers and receive all care in privacy	○	○	○	○	○
3. Right to full and clear information about what is being done for her and their benefits, risks and costs (on the procedures, drugs, tests and treatments offered to her)	○	○	○	○	○
4. Right to accept or refuse procedures, drugs, tests and treatments, and to have her choices honored	○	○	○	○	○
5. Right to be informed if her caregivers wish to enroll her or her infant in a research study	○	○	○	○	○
6. Right to unrestricted access to her file and all available records about her pregnancy, labor, birth, postpartum	○	○	○	○	○
7. Right to receive maternity care that is appropriate to her cultural and religious background.	○	○	○	○	○
8. Right to receive information in a language in which she can communicate (like getting her an interpreter) where necessary	○	○	○	○	○
9. Right to have a family member of her choice present during all the aspects/ stages of her labour and childbirth (having a birth companion)	○	○	○	○	○
10. Right to freedom of movement during labor, not hindered by tubes, wires or other apparatus	○	○	○	○	○

11. Right to virtually uninterrupted contact with her newborn from the moment of birth, as long as she and her baby are healthy and do not need care that requires separation	○	○	○	○	○
12. Right to receive a pain relief during labour with information on the type of pain relief, and the risks and benefits	○	○	○	○	○
13. Right to deciding the position of birth (Squatting)	○	○	○	○	○

Has any of the following ever been done to women who have come to deliver in this hospital in the past 1 year to the best of your knowledge (not necessarily by you)? Kindly select the appropriate response

	Always	Very frequently	Occasionally	Rarely	Never
1. Physical abuse (slapping or hitting pinching, beating or attempts made to)	○	○	○	○	○
2. Verbal abuse (saying sentences to pregnant women in labour that may sound abusive like "was I there when you were getting impregnated""?)	○	○	○	○	○
3. Not providing information on the care to be provided or the procedure to be done before doing it	○	○	○	○	○
4. Not obtaining informed consent or a go ahead before procedures or examinations are done (even if information was provided about it)	○	○	○	○	○
5. Lack of privacy (not screening during examinations)	○	○	○	○	○
6. Lack of confidentiality (discussing patients' details openly or patients' files kept indiscriminately)	○	○	○	○	○
7. Discrimination based on age, economic / financial status, ethnicity, religion	○	○	○	○	○

8. A birth companion of her choice NOT allowed to be present with the woman all through the stages during labour	○	○	○	○	○
9. Women not allowed to move about during labour but must lie down in one position	○	○	○	○	○
10. Women not given an option to choose a position of birth (like squatting to deliver) or denial of their choice	○	○	○	○	○
11. Unnecessary separation of mother and newborn after the birth	○	○	○	○	○
12. Leaving the woman alone or unattended or delaying attending to the woman after being called / sent for	○	○	○	○	○

13. Other types of similar actions done to women during childbirth in this health facility, please, specify ________________________________

How long are women kept on admission at the facility after an uncomplicated delivery on the average before discharge home?

○ Less than 6 hours
○ 6-12 hours
○ More than 12 hours to a maximum of 24 hours
○ More than 24 hours
○ Don't know

Have you ever heard of Respectful Maternity Care (RMC) before? ○ Yes ○ No

Readiness Assessment for a Change to a Respectful Maternity Care Practice

Respectful Maternity Care (RMC) is a human rights approach to childbirth care practice. It is a new strategy for caring for women in labour which we are yet to commence implementing in this health facility. We are interested in knowing how health facilities offering childbirth services,
their managers and individual workers are READY to integrate respectful maternity care into their routine childbirth services.
What is Respectful Maternity care?
Simply, it means the following
1. The preferences of the client must be respected, and she must be involved in the decision making regarding her health.
2. She must be allowed a companion during birth as recommended by the WHO
3. She must be free to move about during labour if she so wishes even in the second stage before the urge to deliver and not restricted to one position.
4. If classified as a low risk pregnant woman, she should be allowed oral fluids or food while in labour as evidence has shown no negative outcomes following this.
5. Her privacy must be ensured by providing one private cubicle or space per woman in labour and information about her should not be shared openly.

6. If she prefers to deliver her child squatting, the health care provider must be willing to support her in the decision.
7. Equitable services must be delivered to her regardless of her personal characteristics.
8. When she calls for help during labour, she must not be denied nor neglected.
9. If she is unable to pay her bills, a consensus must be reached with her on how to pay rather than detaining her illegally for the inability to pay.
10. Overall, she must receive the utmost respectful and dignified care, that she deserves as her fundamental human rights.

Respectful Maternity Care can be implemented in this health facility

◯ Strongly disagree
◯ Somewhat disagree
◯ Neither agree nor disagree
◯ Somewhat agree
◯ Strongly agree

Organizational Readiness for a change to RMC Practice

Please select one option for each of these sentences below that best describes how much you think your health facility management and staff are READY to integrate respectful maternity care (RMC) practice into the routine childbirth/ delivery care services in this facility (Please, tick one of these 5 options: Strongly Disagree=D, Somewhat Disagree= SWD, Neither Agree nor Disagree =NA/D, Somewhat Agree= SWA, Strongly Agree= A)

	Strongly Disagree	Somewhat disagree	Neither agree nor disagree	Somewhat agree	Strongly Agree
6. Health workers in this health facility want to (will not mind to) implement this change to RMC practice.	◯	◯	◯	◯	◯
2. Health workers in this health facility are committed to implementing this change to RMC practice	◯	◯	◯	◯	◯
9. Health workers in this health facility are determined to implement this change to RMC practice.	◯	◯	◯	◯	◯
4. Health workers in this health facility will do whatever it takes to implement this change to RMC practice.	◯	◯	◯	◯	◯
11. Health workers in this health facility are self-motivated to implement this change to RMC practice.	◯	◯	◯	◯	◯

Confidential

1. Health workers in this health facility feel confident that the facility management can get people invested in implementing this change to RMC practice.	○	○	○	○	○
5. Health workers in this health facility feel confident that the facility managers can support people to adjust to this change to RMC practice.	○	○	○	○	○
7. Health workers in this health facility feel confident that they can keep pushing to implementing this change to RMC practice.	○	○	○	○	○
3. Health workers in this health facility feel confident that they can keep track of progress in implementing this change to RMC practice.	○	○	○	○	○
10. Health workers in this health facility feel confident that they can coordinate tasks so that implementation goes smoothly to RMC practice.	○	○	○	○	○
8. Health workers in this health facility feel confident that they can handle the challenges that might arise in implementing this change to RMC practice.	○	○	○	○	○
12. Health workers in this health facility feel confident that they can manage the politics of implementing this change to RMC practice.	○	○	○	○	○

The score for commitment to change ________________________________

The MEAN score for Change commitment ________________________________

The score for Change Efficacy ________________________________

The MEAN Score for Change Efficacy ________________________________

Confidential

Informational assessment- Regarding a change to a respectful maternity care practice being integrated into your routine childbirth care, please, select the most appropriate option to the sentences below

	Strongly disagree	Somewhat disagree	Neither agree nor disagree	Somewhat agree	Strongly agree
1. Health workers in this health facility believe we have the equipment we need to implement this change to a RMC practice	○	○	○	○	○
2. Health workers in this health facility believe we have the expertise we need to implement this change to RMC practice	○	○	○	○	○
3. Health workers in this health facility believe we have the time we need to implement this change to a RMC practice	○	○	○	○	○
4. Health workers in this health facility believe we have the skills we need to implement this change to a RMC practice	○	○	○	○	○
5. Health workers in this health facility believe we have the resources we need to implement this change to a RMC practice	○	○	○	○	○
6. Health workers in this health facility know how much time it will take to implement this change	○	○	○	○	○
7. Health workers in this health facility know what resources we will need to implement this change	○	○	○	○	○
8. Health workers in this health facility know what each of us has to do to implement this change Given	○	○	○	○	○

Change Valence Assessment-
Please select the most appropriate option for each of these statements below regarding a change to integrate respectful maternity care (RMC) practice into the routine childbirth/ delivery care services in this facility.

	Strongly disagree	Somewhat disagree	Neither agree nor disagree	Somewhat agree	Strongly agree

1. Health workers in this health facility feel that a change to a respectful maternity care is compatible with our values	◯	◯	◯	◯	◯
2. Health workers in this health facility feel that we need to implement this change to RMC practice	◯	◯	◯	◯	◯
3. Health workers in this health facility believe this change to RMC practice will benefit our community	◯	◯	◯	◯	◯
4. Health workers in this health facility believe this change to RMC practice will make things better	◯	◯	◯	◯	◯
5. Health workers in this health facility believe this change to RMC practice is a good idea	◯	◯	◯	◯	◯
6. Health workers in this health facility value this change to a RMC practice	◯	◯	◯	◯	◯

Individual Readiness for Change Assessment Tool

The options below are assessing your own personal readiness for your health facility to integrate respectful maternity care practices into your routine childbirth care. Select one option each

	Strongly disagree	Disagree	Slightly disagree	Do not disagree nor agree	Slightly Agree	Agree	Strongly Agree
1. When changes such as a change to RMC practice is about to occur in our health facility, I believe that I am ready to cope with them.	◯	◯	◯	◯	◯	◯	◯
2. I usually try to convince people in my health facility to accept the change such as a change to RMC practice	◯	◯	◯	◯	◯	◯	◯
3. When changes occur in my health facility, I tend to complain about them rather than deal with them*	◯	◯	◯	◯	◯	◯	◯

4. I believe that I am more ready to accept a change to RMC practice than my colleagues.	○	○	○	○	○	○	○
5. I don't worry about changes in my health facility because I believe that there is always a way to cope with them	○	○	○	○	○	○	○
6. When changes such as a change to RMC practice occur in my company, I always have the intention to support them.	○	○	○	○	○	○	○

Concerning the availability of the resources needed to integrate a respectful maternity care practice into the routine childbirth care in this facility, please select the best option

	Disagree	Somewhat disagree	Neither agree nor disagree	Somewhat agree	Agree
1. We have adequate number of trained and competent skilled birth attendants (e.g. nurse, doctors and nurse auxiliaries like CHO/CHEWS) that can provide RMC care to all women	○	○	○	○	○
2. The Management of our health facility are very familiar and well sensitized to the provision of a respectful maternity care practice	○	○	○	○	○
3. Regular practical, in-service training on the provision of respectful maternity care is being done for the staff in this health facility	○	○	○	○	○
4. There are written, up-to-date guidelines on the provision of a respectful maternity care during childbirth in this health facility	○	○	○	○	○
5. There are established and standard informed consent system in place and forms to be used for procedures requiring such (for example episiotomy	○	○	○	○	○

6. There are health education materials on respectful maternity care in pictures and the languages of the communities served in this health facility	○	○	○	○	○
7. Rooming-in (Nursing baby by the mother's side) is practiced in this facility to allow women and their babies to remain together	○	○	○	○	○
8. There are clean, appropriately illuminated, well ventilated labour, and childbirth areas	○	○	○	○	○
9. There are clean and accessible toilets and bathrooms for use by women in labour/ post delivery	○	○	○	○	○
10. There is safe drinking water, and a hand washing station, with soap and water (preferably running water) or alcohol-based hand rubs	○	○	○	○	○
11. Curtains, screens, partitions are available and are being used to maintain privacy for women during ;labour and childbirth	○	○	○	○	○
12. There are sufficient bed capacity (needed number of beds for facility)	○	○	○	○	○
13. Facilities for companions of women in labour, including a private space (or partitioned with curtain) that can allow for the woman and her companion are available	○	○	○	○	○
14. Basic and adequate equipment for labor and childbirth that is available in sufficient quantities at all times in the labor and childbirth areas	○	○	○	○	○
15. Continuous energy/ power supply in the labor, childbirth and neonatal areas	○	○	○	○	○

Confidential

16. Staff meetings are held regularly to review our childbirth practices if respectful (RMC practices) or not in this health facility	○	○	○	○	○
17. There is a suggestion box for service users (clients/ patients) and providers to submit complaints to the management	○	○	○	○	○
18. Establishment of accountability mechanisms for redress in the event of mistreatment or violations (eg. disciplinary committee to handle report of mistreatment of women or providers)	○	○	○	○	○

These last set of questions help us to self-evaluate ourselves as a person and may elicit the factors that are associated with our readiness for a change to a respectful maternity care

Health care providers' Core Self-Evaluation Personality Traits- Kindly select the most appropriate option to each of the sentences below as it describes you as a person

	Strongly disagree	Somewhat disagree	Neither agree nor disagree	Somewhat agree	Strongly agree
1. I am confident I'll get the success I deserve in life.	○	○	○	○	○
2. Sometimes I feel miserable*	○	○	○	○	○
3. When I try, I generally succeed	○	○	○	○	○
4. Sometimes when I fail I feel worthless*	○	○	○	○	○
5. I complete tasks successfully	○	○	○	○	○
6. Sometimes, I do not feel I am in control of my work*	○	○	○	○	○
7. Overall, I am satisfied with myself	○	○	○	○	○
8. I am filled with doubts about my competence*	○	○	○	○	○
9. I determine / choose what will happen in my life	○	○	○	○	○
10. do not feel in control of my success in my career*	○	○	○	○	○

11. I am capable of coping with most of my problems	◯	◯	◯	◯	◯
12. There are times when things look pretty bleak and hopeless to me*	◯	◯	◯	◯	◯

Health care providers' Job satisfaction- Regarding the extent to which you are satisfied with your job, kindly select the most appropriate option for the sentences below

	Strongly disagree	Somewhat disagree	Neither agree nor disagree	Somewhat Agree	Strongly agree
1. My salary is fair compared to other staff in other southwest states with the same level of responsibility	◯	◯	◯	◯	◯
2. My benefits are fair compared to other staff at my level	◯	◯	◯	◯	◯
3. My job description is clear to me, accurate and up to date	◯	◯	◯	◯	◯
4. My supervisor and I have agreed on the priorities of my job	◯	◯	◯	◯	◯
5. I get clear feedback from my supervisors about how well I am performing on my job	◯	◯	◯	◯	◯
6. My annual performance appraisal is based on the priorities in my workplan (my actual performance)	◯	◯	◯	◯	◯
7. My supervisor seeks my input when faced with a challenge or problem	◯	◯	◯	◯	◯
8. The organization (the management of this facility) acknowledges and values my work	◯	◯	◯	◯	◯
9. The organization (the State Ministry of Health) provide me with the essential coaching and training to do my job.	◯	◯	◯	◯	◯
10. The organization works (as much as possible) to provide me with opportunities for career growth.	◯	◯	◯	◯	◯

Any other important comments or observations ________________________________

Time to END of interview ________________________

Confidential

Name of Interviewer	◯ Bukola<br>◯ Dr. Esan<br>◯ Any other

Esan OT, *et al. BMJ Open* 2022; 12:e065517. doi: 10.1136/bmjopen-2022-065517
