# Supplementary figures and images for: Organisational and individual readiness for change to respectful maternity care practice and associated factors in Ibadan, Nigeria: a cross-sectional survey
Source: BMJ Open. 2022 Nov 22;12(11):e065517. doi: 10.1136/bmjopen-2022-065517 (PMC9685001; doi:10.1136/bmjopen-2022-065517)

Additional file 3

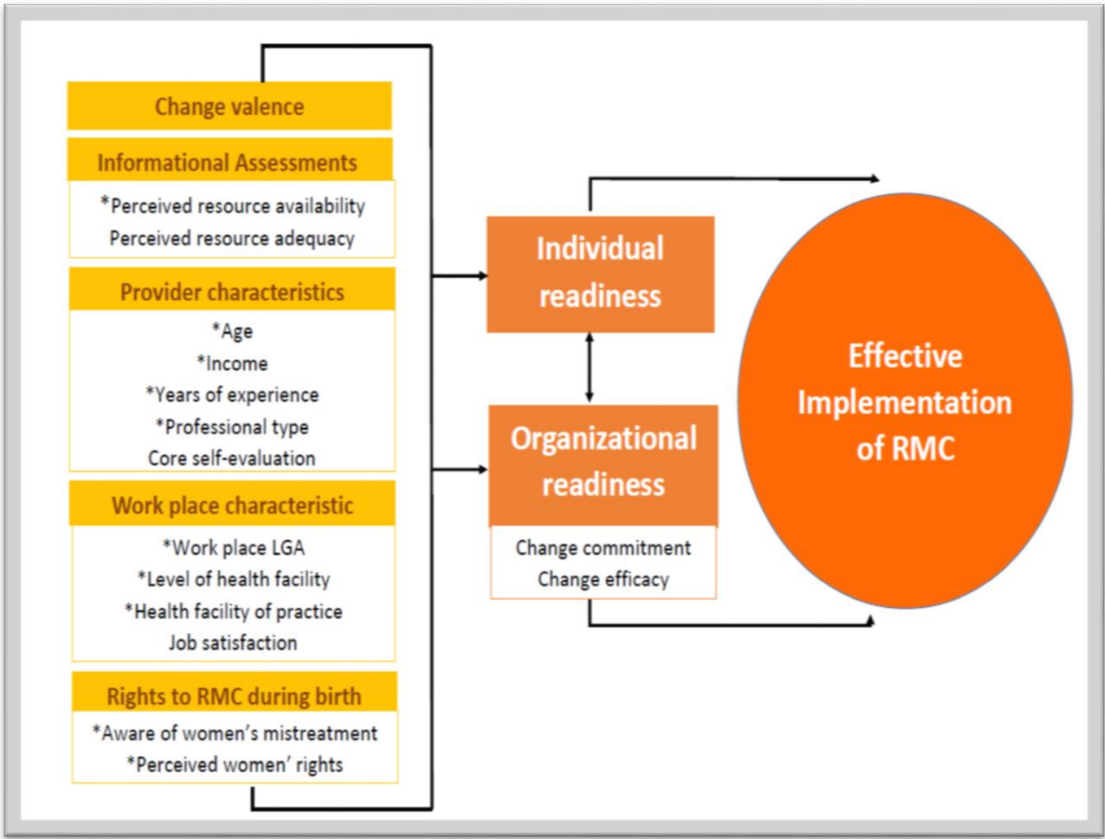

Study Analytical Frameworks (Note: \*proposed as predictor variables)

Supplement: Supplementary data [file bmjopen-2022-065517supp003.pdf]
