# Supplementary material for: Organisational and individual readiness for change to respectful maternity care practice and associated factors in Ibadan, Nigeria: a cross-sectional survey
Source: BMJ Open. 2022 Nov 22;12(11):e065517. doi: 10.1136/bmjopen-2022-065517 (PMC9685001; doi:10.1136/bmjopen-2022-065517)
Supplement: Supplementary data [file bmjopen-2022-065517supp004.pdf]

Additional file 4: Breakdown of tools in the health provider survey instrument

	Name of Tool and Sections	Source	Items	Response type	Alpha coeff.
1	*Organisational Readiness for Implementing change (ORIC)	Shea et al [19]	12	Likert scale 1-5	0.949
2	*Individual readiness for change	Vakola et al[8]	6	Likert scale 1-7	0.733
3	Socio-demographic characteristics	Adapted from the literature	15		
4	Perception on women's rights during childbirth	Childbirth Connection[20]	13	Likert scale 1-5	0.575
5	Provider awareness of mistreatment in their own facility	Maternal & Child Health program[21]	12	Likert scale 1-5	0.638
6	Change valence	Shea et al[19]	6	Likert scale 1-5	0.902
7	Informational assessments	Phillip[22]	8	Likert scale 1-5	0.648
8	Perception on RMC resource availability	WHO Recommendation for labour[1]	18	Likert scale 1-5	0.669
10	Core self-evaluation tool	Judge et al[18]	12	Likert scale 1-5	0.598
11	Employee job satisfaction tool	Management Sciences for Health[23]	10	Likert scale 1-5	0.603
	Total		112		

*Outcome variables; WHO: World Health Organisation
