# Supplementary material for: Organisational and individual readiness for change to respectful maternity care practice and associated factors in Ibadan, Nigeria: a cross-sectional survey
Source: BMJ Open. 2022 Nov 22;12(11):e065517. doi: 10.1136/bmjopen-2022-065517 (PMC9685001; doi:10.1136/bmjopen-2022-065517)
Supplement: Supplementary data [file bmjopen-2022-065517supp005.pdf]

Additional file 5: Summarised PCA Findings

1. Principal Component Analsysis Results for the scale assessing Health Provider's perception of women's rights during childbirth.

Test	Measure	Statistics
Kaiser-Meyer-Olkin (KMO)	Sampling adequacy	[a]0.620
Bartlett's	Sphericity	$\chi^2 = 229.126$
		df = 78
		p < 0.001*

Total Variance explained

Component	Eigenvalues	% of variance	Cumulative %
1	2.326	17.89	17.89
2	1.448	11.14	29.04

* Statistically significant   [a] Sample is adequate   (No of observations -203)

Principal component eignevectors for 4 of 13 components

Variable	Comp1	Comp2	Comp3	Comp4	Comp5
right_Know~r	0.3444	-0.0563	0.0809	0.4687	0.1214
right_priv~y	0.2609	-0.5047	0.0694	0.1062	0.1655
right_info~n	0.1528	-0.1522	0.1208	0.0868	0.6739
right_refu~t	0.1797	0.3353	-0.2941	0.3634	0.0657
right_rese~t	0.2260	0.4095	-0.3549	0.1507	0.2613
right_acce~s	0.2397	-0.2237	-0.3648	-0.3109	0.1185
right_cult~e	0.3100	-0.3255	0.0846	0.2425	-0.4273
right_nola~r	0.0559	0.2120	0.7022	-0.0018	0.2357
right_comp~p	0.3543	0.0971	0.3081	-0.0594	-0.2283
right_mobi~r	0.3228	-0.0195	-0.0627	-0.3274	0.1690
right_noba~n	0.1915	0.4536	0.1623	-0.2516	-0.0634
right_pain~f	0.4288	0.1441	-0.0478	0.0854	-0.3068
right_birt~n	0.3157	-0.0396	-0.0281	-0.5196	0.0064

Correlation matrix showing the total, mean scores and pca predicted scores (final rights) for health provider's perception and frequency of women's rights during childbirth.

	Total scores _perceived women's rights	Mean scores _perceived women's rights	No of items agreed to_ _perceived women's rights	Pca scores _perceived women's rights
Total scores _	1.000			
Mean scores	1.000	1.000		
No of items agreed	0.9157	0.9157	1.000	
Pca scores	0.9919	0.9919	0.9073	1.000

2. Principal Component Analsysis Results for the scale assessing Health Provider's awareness of the frequency of mistreatment f o women during childbith at their own health facilities

Test	Measure	Statistics
Kaiser-Meyer-Olkin (KMO)	Sampling adequacy	[a]0.720
Bartlett's	Sphericity	$\chi^2 = 357.784$
		df = 66
		p < 0.001*

Total Variance explained

Component	Eigenvalues	% of variance	Cumulative %
1	2.783	23.19	23.19
2	1.440	12.00	35.19

* Statistically significant [a] Sample is adequate (No of observations -211)

Principal component eignevectors for 4 of 12 components

Variable	Comp1	Comp2	Comp3	Comp4
physical_a~e	0.1505	-0.1880	0.5230	0.3352
verbal_abuse	0.3260	-0.3530	0.3263	0.1119
lack_of_in~n	0.4237	-0.0759	-0.2696	0.2439
no_informe~t	0.2234	0.1038	-0.4436	0.4205
lack_of_pr~y	0.3758	0.2156	0.0596	-0.2068
lack_confi~y	0.4621	0.0288	-0.1338	-0.1383
discrimina~n	0.4057	-0.1312	-0.1291	-0.0910
no_birth_c~n	0.1262	0.3171	0.2598	0.2618
no_movt_in~r	0.0401	0.4763	0.3472	-0.1348
no_choice_~n	0.1061	0.5535	0.1156	0.3253
separation~y	0.1021	-0.3402	0.3182	0.0749
abandonmen~e	0.2883	0.0975	0.0988	-0.6087

Correlation matrix showing the total scores, mean scores and pca predicted scores (final mistreatment) for health provider's awareness of the frequency of women's mistreatment during childbirth at their facilities

	Total scores _perceived women's rights	Mean scores _perceived women's rights	No of items agreed to_ _perceived women's rights	Pca scores _perceived women's rights
Total scores _	1.000			
Mean scores	1.000	1.000		
No of items agreed	-0.7437	-0.7437	1.000	
Pca scores	0.8658	0.8658	-0.4638	1.000

3. Principal component analysis results for the scale assessing health provider's perception of resource availability for the implementation of RMC as recommended by the World Health Organisation

Test	Measure	Statistics
Kaiser-Meyer-Olkin (KMO)	Sampling adequacy	[a]0.640
Bartlett's	Sphericity	$\chi^2 = 557.535$
		df = 153
		p < 0.001*

Total Variance explained			
**Component**	**Eigenvalues**	**% of variance**	**Cumulative %**
1	2.968	16.49	16.49
2	2.242	12.46	28.95

* Statistically significant [a] Sample is adequate (No of observations -173)

Principal component eignevectors for 6 of 18 components

Variable	Comp1	Comp2	Comp3	Comp4	Comp5	Comp6
adequate_s~f	0.1937	-0.3240	0.2060	-0.0295	0.1330	-0.1381
mgt_sensit~d	0.1834	0.1923	-0.4623	-0.0580	0.1970	-0.0811
regular_rm~g	0.3397	-0.0619	-0.3633	-0.2583	-0.1123	-0.0819
written_gu~s	0.3728	-0.0247	0.0683	-0.3796	-0.1623	0.0967
informed_c~t	0.0625	0.5407	0.1832	-0.1032	0.0221	0.0626
rmc_educat~s	0.2679	0.0166	0.3492	-0.3203	-0.1146	0.1170
rooming_in	-0.0196	0.2111	-0.0005	0.0803	-0.0010	0.6030
clean_priv~e	0.2048	0.2019	-0.1084	0.3604	-0.2740	0.0965
clean_bath~s	0.2307	0.1783	0.0003	0.3818	-0.3967	0.0724
safe_water~e	0.0780	0.1868	0.1777	0.2380	0.0806	-0.6417
curtains_a~s	0.3270	0.1311	0.1233	0.1952	-0.1454	-0.2312
adequate_b~s	0.2094	-0.1672	0.2842	0.2932	0.4128	0.1370
space_woma~s	0.1107	-0.3020	0.3363	-0.0534	-0.3750	-0.0117
adequate_l~t	0.1908	-0.3234	-0.0153	0.3417	0.2592	0.2372
power_supp~r	0.3325	-0.0968	-0.0659	0.0334	-0.0543	0.1038
rmc_practi~w	0.2486	-0.2531	-0.4220	0.0271	0.0132	-0.0762
suggestion~x	0.2576	0.2220	0.1264	-0.2739	0.4053	-0.0240
redress_co~e	0.2430	0.2129	0.0257	0.1119	0.2920	0.0707

Correlation matrix showing the total scores, mean scores and principal component analysis predicted scores (final res) predicted scores for health provider's perception on the availability of resources needed to implement respectful maternity care as recommended by the World Health Organisation

	Total scores _resource availability	Mean scores _ resource availability	No of items agreed to_ _ resource availability	Pca scores _ resource availability
Total scores _	1.000			
Mean scores	1.000	1.000		
No of items agreed	0.9206	0.9206	1.000	
Pca scores	0.9576	0.9576	0.8657	1.000
