# Supplementary material for: Organisational and individual readiness for change to respectful maternity care practice and associated factors in Ibadan, Nigeria: a cross-sectional survey
Source: BMJ Open. 2022 Nov 22;12(11):e065517. doi: 10.1136/bmjopen-2022-065517 (PMC9685001; doi:10.1136/bmjopen-2022-065517)
Supplement: Author's manuscript [file bmjopen-2022-065517.draft_revisions.pdf]

BMJ Open is committed to open peer review. As part of this commitment we make the peer review history of every article we publish publicly available.

When an article is published we post the peer reviewers' comments and the authors' responses online. We also post the versions of the paper that were used during peer review. These are the versions that the peer review comments apply to.

The versions of the paper that follow are the versions that were submitted during the peer review process. They are not the versions of record or the final published versions. They should not be cited or distributed as the published version of this manuscript.

BMJ Open is an open access journal and the full, final, typeset and author-corrected version of record of the manuscript is available on our site with no access controls, subscription charges or pay-per-view fees (http://bmjopen.bmj.com).

If you have any questions on BMJ Open's open peer review process please email info.bmjopen@bmj.com

BMJ Open

Organisational and individual readiness for change to respectful maternity care practice and associated factors in Ibadan, Nigeria: a cross-sectional study

Journal:	*BMJ Open*
Manuscript ID	bmjopen-2022-065517
Article Type:	Original research
Date Submitted by the Author:	09-Jun-2022

Complete List of Authors:	Esan, Oluwaseun; Obafemi Awolowo University College of Health Sciences, Department of Community Health, Faculty of Clinical Sciences; University of the Witwatersrand Faculty of Health Sciences, Centre for Health Policy, School of Public Health<br>Maswime, Salome; University of Cape Town Faculty of Health Sciences, Global Surgery Division, Department of Surgery<br>Blaauw, Duane; University of the Witwatersrand Faculty of Health Sciences, Centre for Health Policy, School of Public Health

Keywords:	Change management < HEALTH SERVICES ADMINISTRATION & MANAGEMENT, Health policy < HEALTH SERVICES ADMINISTRATION & MANAGEMENT, PUBLIC HEALTH, REPRODUCTIVE MEDICINE, Quality in health care < HEALTH SERVICES ADMINISTRATION & MANAGEMENT, Organisation of health services < HEALTH SERVICES ADMINISTRATION & MANAGEMENT

SCHOLARONE™
Manuscripts

I, the Submitting Author has the right to grant and does grant on behalf of all authors of the Work (as defined in the below author licence), an exclusive licence and/or a non-exclusive licence for contributions from authors who are: i) UK Crown employees; ii) where BMJ has agreed a CC-BY licence shall apply, and/or iii) in accordance with the terms applicable for US Federal Government officers or employees acting as part of their official duties; on a worldwide, perpetual, irrevocable, royalty-free basis to BMJ Publishing Group Ltd ("BMJ") its licensees and where the relevant Journal is co-owned by BMJ to the co-owners of the Journal, to publish the Work in this journal and any other BMJ products and to exploit all rights, as set out in our licence.

The Submitting Author accepts and understands that any supply made under these terms is made by BMJ to the Submitting Author unless you are acting as an employee on behalf of your employer or a postgraduate student of an affiliated institution which is paying any applicable article publishing charge ("APC") for Open Access articles. Where the Submitting Author wishes to make the Work available on an Open Access basis (and intends to pay the relevant APC), the terms of reuse of such Open Access shall be governed by a Creative Commons licence – details of these licences and which Creative Commons licence will apply to this Work are set out in our licence referred to above.

Other than as permitted in any relevant BMJ Author's Self Archiving Policies, I confirm this Work has not been accepted for publication elsewhere, is not being considered for publication elsewhere and does not duplicate material already published. I confirm all authors consent to publication of this Work and authorise the granting of this licence.

For peer review only

Organisational and individual readiness for change to respectful maternity care practice and associated factors in Ibadan, Nigeria: a cross-sectional study

1. Dr Oluwaseun Taiwo Esan

Department of Community Health, Faculty of Health Sciences,

Obafemi Awolowo University/ Teaching Hospitals Complex, Ile-Ife, Osun State, Nigeria.

& Centre for Health Policy, School of Public Health, University of the Witwatersrand, Johannesburg, South Africa

Email: o.esan@oauife.edu.ng

ORCID: 0000-0002-2908-6034

2. Professor Salome Maswime

Global Surgery Division, Department of Surgery, Faculty of Health Sciences,

University of Cape Town, Cape Town. South Africa

ORCID: 0000-0003-4013-5164

E-mail: salome.maswime@uct.ac.za

3. Dr Duane Blaauw

Centre for Health Policy, School of Public Health,

Faculty of Health Sciences, University of the Witwatersrand, Johannesburg, South Africa.

ORCID: 0000-0002-0605-7134

E-mail: duane.blaauw@wits.ac.za

Corresponding author:

Name: Dr Oluwaseun Taiwo Esan

E-mail: o.esan@oauife.edu.ng

Word count: 3,981

Abstract

Objectives: This study assessed health providers' organisational and individual readiness for change to respectful maternity care (RMC) practice and their associated factors in Ibadan Metropolis, Nigeria. The study explored the relevance of readiness for change theories to the RMC literature.

Design: An analytical cross-sectional study with standardised structured instruments adapted from the literature.

Setting: Nine public health facilities (5 primary and 4 secondary) in Ibadan Metropolis, Nigeria

Participants: 212 health providers selected via a two-stage cluster sampling

Primary and secondary outcomes: Organisational readiness for change to RMC (ORC_{RMC}) and individual readiness for change to RMC (IRC_{RMC}) scales were scored out of a maximum of 5. We evaluated previously identified predictors of readiness for change (change valence, informational assessments on resource adequacy, core self-evaluation and job satisfaction) and additionally proposed ones from our study (workplace characteristics, awareness of mistreatment during childbirth, perceptions of women's rights and resource availability to implement RMC) on ORC_{RMC} and IRC_{RMC}. Data were adjusted for clustering and analysed using Stata 15. Multiple linear regression was used to identify factors influencing IRC_{RMC} and ORC_{RMC}.

Results: The providers' mean age was 44.0±9.9 with 15.4±9.9 years of work experience. They scored high on awareness of women's mistreatment (3.9±0.5) and women's perceived rights during childbirth (3.9±0.5). They had high ORC_{RMC} (4.1±0.9) and IRC_{RMC} (4.2±0.6) with both weakly but positively correlated (*rho*=0.407, $p<0.001$). Providers also had high change valence (4.5±0.8) but lower perceptions of resource availability (2.7±0.7) and adequacy for implementation (3.3±0.7). Higher provider change valence and informational assessments

For peer review only - http://bmjopen.bmj.com/site/about/guidelines.xhtml

For peer review only

significantly increased both IRC_{RMC} and ORC_{RMC}. Longer years of work experience (p=0.024), providers' personal income (p=0.021) and the health facility of practice significantly influenced ORC_{RMC}.

Conclusion: The health providers in the study valued a change to RMC and believed that both them and their facilities were ready for the change to RMC practice.

Key words: Organisational readiness, individual readiness, readiness for implementing change, respectful maternity care, pre-implementation research, change commitment and efficacy

For peer review only

Strengths and limitations of this study

- To the best of our knowledge, this is the first study to explore the readiness for change theories (organisational and individual readiness for change) to the recently evolving respectful maternity care literature.
- This is the first study to assess both organisational and individual readiness for change on the same population within the same study and objectively assess the relationship between both.
- This is the first study to apply the adapted instrument on individual readiness for change in a health care setting.
- The study was however limited in its geographical extent as it was conducted in Ibadan Metropolis, one metro in Nigeria, even though Ibadan is a cosmopolitan city being the third largest city in Nigeria and the seventh in Africa.
- The study was limited in its scope as tertiary health facilities were not studied because there was only one tertiary health facility serving populations in the study location. Thus the study findings would not be generalisable to health providers' practicing in tertiary health facilities.

For peer review only - http://bmjopen.bmj.com/site/about/guidelines.xhtml

1.1. Background

Respectful maternity care has been defined as "*care organised for and provided to all women in a manner that maintains their dignity, privacy and confidentiality, ensures freedom from harm and mistreatment, and enables informed choice and continuous support during childbirth*" (page 3).[1] It is a human rights approach to maternity care[2] and is recommended as the standard for all women.[3] Several RMC-promoting interventions have been implemented and have shown promising results.[4] For these results to be enduring and sustainable, the health providers will need to embrace and support the interventions. This can be achieved if they are ready for the change to a RMC practice.

Readiness for change measures the extent to which people or organisations are inclined to adopt a change that alters the "status quo".[5] It addresses the psychological and behavioural forms of readiness for change, that is the state of being willing and able to change.[6,7] Some authors also describe it as having a structural component that addresses the presence or absence of financial, material, and human resources needed for a change, such as to RMC practice.[7] Readiness for change is also a multilevel construct measured at individual and organisational levels. Organisational readiness for change is a multifaceted concept that consists of employees' change commitment (their collective resolve) and change efficacy (their perceived shared ability) to implement the change.[6] Individual readiness for change is employee's confidence to manage the change or willingness to accept new roles and adopt new practices.[8]

Readiness for change is a key determinant of implementation success.[9,10] The readiness for change concept has been applied in both health and non-health organisations, however, there are no previous studies on its application to RMC-promoting interventions. Readiness for change to RMC among community, facility and policy stakeholders was mentioned as being responsible for the positive results of a RMC project in Kenya.[11] However, readiness for change was not measured directly in that study.[11] Many RMC-promoting interventions have been

For peer review only - http://bmjopen.bmj.com/site/about/guidelines.xhtml

conducted without prior assessment of individual or organisational readiness for change.[12,13] However, if readiness is assessed and found wanting, efforts can be directed at improving it. If otherwise, this suggests the providers' willingness to accept the change irrespective of the work task demands brought by it. This study assessed health providers' organisational and individual readiness for change to RMC practice and their associated factors in Ibadan Metropolis, Nigeria.

1.2. Methods

This was a cross-sectional study conducted from December 1, 2019 to May 31, 2020 among public health care providers from the five Local Government Areas (LGA) in Ibadan Metropolis, Oyo state, Nigeria. There are 6 public secondary and 26 public primary health facilities in the five LGAs. Maternity care services, including delivery services, are offered in all facilities, with more specialised care at secondary health facilities. Doctors and nurses attend deliveries at both primary and secondary health facilities, while Community Health Officers (CHOs), Community Health Extension Workers (CHEWs) and Health Auxiliaries (HA) only attend deliveries at primary health facilities in the study state.

A two-stage cluster sampling technique was employed to select the health facilities and health providers in the study LGAs. One primary and one secondary health facility were selected in each LGA using simple random sampling, except in one LGA without a secondary health facility. This gave a total of nine health facilities studied (4 secondary and 5 primary health facilities). There were a total of 244 health providers who could attend deliveries in the study facilities (176 in the 4 secondary facilities and 68 in the 5 primary facilities).

A sample size of 210 health providers was calculated using the one-sample mean test[14] in Stata. The parameters used were a change commitment mean of 3.64±0.61 standard deviation (SD), based on a similar study in Switzerland, as a proxy for organisational readiness.[15] The required precision was ±5% about the reference mean, with 90% power, and a design effect of 2[16] for

For peer review only - http://bmjopen.bmj.com/site/about/guidelines.xhtml

the cluster sampling. The number of health providers interviewed at each facility was allocated proportionately to the total number of health providers per professional type at each health facility within the LGA. All the available and consenting health providers at each health facility were interviewed until the required numbers were reached. Ethical approvals were obtained from the Human Research Ethics Committees of the University of the Witwatersrand, Johannesburg (clearance Number M190658), and Oyo State Ministry of Health (Ref. Number AD/13/479/1386).

Data collection was done using a 112-item tool with 9 sections developed in REDCap.[17] Two research assistants directly administered the questionnaire. The first part of the instrument assessed health providers' perceptions of women's rights during childbirth, their awareness of women mistreatment during childbirth in their health facilities, and their awareness of the RMC concept. A one-page brief on 'RMC during childbirth' was read to each respondent (see Additional file 1). The subsequent sections of the questionnaire evaluated providers' perceptions of individual and organisational readiness for change to RMC practice during childbirth, and possible associated factors, using standardised tools.

The respondents' perceived organisational readiness and individual readiness for change to RMC practice were the outcome variables. Organisational readiness for change to RMC (ORC_{RMC}) was assessed using a 12-item tool with 5 items measuring their change commitment and 7-items assessing their change efficacy, both on a 5-point Likert agreement scale. The questions assessing organisational readiness were framed as, *"The health workers in this health facility are…"* Organisational readiness was determined as the mean score of the 12 items on the scale with a maximum score of 5. Individual readiness for change to RMC (IRC_{RMC}) was measured using a 6-item tool on a 5-point Likert agreement scale. Questions were framed as *"I am willing to…"*. IRC_{RMC} was determined as the mean score of the 6-item scale, also with a

For peer review only - http://bmjopen.bmj.com/site/about/guidelines.xhtml

maximum score of 5. When reported as percentages, the mean scores were standardised and converted to it using the formula (Mean-1)/4*100.

For the predictors, Weiner[6] theorised that employee change valence (how much they value the change) and informational assessments (perceived adequacy of the resources available to implement the change) would positively influence organisational readiness for change. Vakola et al[8] further postulated that employee characteristics such as their job satisfaction and core self-evaluation (which assesses their self-esteem, locus of control, emotional stability and generalised self-efficacy)[19] would positively influence their individual readiness for change. We evaluated the relationship between all of these factors with both IRC_{RMC} and ORC_{RMC}. We also proposed that individual provider characteristics such as being younger, having more years of experience, and higher monthly income could positively influence IRC_{RMC} and ORC_{RMC}. We suggested that health providers' perceptions about women's rights during childbirth, their perceived availability and adequacy of resources for RMC implementation and differences in their workplace contexts might influence both IRC_{RMC} and ORC_{RMC}. Additional file 2 shows the list of the standardised tools used to assess the analytical constructs, together with their reliability statistics in our study. The highest Cronbach's alpha was 0.949 for the organisational readiness for change tool, while the lowest was 0.575 for the tool assessing providers' perception of women's rights.

Data analysis was done using the Stata version 15 software. We adjusted for weighting and facility-level clustering in all analyses using the Stata 'svy' commands. The mean scores of the outcome and predictor variables were determined. Higher mean scores indicate higher IRC_{RMC} and ORC_{RMC}. Pearson's correlation was used to evaluate the relationship between IRC_{RMC} and ORC_{RMC}.

Principal component analysis (PCA) was used to construct separate composite indices for the study-specific tools assessing providers' perceptions of women's rights, their awareness of

For peer review only - http://bmjopen.bmj.com/site/about/guidelines.xhtml

mistreatment in their facilities, and the availability of resources for RMC practice. Details are provided in Additional file 3. The first components explained 17.9%, 23.2% and 16.5% of the variance for each of these scales respectively. These PCA scores were then used in the bivariate and multiple regression analysis as potential predictors.

Simple linear regression was done to assess the bivariate relationship between the two numerical outcomes and the predictor variables. Predictors with p-value ≤0.2 were included in the final multiple regression models for each outcome variable. All predictors were added simultaneously. Multicollinearity analysis was conducted after the regressions. Predictor variables with a high variance inflation factor (>10.0) were excluded from the model.

1.2.1. Profile Patient and Public Involvement

A prior qualitative study of pregnant women's perceptions of RMC[18] informed this study and many of the variables assessed. The women had described their experience of childbirth care and queried the readiness of the health providers to provide such care.

1.3. Results

1.3.1. Socio-demographic profile

Two hundred and twelve health providers completed the survey, with the breakdown by professional group as shown in Table 1. Their overall mean age was 44.0. The doctors were the youngest with a mean age of 38.9 while the health auxiliaries were the oldest with a mean age of 49.3. Overall, the respondents had an average of >15 years post-training work experience, which included an average of about 6 years working at the study facility.

For peer review only - http://bmjopen.bmj.com/site/about/guidelines.xhtml

Table 1: Providers' socio-demographic profile by provider type

Variables	Doctor n=38	Nurse n=128	CHEW/CHO n=29	Auxiliary n=18	Total n=212
**Age**					
Mean ± SD	38.9 ±9.9	44.6 ± 9.4	44.5 ± 9.7	49.3 ± 10.5	44.0 ± 9.9
Median (IQR)	40 (31- 46)	44 (39 - 52)	46 (39 - 50)	52 (40 - 56)	44 (38 - 52)
**Sex**					
Male	20 (52.3)	0 (0.0)	2 (8.5)	0 (0.0)	22 (10.4)
Female	18 (47.7)	127 (100.0)	26 (91.5)	18 (100.0)	190 (89.6)
**Type of health facility**					
Primary health facility	3 (8.7)	9 (7.1)	29 (100.0)	18 (100.0)	59 (27.9)
Secondary health facility	34 (91.3)	119 (92.9)	0 (0.00	0 (0.0)	153 (72.1)
**LGA**					
Ibadan North	23 (61.9)	61 (47.8)	3 (12.0)	2 (9.7)	89 (42.2)
Ibadan North East	5 (14.6)	14 (11.2)	5 (16.2)	2 (8.8)	26 12.3)
Ibadan North West	6 (15.6)	20 (15.9)	12 (41.6)	3 (15.0)	41 (19.3)
Ibadan South East	1 (2.9)	1 (0.8)	4 (14.8)	7 (41.8)	14 (6.6)
Ibadan South West	2 (5.1)	31 (24.3)	4 (15.3)	4 (24.7)	42 (19.7)
**Health facility in LGA**					
Facility 1	1 (2.9)	2 (1.4)	3 (12.0)	2 (9.7)	8 (8.7)
Facility 2	23 (59.6)	59 (46.4)	0 (0.0)	0 (0.0)	82 (38.5)
Facility 3	0 (0.0)	2 (1.2)	5 (16.2)	2 (8.8)	9 (3.7)
Facility 4	5 (44.6)	13 (10.0)	0 (0.0)	0 (0.0)	18 (8.6)
Facility 5	1 (3.6)	1 (1.1)	12 (41.6)	3 (15.0)	17 (8.2)
Facility 6	5 (12.0)	19 (14.8)	0 (0.0)	0 (0.0)	23 (11.1)
Facility 7	0 (0.0)	3 (2.6)	5 (15.3)	4 (24.7)	12 (5.7)
Facility 8	2 (5.1)	28 (21.7)	0 (0.0)	0 (0.0)	30 (13.9)
Facility 9	1 (2.3)	1 (1.0)	4 (14.8)	7 (41.8)	13 (6.6)
**Years of experience**					
Mean ± SD	10.4 ± 7.7	18.2 ± 9.9	10.5 ± 7.5	13.6 ± 11.0	15.4 ± 9.9
Median (IQR)	10 (3 - 14)	18 (11 - 25)	8 (4 - 17)	9 (6 - 190)	14 (7 - 23)
**Years working in study facility**					
Mean ± SD	3.2 ± 3.5	8.2 ± 6.0	2.7 ± 1.9	3.7 ± 2.0	6.0 ± 5.6
Median (IQR)	2 (0.5 -5)	7 (4 - 11)	3 (1 - 4)	4 (3 - 5)	5 (2 - 10)
**Income (in USD)**					
Median (IQR)	658 (526 - 921)	500 (289 – 553)	270 (132 -395)	99 (26 – 191)	463(263 – 605)

Note: IQR – Interquartile range

1.3.2. RMC- women's rights and mistreatment and needed resources

Overall, 35.9% of the providers had heard of RMC. This consisted mainly of the doctors (60%) and the least (19.1%) being the health auxiliaries. Nonetheless, after RMC had been explained to them, 70% of all the providers agreed that RMC could be implemented in their facilities.

As shown in Figure 1, 72.9% of the health providers stated that women delivering in their facility were always denied a birth companion, 63.9% were aware of women not being allowed to decide their birth position, and 36.7% had witnessed restrictions on mobility during labour. Correspondingly, only 19.9% of health providers believed that women should always have the right to decide their birth position, 38.7% agreed that women could be mobile during labour,

For peer review only

For peer review only - http://bmjopen.bmj.com/site/about/guidelines.xhtml

and 50.7% supported women having a birth companion (Figure 1). Only 20.4% accepted that women should always have unrestricted access to their hospital records.

Figure 1: Forms of mistreatment and perceived providers' rights to women during childbirth

Figure 2 indicates providers' perceptions of the availability of essential 18 WHO-recommended resources for implementing RMC. The least available of the resources were RMC educational materials (7.7%), followed by guidelines (8.2%). Approximately 10-15% of the providers agreed to the availability of private spaces to support birth companions, in-service training on RMC, suggestion boxes, and adequately trained staff on RMC. However, 63.0% of them agreed to having curtains and screens for privacy during childbirth.

Figure 2: Provider perceptions on availability of WHO-recommended resources for RMC implementation

The mean scores for all the study scales are shown in Table 2. The health providers were well aware of the mistreatment of women during childbirth in their health facilities across the 12 items with a high mean score of 3.9±0.5 out of a maximum of 5. However, the mean score of 3.9±0.5 out of 5 also indicates high acceptance of the rights they believe women should always be granted during childbirth.

1.3.3. Individual and organisational readiness for change to RMC practice

In assessing organisational readiness for change to RMC, the health providers scored high on their commitment to the change and their change efficacy, which is their perceived ability to implement the change (Table 2). These two constructs were strongly positively correlated (rho: 0.830, $p<0.001$). Combined, this gave a high mean organisational readiness for change (ORC_{RMC}) score of 4.01±0.9, which is 75.3% of the maximum obtainable mean score of 5. The health providers had even higher individual readiness to change (IRC_{RMC}), with a mean score of 4.23±0.6, 80.8% of the maximum. Organisational readiness was only moderately but significantly correlated with individual readiness for change to RMC (rho: 0.407, $p<0.001$).

For peer review only - http://bmjopen.bmj.com/site/about/guidelines.xhtml

Table 2: Average provider perceptions for different study scales (n=212)

Analytical Category	Scale	Mean ± SD	95% CI
**Outcomes**	Change commitment	4.05 ± 1.0	3.8– 4.3
	Change efficacy	3.96 ± 0.9	3.6 – 4.3
	Organisational readiness for change (ORC_{RMC})	4.01 ± 0.9	3.7 – 4.3
	Individual readiness for change (IRC_{RMC})	4.23 ± 0.6	4.1 – 4.4
**Predictors**	Awareness of mistreatment during childbirth in their facilities	3.90 ± 0.5	3.7 – 4.1
	Women's rights during childbirth	3.85 ± 0.5	3.8 - 4.0
	Change valence	4.46 ± 0.8	4.3 – 4.6
	Informational assessments	3.30 ± 0.7	3.1 - 3.4
	Availability of resources to implement RMC in their facilities	2.70 ± 0.6	2.5 – 2.9
	Core self-evaluation	4.34 ± 0.5	4.3 – 4.4
	Job satisfaction	3.70 ± 0.6	3.6 – 3.8

1.3.4. Change valence and informational assessments

The health providers scored high on how much they value the change to RMC, with a mean of 4.46±0.8 out of 5 (Table 2). They, however, scored lower in their informational assessments ((3.30±0.7), which describes their perceptions on the adequacy of the available resources to implement the change to RMC practice in their facilities. The providers' mean score for the availability of the WHO-recommended resources to implement RMC was even lower (2.70±0.6), 42.5% of the maximum. There was a mild but significantly positive relationship between their perceived availability and adequacy of the resources needed to implement RMC in their facilities, (rho: 0.263, p=0.0001). Notwithstanding these perceived deficiencies, the health providers indicated relatively high levels of job satisfaction and core self-evaluation- that is, they had high self-esteem, locus of control, emotional stability and generalised self-efficacy (Table 2).

1.3.5. Factors associated with individual readiness for change (IRC_{RMC}) and organisational readiness for change (ORC_{RMC}) to RMC practice

Table 3 shows the bivariate and multiple regression analysis for IRC_{RMC}, while Table 4 shows the analysis for $ORC_{RMC.}$ The health providers' change valence and informational assessments were significantly associated with individual readiness for change in the multiple regression analysis, increasing IRC_{RMC} scores by 0.45 and 0.07 respectively. Doctors and nurses had

For peer review only - http://bmjopen.bmj.com/site/about/guidelines.xhtml

significantly higher IRC_{RMC} than health assistants, in the bivariate analysis but this was no longer significant after adjusting for other covariates.

IRC_{RMC} varied significantly between health providers from different health facilities in the bivariate analysis but this was no longer the case in the multiple regression analysis. None of the known predictors of individual readiness for change (providers' job satisfaction and core self-evaluation), nor the newly proposed ones (perceived rights of women, years of experience, income), was significantly associated with IRC_{RMC}.

For peer review only

For peer review only - http://bmjopen.bmj.com/site/about/guidelines.xhtml

Table 3: Analysis of factors associated with health providers' IRC_{RMC}

Covariates	Simple linear regression			Multiple linear regression		
	**Crude Coeff.**	**95%CI**	**p-value**	**Adjusted Coeff.**	**95%CI**	**p-value**
Health providers' age	-0.003	-0.01 - 0.007	0.497			
**Sex**						
Female	Ref	-	-	Ref	-	-
Male	-0.21	-0.02 – 0.44	0.065	-0.14	-0.84 – 1.13	0.743
**Study Local Government Area**						
Ibadan North	Ref	-	-			
Ibadan North East	0.02	-0.54 – 0.58	0.946			
Ibadan North West	-0.22	-0.47 – 0.03	0.078			
Ibadan South East	-0.45	-052 – 0.37	**<0.001**			
Ibadan South West	-0.09	-0.32 – 0.14	0.383			
**Health facility in LGA**						
Facility 1	0.19	0.10 – 0.19	<0.001	0.35	-0.01 – 0.70	0.053
Facility 2	Ref	-	-	Ref	-	-
Facility 3	-0.53	-0.53- -0.53	<0.001	-0.06	-0.39 – 0.27	0.675
Facility 4	0.29	0.29 – 0.29-	<0.001	0.07	-0.05 – 0.18	0.218
Facility 5	-0.37	0.37 - -0.37	<0.001	-0.23	-0.51 – 0.04	0.087
Facility 6	-0.06	-0.06 - -0.06	<0.001	0.04	-0.07 – 0.14	0.423
Facility 7	0.12	0.12 – 0.12	<0.001	0.22	-0.13 - 0.54	0.175
Facility 8	-0.10	-0.10 – -0.10	<0.001	0.0002	-0.09 – 0.09	0.970
Facility 9	-0.42	-0.42 - -0.42	<0.001	0.03	-0.30 – 0.35	0.844
**Providers' type of health facility**						
Primary health facility	Ref	-	-			
Secondary health facility	0.23	0.56 – 0.09	0.135			
**Professional cadre**						
Doctor	0.43	0.04 – 0.83	**0.036**	-0.13	-0.46 – 0.19	0.360
Nurse	0.37	0.05 – 0.70	**0.030**	Ref	-	-
CHEW/ CHO	0.08	-0.06 – 0.21	0.233	-0.19	-0.63 – 0.24	0.329
Health Assistant/ Aide	Ref	-	-	-0.16	-0.50 – 0.18	0.296
Income (in USD/ 1000)	-2.36	-0.06 – 5.28	0.097	0.05	-0.03 - 0.13	0.187
Years of professional experience	0.01	0.004 – 0.03	0.106	0.004	-0.02 – 0.03	0.644
Years of experience in health facility	0.004	-0.02 – 0.031	0.727			
Awareness of mistreatment of women	0.01	-0.04 – 0.05	0.712			
Perceived women's rights during childbirth	0.04	-0.05 – 0.11	0.357			
**Ever heard of RMC (n=170)**						
Yes	-0.02	-0.33 – 0.30	0.883			
No	Ref	-	-			
**Perception of RMC being implementable**						
Agreed	0.10	-0.34 – 0.53	0.620			
Indifferent	Ref	-	-			
Disagreed	0.06	-0.46 – 0.58	0.794			
Change valence (value for RMC practice)	0.45	0.19– 0.71	**0.005**	0.40	0.11 – 0.70	**0.015**
RMC Informational assessment	0.07	0.15 - 0.42	**0.001**	0.07	0.008 – 0.13	**0.032**
Provider perceptions on available resources	0.03	-0.02 – 0.09	0.182			
Provider job satisfaction	0.010	-0.03 – 0.22	0.105	0.004	-0.13 – 0.14	0.953
Provider core self-evaluation	0.25	0.01 – 0.50	0.055	0.09	-0.22– 0.39	0.513
*Male # Doctor				0.15	-1.01 – 1.31	0.765
Constant				1.76	1.37 – 2..14	**<0.001**

n=212; R^2= 0.4363; p<0.001

Note: Ref: means the reference category; 95%CI: 95% Confidence Interval; Predictors with p-value ≤0.2 from the simple linear regression analysis were included in the multiple regression model; The Mean variance inflation factor vif for the multiple regression model is =2.33, significant p-values in bold. Male # Doctor- Interaction between gender and profession

For peer review only - http://bmjopen.bmj.com/site/about/guidelines.xhtml

For peer review only

Change valence and informational assessments were also significantly associated with organisational readiness for change (Table 4). A unit increase in the health providers' change valence and informational assessments increased their perceived ORC_{RMC} by 0.47 and 0.43 units respectively, after adjusting for other covariates. Also, each additional 10 years of work experience significantly increased ORC_{RMC} by 0.08 and each $1000 increase in providers' personal income increased their perceived ORC_{RMC} by 0.08. There were significant varied associations (positively or negatively) between the health providers' facility of practice and their ORC_{RMC} in relation to the reference facility. The only exception was for Facility 4, a secondary health facility in one of the LGAs.

For peer review only - http://bmjopen.bmj.com/site/about/guidelines.xhtml

Table 4: Analysis of factors associated with health providers' ORC_{RMC}

Covariates	Simple linear regression			Multiple regression		
	Crude Coeff.	95%CI	p-value	Adjusted Coeff.	95%CI	p-value
Health providers' age	-0.01	-0.02 – 0.02	0.916			
**Sex**						
Female	Ref	-	-	Ref	-	-
Male	-0.21	-0.09 – 0.50	0.146	0.15	-0.11 – 0.41	0.213
**Study Local Government Area**						
Ibadan North	Ref	-	-			
Ibadan North East	0.34	-0.26 – 0.94	0.226			
Ibadan North West	-0.22	-0.42 – 0.02	**0.034**			
Ibadan South East	-0.46	-0.59 – 0.33	**<0.001**			
Ibadan South West	-0.27	-1.21 – 0.66	0.510			
**Health facility in LGA**						
Facility 1	0.43	0.43 – 0.43	**<0.001**	0.38	0.30 – 0.46	**<0.001**
Facility 2	Ref	-	-	-	-	-
Facility 3	-0.23	-0.23 - -0.23	**<0.001**	0.24	0.12 – 0.35	**0.002**
Facility 4	0.65	0.65 – 0.65	**<0.001**	0.16	-0.03 – 0.35	0.087
Facility 5	-0.09	-0.09 - -0.09	**<0.001**	-0.29	-0.40 - -0.19	**<0.001**
Facility 6	-0.24	-0.24 - -0.24	**<0.001**	-0.11	-0.16 - -0.07	**0.001**
Facility 7	0.63	0.63 – 0.63	**<0.001**	0.56	0.54 – 0.57	**<0.001**
Facility 8	-0.54	-0.54 - -0.54	**<0.001**	-0.41	-0.47- -0.36	**<0.001**
Facility 9	-0.41	-0.41 - -0.41	**<0.001**	0.11	0.02 – 0.20	**0.024**
**Providers' type of health facility**						
Primary health facility	Ref	-	-			
Secondary health facility	0.09	-0.68– 0.50	0.717			
**Professional cadre**						
Doctor	0.31	-0.50 – 1.12	0.391			
Nurse	-0.07	-0.88 – 0.75	0.857			
CHEW/ CHO	0.09	-0.38 – 0.56	0.667			
Health Assistant/ Aide	Ref	-	-			
Income (in USD/ 1000)	0.26	-0..05- 0.56	0.083	0.08	0.02– 0.15	**0.021**
Years of professional experience /10 years	0.05	0.02 – 0.3	**0.034**	0.08	0.01 – 0.2	**0.024**
Years of experience in health facility	-0.004	-0.03 – 0.02	0.678			
Awareness of mistreatment of women	0.02	-0.06 – 0.09	0.650			
Perceived women's rights during childbirth	0.02	-0.12 – 0.16	0.767			
**Ever heard of RMC (n=170)**						
Yes	0.10	-0.27 – 0.46	0.553			
No	Ref	-	-			
**Perceptions of RMC being implementable**						
Agreed	0.60	-0.02 – 1.23	0.056	0.19	-0.08– 0.45	0.148
Indifferent	Ref	-	-	Ref	-	-
Disagreed	-0.09	-0.76 – 0.58	0.765	-0.12	-0.60 – 0.36	0.570
Change valence (value for RMC practice)	0.74	0.47 – 1.01	**<0.001**	0.47	0.21 – 0.74	**0.004**
RMC Informational assessment	0.72	0.40 – 1.05	**0.001**	0.43	0.22 – 0.63	**0.002**
Provider perceptions on available resources	-0.002	-0.25 – 0.25	0.984			
Provider job satisfaction	0.23	-0.08 – 0.55	0.125	0.05	-0.10 – 0.20	0.477
Provider core self-evaluation	0.15	-0.38 – 0.68	0.521			
Constant				0.06	-1.28 – 1.41	0.915

n=212; R^2= 0.6016; p<0.001

Note: Ref: means the reference category; CI: 95% Confidence Interval; Predictors with a p-value ≤0.2 from the bivariate analysis (simple linear regression) were included in the multiple regression model; The Mean variance inflation factor vif for the multiple regression model is =1.55, Significant p-values in bold.

For peer review only

1.4. Discussion

This is the first study to explore individual and organisational readiness for change to RMC practice, and associated predictors. The health providers had a high level of awareness of mistreatment to women but also a high general acceptance of women's rights during childbirth. However, there were some rights, such as being allowed a birth companion, that few providers regarded as essential, and these were then seldom practised. Nonetheless, the health providers scored high in their perceived IRC_{RMC} and ORC_{RMC}. IRC_{RMC} and ORC_{RMC} were only moderately correlated in this analysis. Higher change valence and informational assessment of the adequacy of resources increased not only ORC, as has been found previously,[20,21] but also IRC_{RMC}. Job satisfaction and the providers' core self-evaluation, which have been shown to influence IRC,[8,22] had no statistically significant effect on IRC_{RMC} in this study. The provider's years of work experience, their personal income (individual characteristics) and their health facility of practice (a workplace characteristic) significantly influenced ORC_{RMC}.

This study has provided an understanding of the state of readiness for change to RMC practice, eliminating it as a possible implementation problem for RMC practice in the study setting. It has established that IRC_{RMC} and ORC_{RMC} have a positive influence on each other. This study has further confirmed the critical role of change valence and informational assessments in increasing both organisational and individual readiness for change.

The study findings however failed to establish a significant relationship between the providers' readiness for a change to RMC and their perceptions of women's rights during childbirth. Respectful maternity care is premised on the fundamental human rights of women to receive dignified care.[23] It would have been expected that provider perceptions of women's rights would be positively associated with their readiness for change. The relationship was in the correct direction but not statistically significant. The provider's low perceptions of resource availability to implement RMC did also not significantly reduce their IRC_{RMC} and ORC_{RMC}.

For peer review only - http://bmjopen.bmj.com/site/about/guidelines.xhtml

This study had some limitations. It was a relatively small study and its geographical extent was limited to one Metro in Nigeria which may not be representative of similar facilities and providers in other regions of Nigeria. Tertiary health facilities were not included because there was only one tertiary health facility serving populations across the five LGAs studied. Social desirability bias may have influenced some of the providers' responses positively to the availability of resources and their perception of women's rights during childbirth. To mitigate this, the data collectors stressed the academic purpose of the research to the providers when obtaining informed consent. Limited awareness of RMC, as found in this study, may affect an accurate assessment of readiness for change. We attempted to address this by educating the providers on RMC concepts before assessing their readiness for change to RMC practice.

Health providers cannot truly be ready to implement RMC if they do not support certain women's rights during childbirth. This would result in persistent mistreatment and may prevent a positive change to RMC practice. The most common forms of mistreatment to women during childbirth in the study health facilities were being denied birth companions, not being allowed to decide on birth position, and being denied mobility in labour. All three forms of mistreatment were also reported by Tanzanian women in a qualitative study of the perspectives of mothers and fathers on mistreatment during childbirth.[24] Several other studies have reported these forms of mistreatment experienced by women during childbirth.[25–28] According to the WHO,[29,30] having a birth companion during labour provides emotional support, reduces labour pain and strengthens the woman's capability to deliver. The WHO has also recommended that women are supported to deliver in their preferred birth position because alternative birth positions, such as standing to deliver, are safe and may result in shorter labour from better foetal alignment.[27,29] It has also been reported that mobility during the first stage of labour is safe.[27] Denying women the autonomy, or not respecting women's choices during childbirth without a justifiable medical reason, constitutes mistreatment that negatively affects their overall childbirth experience.[31]

For peer review only - http://bmjopen.bmj.com/site/about/guidelines.xhtml

356 The health providers perceived that women should always have the right to full information about their care and to receive their care in privacy. Unfortunately, many may not practice it for several reasons, including unconscious behaviour, an abusive work culture, and perceived excessive workload amongst others.[32] About 33% of maternity care providers in Western Kenya attested that they do not often give explanations before conducting procedures on women during childbirth, and 73% do not wait to obtain consent before conducting these examinations.[32] This is similar to the inconsistent support for women's right to autonomy found among Australian midwives and doctors.[33] They confirmed their support for women's autonomy, but override women's decisions sometimes on safety reasons, claiming full accountability for every pregnancy outcome. Women should be included when safety decisions are being made during childbirth. When this is not done, women may conclude it is an abuse of their rights. Tanzanian women related their abusive maternity care experiences as a deviation from their basic human rights.[34] Hence, advocating for women's rights among health providers should be a key component of RMC-promoting interventions.

370 Nonetheless, the health providers scored high in their perceived IRC_{RMC} and ORC_{RMC}. Few studies had reported the overall organisational readiness for implementing change (ORIC) in health programmes as mean scores using the ORIC tool. Many either report the mean change commitment and change efficacy as individual scores,[15] or as total scores.[35] The ORC_{RMC} score in our study was higher than the average of the change commitment and change efficacy scores found when the nurse-reported ORC for policy change in acute care hospitals in Switzerland was assessed.[15] There was no comparable study of individual readiness for change using the same instrument applied in health industry. A scoping review to explore the nature and extent of literature published on individual readiness for change in the health sector yielded no study found in health.[36]

For peer review only - http://bmjopen.bmj.com/site/about/guidelines.xhtml

IRC_{RMC} and ORC_{RMC} in our study were significantly positively correlated. Thus, a positive increase in IRC_{RMC} by strengthening its facilitating factors should also reflect in increased ORC_{RMC}. This is similar to the postulations by Weiner in his theory where he stated that "*Organisational readiness is likely to be highest when organisational members not only want to implement an organisational change but also feel confident that they can do so*" (page 3).[6]

Weiner theorised that organisational readiness was most strongly influenced by change valence and informational assessments.[6] The health providers' change valence positively influenced both their IRC_{RMC} and ORC_{RMC} significantly in our study. Change valence also positively and significantly influenced organisational readiness for change amongst employees of a private hospital changing to a tertiary hospital.[37] It also strongly correlated with individual readiness for change in an automobile industry.[38] There has been limited assessments of individual readiness for change in health-related industries.

Informational assessment is the perceived adequacy of the available resources such as the equipment, expertise, skills, and time, needed to implement the change. Informational assessments also significantly influenced both IRC_{RMC} and ORC_{RMC} in this analysis. Informational assessment of their perceived resource adequacy was found to be positively and significantly correlated with their perceived resource availability in this study. This suggests that if providers' perception of resource availability is high, they would be readier for a change to RMC practice. However, the providers had a low perception of the availability of recommended resources for RMC implementation in our study setting. This may have explained their fairly low perceived resource adequacy.

Thus, additional resource requirements are critical drivers of RMC implementation.[6] For example, only 9% of the health providers agreed that facilities to support birth companions were available. This would include a private space achievable with the use of curtains. In an observational study of childbirths across four countries, Nigeria had the lowest proportion of

For peer review only

For peer review only - http://bmjopen.bmj.com/site/about/guidelines.xhtml

women (6.9%) in which curtains were used to ensure privacy.[39] This is a challenge that may prevent Nigerian women from receiving RMC as there is limited funding to the Nigerian health system to provide these essential RMC resources. There is a need to identify cost-effective strategies to address these system challenges.

ORC_{RMC} was found to be significantly higher among health providers with longer years of work experience. They are a population to target in RMC-promoting interventions. The nurses' years of work experience also positively influenced their change commitment, one of the measures of organisational readiness, in Switzerland's acute care hospitals.[15] The providers' workplace setting, as indicated by their health facility of practice significantly influenced their perceived $ORC_{RMC.}$ This was positive for most of the primary health care facilities across the LGAs, and was significantly negative for the two secondary health facilities studied. Interestingly, both the primary and secondary health facilities in the Ibadan North-west LGA were significantly associated with a decreased $ORC_{RMC.}$ According to the literature, the workplace contextual fit is critical to providers' readiness for change to RMC as it informs the adaptability of the local context to the globally defined RMC practice, the quality of the implementation, and whether expected RMC implementation outcomes will be achieved.[40–42] There is the need to qualitatively explore which contextual factors within the health facilities are the most critical barriers to a successful implementation of RMC practice during childbirth.

1.5. Conclusions

The three most common forms of mistreatment during childbirth noted by health providers corresponded with the low recognition of these as rights that women should always receive. Our study confirmed the relevance of the organisational and individual readiness for change constructs to the RMC literature and should prompt more studies on this topic. It is noteworthy that the health providers in our study perceived themselves and their organisations to be ready for a change to RMC practice. It would be important to verify in future research if readiness for

For peer review only

change significantly facilitated the implementation of RMC interventions. The main influencing factors of both IRC_{RMC} and ORC_{RMC} scores in our analysis were a high valuation of the change (change valence) and the perceived adequacy of resources necessary to implement the change. Longer serving providers may be a readier population to target during RMC implementation either as champions to lead a change to RMC practice. Workplace contexts could significantly influence ORC_{RMC} and should be explored before the implementation of RMC interventions.

Ethics approval and consent to participate: The research was conducted in accordance with the Declaration of Helsinki. Ethical approvals were obtained from the Human Research Ethics Committees (HRECs) of the University of the Witwatersrand, Johannesburg (clearance Number M190658), and the Oyo State Ministry of Health (Ref. Number AD/13/479/1386). A written consent to continue the interview was obtained from the respondents. It was a consent form that explained the purpose of the research, and the respondents were asked if they agree to continue with the research or not. The form was filled using the REDCap software. For anonymity, the facilities were referred to by numbers rather than names. The primary and secondary health facilities were designated with odd and even numbers respectively. Subsequent numbers in sequence are located in the same LGA. Respondents were given a small jotter with brief information on respectful maternity care that costs 0.32USD at ₦380=1USD each on completion of the survey in appreciation of their time and to further educate them. There were no inducements given before participation. A transparent and complete reporting of the research was done guided by the STROBE's checklist.[43].

Availability of data and materials:

All dataset generated and analysed in the current study are available from the Figshare database accessible at https://figshare.com/s/4c3a01159121780b77da. The doi is 10.6084/m9.figshare.19757329. Also the datasets used and analysed during the current study

For peer review only - http://bmjopen.bmj.com/site/about/guidelines.xhtml

and on which the study findings and conclusions are based are available with the corresponding author and will be shared on reasonable request.

Competing Interests

The authors declare that they have no competing interests

Funding

This research was supported by the Consortium for Advanced Research Training in Africa (CARTA). CARTA is jointly led by the African Population and Health Research Centre and the University of the Witwatersrand and funded by the Carnegie Corporation of New York (Grant No G-19-57145), Sida (Grant No:54100113), Uppsala Monitoring Center, Norwegian Agency for Development Cooperation (Norad), and by the Wellcome Trust [reference no. 107768/Z/15/Z] and the UK Foreign, Commonwealth & Development Office (No Grant Number), with support from the Developing Excellence in Leadership, Training and Science in Africa (DELTAS Africa) programme (No Grant Number). The statements made and views expressed are solely the responsibility of the Fellow. For the purpose of open access, the author has applied a CC BY public copyright licence to any 'author accepted' manuscript version arising from this submission. The funders only provided the funding for the research. They played no role in the conceptualisation, design and conduct of the research nor in the data analysis, interpretation and development of the manuscript for publication.

Author Contributions

OTE conceptualised the study, designed the study, acquired and adapted the tools to the study and obtained the ethical approvals. She was the principal investigator who conducted the data collection and supervised the research assistants. She analysed the data, and wrote the draft and final manuscripts for publication.

SM contributed to the design of the study and the finalisation of the tools. She also significantly contributed to the revision of the draft manuscript and approved the final manuscript for publication.

DB contributed significantly to the design of the study, the finalisation of the tools and the data analysis. He significantly revised and contributed significant intellectual content to the draft manuscript and approved the final version of the manuscript for publication.

Acknowledgements

I acknowledge the contributions of Dr Tunde Adedokun, Dr Joshua Akinyemi and Professor Folusho Owotade for their inestimable advice and support with the data analysis. I also recognise and appreciate all the health providers who completed the survey despite their hectic schedules. The second author acknowledges the funding support by the South African Medical Research Council's Mid-Career Scientist Award.

References

1. World Health Organization. WHO recommendations: Intrapartum care for a positive childbirth experience [Internet]. Geneva, Switzerland; 2018 [cited 2018 Aug 8]. 1–200 p. Available from: https://www.who.int/reproductivehealth/publications/intrapartum-care-guidelines/en/

2. Williams CR, Meier BM. Ending the abuse: the human rights implications of obstetric violence and the promise of rights-based policy to realise respectful maternity care. Sex Reprod Heal Matters. 2019 Jan 1;27(1).

3. Denny E. Mini commentary: Respectful maternity care needs to be the standard for all women worldwide. BJOG [Internet]. 2018 [cited 2018 May 10];125(8):943. Available from: https://pubmed.ncbi.nlm.nih.gov/29230930/

4. Downe S, Lawrie TA, Finlayson K, Oladapo OT. Effectiveness of respectful care policies for women using routine intrapartum services : a systematic review. Reprod Health. 2018;15(23):1–13.

5. Holt DT, Armenakis AA, Harris SG, Feild HS. Toward a Comprehensive Definiton of Readiness for Change: A Review of Research and Instrumentation. Res Organ Chang Dev [Internet]. 2015 [cited 2019 May 23];16:289–336. Available from: https://www.emeraldinsight.com/doi/abs/10.1016/S0897-3016%2806%2916009-7

6. Weiner BJ. A theory of organizational readiness for change. Implement Sci [Internet]. 2009 [cited 2018 Aug 9];4(67):1–9. Available from: http://www.implementationscience.com/content/4/1/67

7. Lehman WEK, Greener JM, Simpson DD. Assessing organizational readiness for change. J Subst Abuse Treat. 2002;22(4):197–209.

8. Vakola M. What's in there for me? Individual readiness to change and the perceived impact of organizational change. Leadersh Organ Dev J. 2014;35(3):195–209.

9. Miake-Lye IM, Delevan DM, Ganz DA, Mittman BS, Finley EP. Unpacking

For peer review only - http://bmjopen.bmj.com/site/about/guidelines.xhtml

organizational readiness for change: An updated systematic review and content analysis of assessments. BMC Health Serv Res. 2020;20(1):1–13.

10. Haffar M, Al-Karaghouli W, Irani Z, Djebarni R, Gbadamosi G. The influence of individual readiness for change dimensions on quality management implementation in Algerian manufacturing organisations. Int J Prod Econ [Internet]. 2019 [cited 2021 Aug 31];207:247–60. Available from: http://dx.doi.org/10.1016/j.ijpe.2016.08.024

11. Warren CE, Ndwiga C, Sripad P, Medich M, Njeru A, Maranga A, et al. Sowing the seeds of transformative practice to actualize women's rights to respectful maternity care: Reflections from Kenya using the consolidated framework for implementation research. BMC Womens Health. 2017;17(1):1–18.

12. Ratcliffe HL, Sando D, Lyatuu GW, Emil F, Mwanyika-sando M, Chalamilla G, et al. Mitigating disrespect and abuse during childbirth in Tanzania : an exploratory study of the effects of two facility-based interventions in a large public hospital. Reprod Health [Internet]. 2016 [cited 2018 Sep 27];13(79):1–13. Available from: http://dx.doi.org/10.1186/s12978-016-0187-z

13. Kujawski SA, Freedman LP, Ramsey K, Mbaruku G, Mbuyita S, Moyo W, et al. Community and health system intervention to reduce disrespect and abuse during childbirth in Tanga Region, Tanzania: A comparative before-and-after study. PLoS Med. 2017;14(7):1–16.

14. Stata Software. Power and Sample size: Power onemean — Power analysis for a one-sample mean test [Internet]. 2019. p. 1–15. Available from: file:///E:/PhD Folder/Respectful maternity care/Very Important for Current PHD Research/psspoweronemean (1).pdf

15. Sharma N, Geest S De, Herrnschmidt J, Claes V, Bachnick S, Simon M. Organizational readiness for implementing change in acute care hospitals : An analysis of a cross - sectional , multicentre study. J Adv Nurs. 2018;74(12):2798–808.

16. Glen S. Design Effect: Definition, Examples [Internet]. Statistics How To. 2015 [cited 2019 Apr 1]. Available from: https://www.statisticshowto.datasciencecentral.com/design-effect/

17. Hameed W, Uddin M, Avan BI. Are underprivileged and less empowered women deprived of respectful maternity care: Inequities in childbirth experiences in public health facilities in Pakistan. PLoS One. 2021;16(4 April):1–17.

18. Esan OT, Maswime S, Blaauw D. A qualitative inquiry into pregnant women's perceptions of respectful maternity care during childbirth in Ibadan Metropolis, Nigeria. Sex Reprod Heal Matters. 2022;30(1):1–17.

19. Judge TA, Erez A, Bono J., Thoresen CJ. The Core Self-Evaluation Scale (CSES): Development of a Measure. Pers Psychol. 2003;56:303–31.

20. Shea CM, Jacobs SR, Esserman DA, Bruce K, Weiner BJ. Organizational readiness for implementing change : a psychometric assessment of a new measure. Implement Sci. 2014;9(7):1–15.

21. Phillips JE. Effects of Change Valence and Informational Assessments on Organizational Readiness for Change T [Internet]. Walden University, Cape Town; 2017 [cited 2018 Sep 27]. Available from: https://scholarworks.waldenu.edu/dissertations/4016/

For peer review only - http://bmjopen.bmj.com/site/about/guidelines.xhtml

22. Win NN, Chotiyaputta V. Measuring Employee Readiness To Change : a Case Study of an Organization in Myanmar. Panyapiwat J. 2018;10(3):110–24.

23. White Ribbon Alliance. Respectful Maternity Care: The Universal Rights of Childbearing Women [Internet]. The White Ribbon Alliance for Safe Motherhood. 2011 [cited 2021 Feb 1]. Available from: https://www.who.int/woman_child_accountability/ierg/reports/2012_01S_Respectful_Maternity_Care_Charter_The_Universal_Rights_of_Childbearing_Women.pdf

24. Mselle LT, Kohi TW, Dol J. Humanizing birth in Tanzania: A qualitative study on the (mis) treatment of women during childbirth from the perspective of mothers and fathers. BMC Pregnancy Childbirth. 2019;19(1):1–11.

25. Balde MD, Nasiri K, Mehrtash H, Soumah AM, Bohren MA, Diallo BA, et al. Labour companionship and women's experiences of mistreatment during childbirth: Results from a multi-country community-based survey. BMJ Glob Heal. 2020;5(e003564):1–10.

26. Afulani P, Kusi C, Kirumbi L, Walker D. Companionship during facility-based childbirth: results from a mixed-methods study with recently delivered women and providers in Kenya. BMC Pregnancy Childbirth 2018 181 [Internet]. 2018 May 10 [cited 2021 Oct 10];18(1):1–28. Available from: https://bmcpregnancychildbirth.biomedcentral.com/articles/10.1186/s12884-018-1806-1

27. Lawrence A, Lewis L, Hofmeyr GJ, Dowswell T, Styles C. Maternal positions and mobility during first stage labour. Cochrane Database Syst Rev. 2013;(8).

28. Lugina H, Mlay R, Smith H. Mobility and maternal position during childbirth in Tanzania: an exploratory study at four government hospitals. BMC Pregnancy Childbirth 2004 41 [Internet]. 2004 Feb 19 [cited 2021 Oct 10];4(1):1–10. Available from: https://bmcpregnancychildbirth.biomedcentral.com/articles/10.1186/1471-2393-4-3

29. World Health Organization. Standards for improving quality of maternal and newborn care in health facilities [Internet]. Geneva,, Switzerland: WHO Press; 2016 [cited 2018 Oct 16]. 84 p. Available from: https://www.who.int/maternal_child_adolescent/documents/improving-maternal-newborn-care-quality/en/

30. WHO | Why having a companion during labour and childbirth may be better for you. WHO [Internet]. 2019 [cited 2021 Oct 10]; Available from: http://www.who.int/reproductivehealth/companion-during-labour-childbirth/en/

31. Mirzaee Rabor F, Taghipour A, Najmabadi KM, Fattahi Masoum SH, Pour MF. Respect to women's autonomy in childbirth: A qualitative study. Iran Red Crescent Med J. 2017;19(12).

32. Afulani PA, Afulani PA, Buback L, Kelly AM, Kirumbi L, Cohen CR, et al. Providers' perceptions of communication and women's autonomy during childbirth: A mixed methods study in Kenya. Reprod Health. 2020 Jun 3;17(1).

33. Edwards N. Maternity care providers' perceptions of women's autonomy and the law. AIMS J. 2015;27(1):22.

34. Miltenburg AS, Lambermon F, Hamelink C, Meguid T. Maternity care and Human

For peer review only - http://bmjopen.bmj.com/site/about/guidelines.xhtml

Rights: what do women think? BMC Int Health Hum Rights [Internet]. 2016 [cited 2018 Jun 16];16(17):1–10. Available from: http://dx.doi.org/10.1186/s12914-016-0091-1

35. Geerligs L, Shepherd HL, Butow P, Shaw J, Masya L, Cuddy J, et al. What factors influence organisational readiness for change? Implementation of the Australian clinical pathway for the screening, assessment and management of anxiety and depression in adult cancer patients (ADAPT CP). Support Care Cancer. 2021;29(6):3235–44.

36. Beasley L, Grace S, Horstmanshof L. Assessing individual readiness for change in healthcare: a review of measurement scales. J Health Organ Manag [Internet]. 2021 Jan 1 [cited 2021 Mar 10];35(8):1062–79. Available from: https://doi.org/10.1108/JHOM-10-2020-0414

37. Trisnawati A, Damayanti N, Novita RD. How Change Valence Impacts Readiness to Change In Teaching Hospital. Eur J Mol Clin Med. 2020;07(05):310–6.

38. AbuTahoun TNB, Khan N. The Management Role in Promoting Individual Readiness for Change. Int Conf Manag Inf Syst [Internet]. 2019 [cited 2021 Jul 28];29:217–22. Available from: https://search.ebscohost.com/login.aspx?direct=true&db=buh&AN=141539752&site=ehost-live

39. Asefa A, McPake B, Langer A, Bohren MA, Morgan A. Imagining maternity care as a complex adaptive system: understanding health system constraints to the promotion of respectful maternity care. Sex Reprod Heal Matters [Internet]. 2020 [cited 2021 Oct 11];28(1):456–74. Available from: https://doi.org/10.1080/26410397.2020.1854153

40. Edwards N, Barker PM. The importance of context in implementation research. J Acquir Immune Defic Syndr. 2014;67:S157–62.

41. Horner R, Blitz C, Ross SW. The Importance Of Contextual Fit When Implementing Evidence-Based Interventions [Internet]. ASPE Issue Brief. 2014 [cited 2021 Nov 27]. Available from: http://aspe.hhs.gov/hsp/14/IWW/ib_Contextual.pdf

42. Kok BC De, Uny I, Immamura M, Bell J, Geddes J, Phoya A. From Global Rights to Local Relationships : Exploring Disconnects in Respectful Maternity Care in Malawi. Qual Health Res. 2020;20(3):341–335.

43. Von Elm E, Altman DG, Egger M, Pocock SJ, Gøtzsche PC, Vandenbroucke JP. The Strengthening the Reporting of Observational Studies in Epidemiology (STROBE) statement: Guidelines for reporting observational studies. PLoS Med. 2007;4(10):1623–7.

For peer review only - http://bmjopen.bmj.com/site/about/guidelines.xhtml

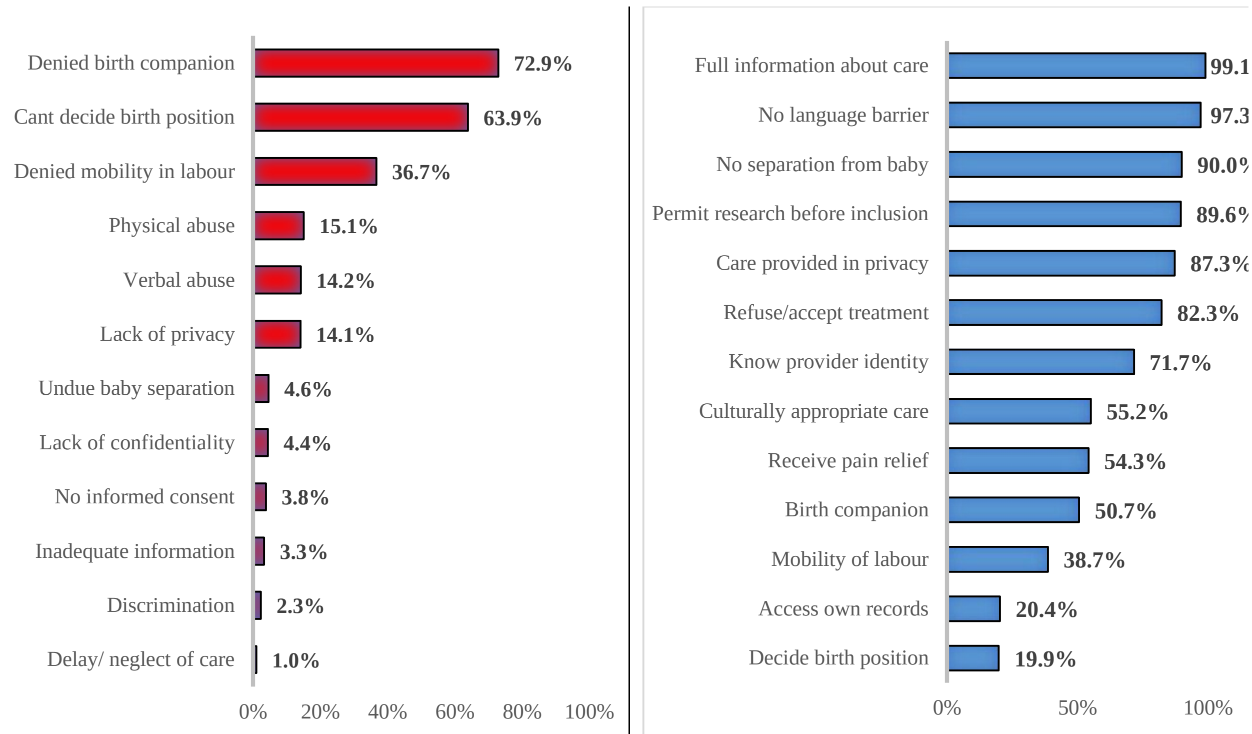

Forms of mistreatment noted by providers in their health facilities (n=212)

Provider's perceived rights women should always have during childbirth (n=212)

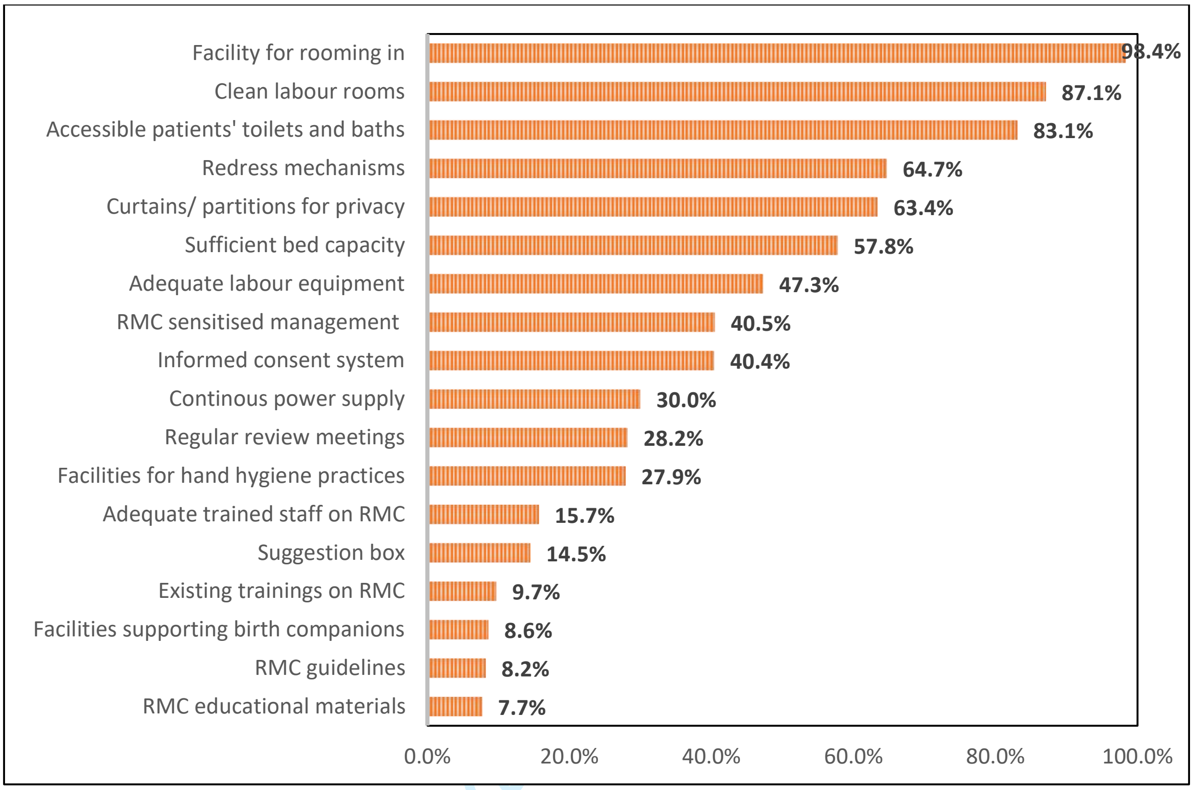
Facility for rooming in
98.4%
Clean labour rooms
87.1%
Accessible patients' toilets and baths
83.1%
Redress mechanisms
64.7%
Curtains/ partitions for privacy
63.4%
Sufficient bed capacity
57.8%
Adequate labour equipment
47.3%
RMC sensitised management
40.5%
Informed consent system
40.4%
Continous power supply
30.0%
Regular review meetings
28.2%
Facilities for hand hygiene practices
27.9%
Adequate trained staff on RMC
15.7%
Suggestion box
14.5%
Existing trainings on RMC
9.7%
Facilities supporting birth companions
8.6%
RMC guidelines
8.2%
RMC educational materials
7.7%
0.0%
20.0%
40.0%
60.0%
80.0%
100.0%

Additional file 1

What is Respectful Maternity Care?

Respectful Maternity Care (RMC) is a human rights approach to childbirth care practice. It is a new strategy for caring for women in labour which we are yet to commence implementing in this health facility. We are interested in knowing how health facilities offering childbirth services, their managers and individual workers are READY to integrate respectful maternity care into their routine childbirth services.

What is Respectful Maternity care?
Simply, it means the following

1. The preferences of the client must be respected, and she must be involved in the decision making regarding her health.
2. She must be allowed a companion during birth as recommended by the WHO
3. She must be free to move about during labour if she so wishes even in the second stage before the urge to deliver and not restricted to one position.
4. If classified as a low risk pregnant woman, she should be allowed oral fluids or food while in labour as evidence has shown no negative outcomes following this.
5. Her privacy must be ensured by providing one private cubicle or space per woman in labour and information about her should not be shared openly.
6. If she prefers to deliver her child squatting, the health care provider must be willing to support her in the decision.
7. Equitable services must be delivered to her regardless of her personal characteristics.
8. When she calls for help during labour, she must not be denied nor neglected.
9. If she is unable to pay her bills, a consensus must be reached with her on how to pay rather than detaining her illegally for the inability to pay.
10. Overall, she must receive the utmost respectful and dignified care, that she deserves as her fundamental human rights.

For peer review only

Additional file 2: Breakdown of tools in the health provider survey instrument

	Name of Tool and Sections	Source	Items	Response type	Alpha coeff.
1	*Organisational Readiness for Implementing change (ORIC)	Shea et al[1]	12	Likert scale 1-5	0.949
2	*Individual readiness for change	Vakola et al[2]	6	Likert scale 1-7	0.733
3	Socio-demographic characteristics	Adapted from the literature	15		
4	Perception on women's rights during childbirth	Childbirth Connection[3]	13	Likert scale 1-5	0.575
5	Provider awareness of mistreatment in their own facility	Maternal & Child Health program[4]	12	Likert scale 1-5	0.638
6	Change valence	Shea et al[1]	6	Likert scale 1-5	0.902
7	Informational assessments	Phillip[5]	8	Likert scale 1-5	0.648
8	Perception on RMC resource availability	WHO Recommendation for labour[6]	18	Likert scale 1-5	0.669
10	Core self-evaluation tool	Judge et al[7]	12	Likert scale 1-5	0.598
11	Employee job satisfaction tool	Management Sciences for Health[8]	10	Likert scale 1-5	0.603
	Total		112		

*Outcome variables; WHO: World Health Organisation

References

1. Shea CM, Jacobs SR, Esserman DA, Bruce K, Weiner BJ. Organizational readiness for implementing change : a psychometric assessment of a new measure. Implement Sci. 2014;9(7):1–15.

2. Vakola M. What's in there for me? Individual readiness to change and the perceived impact of organizational change. Leadersh Organ Dev J. 2014;35(3):195–209.

3. Childbirth Connection. The Rights of Childbearing Women: Fundamental Problems with Maternity Care in the United States [Internet]. ChildBirth Connection. 2006 [cited 2019 Jan 12]. p. 1–4. Available from: www.childbirthconnection.org/rights

4. Maternal and Child Health Integrated Program. Respectful Maternity Care Program Review Instrument [Internet]. United States of America: K4Health; 2010 [cited 2018 May 24]. Available from: https://www.k4health.org/sites/default/files/RMC Program Review Instrument_0.pdf

5. Phillips JE. Effects of Change Valence and Informational Assessments on Organizational Readiness for Change T [Internet]. Walden University, Cape Town; 2017 [cited 2018 Sep 27]. Available from: https://scholarworks.waldenu.edu/dissertations/4016/

6. World Health Organization. WHO recommendations: Intrapartum care for a positive childbirth experience [Internet]. Geneva, Switzerland; 2018 [cited 2018 Aug 8]. 1–200 p. Available from: https://www.who.int/reproductivehealth/publications/intrapartum-care-guidelines/en/

7. Judge TA, Erez A, Bono J., Thoresen CJ. The Core Self-Evaluation Scale (CSES): Development of a Measure. Pers Psychol. 2003;56:303–31.

8. O'Neil M, Reimann S. Health Systems in Action: An eHandbook for Leaders and Managers. In: Managing Human Resources [Internet]. 2014th ed. Medford, MA, United States of America: Management Sciences for Health; 2010 [cited 2019 Jan 14]. Available from: http://www.msh.org/resources/health-systems-in-action-an-ehandbook-for-leaders-and-

For peer review only

managers

For peer review only

Additional file 2

1. Principal Component Analsysis Results for the scale assessing Health Provider's perception of women's rights during childbirth.

Test	Measure	Statistics
Kaiser-Meyer-Olkin (KMO)	Sampling adequacy	[a]0.620
Bartlett's	Sphericity	$\chi^2 = 229.126$
		df = 78
		$p < 0.001$*

Total Variance explained

Component	Eigenvalues	% of variance	Cumulative %
1	2.326	17.89	17.89
2	1.448	11.14	29.04

* Statistically significant [a] Sample is adequate (No of observations -203)

For peer review only

Principal component eignevectors for 4 of 13 components

Variable	Comp1	Comp2	Comp3	Comp4	Comp5
right_Know~r	0.3444	-0.0563	0.0809	0.4687	0.1214
right_priv~y	0.2609	-0.5047	0.0694	0.1062	0.1655
right_info~n	0.1528	-0.1522	0.1208	0.0868	0.6739
right_refu~t	0.1797	0.3353	-0.2941	0.3634	0.0657
right_rese~t	0.2260	0.4095	-0.3549	0.1507	0.2613
right_acce~s	0.2397	-0.2237	-0.3648	-0.3109	0.1185
right_cult~e	0.3100	-0.3255	0.0846	0.2425	-0.4273
right_nola~r	0.0559	0.2120	0.7022	-0.0018	0.2357
right_comp~p	0.3543	0.0971	0.3081	-0.0594	-0.2283
right_mobi~r	0.3228	-0.0195	-0.0627	-0.3274	0.1690
right_noba~n	0.1915	0.4536	0.1623	-0.2516	-0.0634
right_pain~f	0.4288	0.1441	-0.0478	0.0854	-0.3068
right_birt~n	0.3157	-0.0396	-0.0281	-0.5196	0.0064

Correlation matrix showing the total, mean scores and pca predicted scores (final rights) for health provider's perception and frequency of women's rights during childbirth.

	Total scores _perceived women's rights	Mean scores _perceived women's rights	No of items agreed to_ _perceived women's rights	Pca scores _perceived women's rights
Total scores _	1.000			
Mean scores	1.000	1.000		
No of items agreed	0.9157	0.9157	1.000	
Pca scores	0.9919	0.9919	0.9073	1.000

2. Principal Component Analsysis Results for the scale assessing Health Provider's awareness of the frequency of mistreatment f o women during childbith at their own health facilities

Test	Measure	Statistics
Kaiser-Meyer-Olkin (KMO)	Sampling adequacy	[a]0.720
Bartlett's	Sphericity	χ^2 = 357.784
		df = 66
		p < 0.001*

Total Variance explained

Component	Eigenvalues	% of variance	Cumulative %
1	2.783	23.19	23.19
2	1.440	12.00	35.19

* Statistically significant [a] Sample is adequate (No of observations -211)

Principal component eignevectors for 4 of 12 components

Variable	Comp1	Comp2	Comp3	Comp4
physical_a~e	0.1505	-0.1880	0.5230	0.3352
verbal_abuse	0.3260	-0.3530	0.3263	0.1119
lack_of_in~n	0.4237	-0.0759	-0.2696	0.2439
no_informe~t	0.2234	0.1038	-0.4436	0.4205
lack_of_pr~y	0.3758	0.2156	0.0596	-0.2068
lack_confi~y	0.4621	0.0288	-0.1338	-0.1383
discrimina~n	0.4057	-0.1312	-0.1291	-0.0910
no_birth_c~n	0.1262	0.3171	0.2598	0.2618
no_movt_in~r	0.0401	0.4763	0.3472	-0.1348
no_choice_~n	0.1061	0.5535	0.1156	0.3253
separation~y	0.1021	-0.3402	0.3182	0.0749
abandonmen~e	0.2883	0.0975	0.0988	-0.6087

Correlation matrix showing the total scores, mean scores and pca predicted scores (final mistreatment) for health provider's awareness of the frequency of women's mistreatment during childbirth at their facilities

	Total scores _perceived women's rights	Mean scores _perceived women's rights	No of items agreed to_ _perceived women's rights	Pca scores _perceived women's rights
Total scores _	1.000			
Mean scores	1.000	1.000		
No of items agreed	-0.7437	-0.7437	1.000	
Pca scores	0.8658	0.8658	-0.4638	1.000

For peer review only - http://bmjopen.bmj.com/site/about/guidelines.xhtml

3. Principal component analysis results for the scale assessing health provider's perception of resource availability for the implementation of RMC as recommended by the World Health Organisation

Test	Measure	Statistics
Kaiser-Meyer-Olkin (KMO)	Sampling adequacy	[a]0.640
Bartlett's	Sphericity	χ^2 = 557.535
		df = 153
		p < 0.001*

Total Variance explained

Component	Eigenvalues	% of variance	Cumulative %
1	2.968	16.49	16.49
2	2.242	12.46	28.95

* Statistically significant [a] Sample is adequate (No of observations -173)

Principal component eignevectors for 6 of 18 components

Variable	Comp1	Comp2	Comp3	Comp4	Comp5	Comp6
adequate_s~f	0.1937	-0.3240	0.2060	-0.0295	0.1330	-0.1381
mgt_sensit~d	0.1834	0.1923	-0.4623	-0.0580	0.1970	-0.0811
regular_rm~g	0.3397	-0.0619	-0.3633	-0.2583	-0.1123	-0.0819
written_gu~s	0.3728	-0.0247	0.0683	-0.3796	-0.1623	0.0967
informed_c~t	0.0625	0.5407	0.1832	-0.1032	0.0221	0.0626
rmc_educat~s	0.2679	0.0166	0.3492	-0.3203	-0.1146	0.1170
rooming_in	-0.0196	0.2111	-0.0005	0.0803	-0.0010	0.6030
clean_priv~e	0.2048	0.2019	-0.1084	0.3604	-0.2740	0.0965
clean_bath~s	0.2307	0.1783	0.0003	0.3818	-0.3967	0.0724
safe_water~e	0.0780	0.1868	0.1777	0.2380	0.0806	-0.6417
curtains_a~s	0.3270	0.1311	0.1233	0.1952	-0.1454	-0.2312
adequate_b~s	0.2094	-0.1672	0.2842	0.2932	0.4128	0.1370
space_woma~s	0.1107	-0.3020	0.3363	-0.0534	-0.3750	-0.0117
adequate_l~t	0.1908	-0.3234	-0.0153	0.3417	0.2592	0.2372
power_supp~r	0.3325	-0.0968	-0.0659	0.0334	-0.0543	0.1038
rmc_practi~w	0.2486	-0.2531	-0.4220	0.0271	0.0132	-0.0762
suggestion~x	0.2576	0.2220	0.1264	-0.2739	0.4053	-0.0240
redress_co~e	0.2430	0.2129	0.0257	0.1119	0.2920	0.0707

Correlation matrix showing the total scores, mean scores and principal component analysis predicted scores (final res) predicted scores for health provider's perception on the availability of resources needed to implement respectful maternity care as recommended by the World Health Organisation

	Total scores _resource availability	Mean scores _ resource availability	No of items agreed to_ _ resource availability	Pca scores _ resource availability
Total scores _	1.000			
Mean scores	1.000	1.000		
No of items agreed	0.9206	0.9206	1.000	
Pca scores	0.9576	0.9576	0.8657	1.000

For peer review only - http://bmjopen.bmj.com/site/about/guidelines.xhtml

STROBE Statement—Checklist of items that should be included in reports of ***cross-sectional studies***

Title: Organisational and individual readiness for change to respectful maternity care practice and associated factors in Ibadan, Nigeria: a cross-sectional study

	Item No	Recommendation		Page
**Title and abstract**	1	(*a*) Indicate the study's design with a commonly used term in the title or the abstract	✓	1
		(*b*) Provide in the abstract an informative and balanced summary of what was done and what was found	✓	2
**Introduction**				
Background/rationale	2	Explain the scientific background and rationale for the investigation being reported	✓	4
Objectives	3	State specific objectives, including any pre-specified hypotheses	✓	5
**Methods**				
Study design	4	Present key elements of study design early in the paper	✓	5
Setting	5	Describe the setting, locations, and relevant dates, including periods of recruitment, exposure, follow-up, and data collection	✓	5
Participants	6	(*a*) Give the eligibility criteria, and the sources and methods of selection of participants	✓	5-6
Variables	7	Clearly define all outcomes, exposures, predictors, potential confounders, and effect modifiers. Give diagnostic criteria, if applicable	✓	6-7
Data sources/ measurement	8*	For each variable of interest, give sources of data and details of methods of assessment (measurement). Describe comparability of assessment methods if there is more than one group	✓	*Additional file 2*
Bias	9	Describe any efforts to address potential sources of bias (Adjusted for clustering effect)	✓	7
Study size	10	Explain how the study size was arrived at	✓	5
Quantitative variables	11	Explain how quantitative variables were handled in the analyses. If applicable, describe which groupings were chosen and why	✓	7-8
Statistical methods	12	(*a*) Describe all statistical methods, including those used to control for confounding	✓	8
		(*b*) Describe any methods used to examine subgroups and interactions	✓	8
		(*c*) Explain how missing data were addressed	No missing data	
		(*d*) If applicable, describe analytical methods taking account of sampling strategy	✓	7
		(*e*) Describe any sensitivity analyses	Not applicable	
**Results**				
Participants	13*	(a) Report numbers of individuals at each stage of study—eg numbers potentially eligible, examined for eligibility, confirmed eligible, included in the study, completing follow-up, and analysed	✓	8-9

For peer review only - http://bmjopen.bmj.com/site/about/guidelines.xhtml

		(b) Give reasons for non-participation at each stage	Not applicable	
		(c) Consider use of a flow diagram	Not applicable	
Descriptive data	14*	(a) Give characteristics of study participants (eg demographic, clinical, social) and information on exposures and potential confounders	✓	8-9
		(b) Indicate number of participants with missing data for each variable of interest	Not applicable	
Outcome data	15*	Report numbers of outcome events or summary measures	✓	10
Main results	16	(*a*) Give unadjusted estimates and, if applicable, confounder-adjusted estimates and their precision (eg, 95% confidence interval). Make clear which confounders were adjusted for and why they were included	✓	11
		(*b*) Report category boundaries when continuous variables were categorized	✓	9 & 11
		(*c*) If relevant, consider translating estimates of relative risk into absolute risk for a meaningful time period		Not applicable
Other analyses	17	Report other analyses done—eg analyses of subgroups and interactions, and sensitivity analyses	Not applicable	
**Discussion**				
Key results	18	Summarise key results with reference to study objectives	✓	16
Limitations	19	Discuss limitations of the study, taking into account sources of potential bias or imprecision. Discuss both direction and magnitude of any potential bias	✓	16-17
Interpretation	20	Give a cautious overall interpretation of results considering objectives, limitations, multiplicity of analyses, results from similar studies, and other relevant evidence	✓	17-21
Generalisability	21	Discuss the generalisability (external validity) of the study results	✓	17
**Other information**				
Funding	22	Give the source of funding and the role of the funders for the present study and, if applicable, for the original study on which the present article is based	✓	22

*Give information separately for exposed and unexposed groups.

For peer review only

Organisational and individual readiness for change to respectful maternity care practice and associated factors in Ibadan, Nigeria: a cross-sectional survey

Journal:	*BMJ Open*
Manuscript ID	bmjopen-2022-065517.R1
Article Type:	Original research
Date Submitted by the Author:	16-Sep-2022
Complete List of Authors:	Esan, Oluwaseun; Obafemi Awolowo University College of Health Sciences, Department of Community Health, Faculty of Clinical Sciences; University of the Witwatersrand Faculty of Health Sciences, Centre for Health Policy, School of Public Health<br>Maswime, Salome; University of Cape Town Faculty of Health Sciences, Global Surgery Division, Department of Surgery<br>Blaauw, Duane; University of the Witwatersrand Faculty of Health Sciences, Centre for Health Policy, School of Public Health
**Primary Subject Heading**:	Public health
Secondary Subject Heading:	Health services research, Reproductive medicine
Keywords:	Change management < HEALTH SERVICES ADMINISTRATION & MANAGEMENT, Health policy < HEALTH SERVICES ADMINISTRATION & MANAGEMENT, PUBLIC HEALTH, REPRODUCTIVE MEDICINE, Quality in health care < HEALTH SERVICES ADMINISTRATION & MANAGEMENT, Organisation of health services < HEALTH SERVICES ADMINISTRATION & MANAGEMENT

I, the Submitting Author has the right to grant and does grant on behalf of all authors of the Work (as defined in the below author licence), an exclusive licence and/or a non-exclusive licence for contributions from authors who are: i) UK Crown employees; ii) where BMJ has agreed a CC-BY licence shall apply, and/or iii) in accordance with the terms applicable for US Federal Government officers or employees acting as part of their official duties; on a worldwide, perpetual, irrevocable, royalty-free basis to BMJ Publishing Group Ltd ("BMJ") its licensees and where the relevant Journal is co-owned by BMJ to the co-owners of the Journal, to publish the Work in this journal and any other BMJ products and to exploit all rights, as set out in our licence.

The Submitting Author accepts and understands that any supply made under these terms is made by BMJ to the Submitting Author unless you are acting as an employee on behalf of your employer or a postgraduate student of an affiliated institution which is paying any applicable article publishing charge ("APC") for Open Access articles. Where the Submitting Author wishes to make the Work available on an Open Access basis (and intends to pay the relevant APC), the terms of reuse of such Open Access shall be governed by a Creative Commons licence – details of these licences and which Creative Commons licence will apply to this Work are set out in our licence referred to above.

Other than as permitted in any relevant BMJ Author's Self Archiving Policies, I confirm this Work has not been accepted for publication elsewhere, is not being considered for publication elsewhere and does not duplicate material already published. I confirm all authors consent to publication of this Work and authorise the granting of this licence.

For peer review only

Organisational and individual readiness for change to respectful maternity care practice and associated factors in Ibadan, Nigeria: a cross-sectional survey

1. **Dr Oluwaseun Taiwo Esan**

Department of Community Health, Faculty of Health Sciences,

Obafemi Awolowo University/ Teaching Hospitals Complex, Ile-Ife, Osun State, Nigeria.

& Centre for Health Policy, School of Public Health, University of the Witwatersrand, Johannesburg, South Africa

Email: o.esan@oauife.edu.ng

ORCID: 0000-0002-2908-6034

2. **Professor Salome Maswime**

Global Surgery Division, Department of Surgery, Faculty of Health Sciences,

University of Cape Town, Cape Town. South Africa

ORCID: 0000-0003-4013-5164

E-mail: salome.maswime@uct.ac.za

3. **Dr Duane Blaauw**

Centre for Health Policy, School of Public Health,

Faculty of Health Sciences, University of the Witwatersrand, Johannesburg, South Africa.

ORCID: 0000-0002-0605-7134

E-mail: duane.blaauw@wits.ac.za

<u>Correspondence to:</u>

Dr Oluwaseun Taiwo Esan

o.esan@oauife.edu.ng

Word count: 4,577

Abstract

Objectives: This study assessed health providers' organisational and individual readiness for change to respectful maternity care (RMC) practice and their associated factors in Ibadan Metropolis, Nigeria.

Design: A cross-sectional survey using standardised structured instruments adapted from the literature.

Setting: Nine public health facilities in Ibadan Metropolis, Nigeria, December 1, 2019, to May 31, 2020.

Participants: 212 health providers selected via a two-stage cluster sampling.

Outcomes: Organisational readiness for change to RMC (ORC_{RMC}) and individual readiness for change to RMC (IRC_{RMC}) scales had a maximum score of 5. Multiple linear regression was used to identify factors influencing IRC_{RMC} and $ORC_{RMC.}$ We evaluated previously identified predictors of readiness for change (change valence, informational assessments on resource adequacy, core self-evaluation and job satisfaction) and proposed others (workplace characteristics, awareness of mistreatment during childbirth, perceptions of women's rights and resource availability to implement RMC). Data were adjusted for clustering and analysed using Stata 15.

Results: The providers' mean age was 44.0±9.9 with 15.4±9.9 years of work experience. They scored high on awareness of women's mistreatment (3.9±0.5) and women's perceived rights during childbirth (3.9±0.5). They had high ORC_{RMC} (4.1±0.9) and IRC_{RMC} (4.2±0.6), both weakly but positively correlated (*rho*=0.407, 95% CI: 0.288-0.514, p<0.001). Providers also had high change valence (4.5±0.8) but lower perceptions of resource availability (2.7±0.7) and adequacy for implementation (3.3±0.7). Higher provider change valence and informational assessments were associated with significantly increased IRC_{RMC} (β 0.40 [95% CI: 0.11-0.70,

For peer review only
For peer review only - http://bmjopen.bmj.com/site/about/guidelines.xhtml

p=0.015] and β 0.07 [95% CI: 0.01-0.13, p=0.032], respectively), and also with significantly increased ORC_{RMC} (β 0.47 [95% CI: 0.21-0.74, p=0.004] and β 0.43 [95% CI: 0.22-0.63, p=0.002], respectively). Longer years of work experience (β 0.08, 95% CI:0.01-0.2, p=0.024), providers' monthly income (β 0.08, 95% CI:0.02-0.15, p=0.021) and the health facility of practice were associated with significantly increased ORC_{RMC}.

Conclusion: The health providers studied valued a change to RMC and believed that both they and their facilities were ready for the change to RMC practice.

Keywords: Organisational readiness, individual readiness, readiness for implementing change, respectful maternity care, pre-implementation research, change commitment and efficacy

Strengths and limitations of this study

- The study was conducted in the pre-implementation phase before the integration of respectful maternity care practice into routine childbirth care in the study location.
- Organisational and individual readiness for change theories were tested quantitatively using very brief standardised assessment scales (12-item and 6-item) among health providers, with zero non-response rate recorded.
- All categories of maternal health care providers were interviewed, which may facilitate stakeholder engagement during the implementation process.
- The study was limited in its geographical scope as it was conducted in Ibadan Metropolis, one metropolitan area in a South-Western Nigerian state.

For peer review only

For peer review only - http://bmjopen.bmj.com/site/about/guidelines.xhtml

- The study was further limited in its scope as tertiary health facilities were not studied because there was only one tertiary health facility serving the populations in the study location.

Introduction

The utilisation of maternal care services, especially during childbirth, is low in Nigeria. The proportion of women whose delivery utilised a skilled birth attendant in 2018 was 43.3%.[1] One of the reasons explaining this is women's mistreatment during birth.[2,3] Negative health worker attitudes have been expressed as mistreatment, particularly during childbirth, and this has been reported frequently, both globally and in Nigeria specifically. Ogunlaja *et al*[4] found a 93.2% reported prevalence of mistreatment in previous deliveries among 438 antenatal clients in Ogbomoso, Oyo state. The Nigerian prevalence of women's mistreatment during childbirth was reported as ranging from 11% to 71% according to a systematic review of 14 studies between 2004 and 2015.[5] Respectful maternity care practices have been prioritised as a means to improve patient-provider interactions and the quality of maternal care experienced.

Respectful maternity care has been defined as "*care organised for and provided to all women in a manner that maintains their dignity, privacy and confidentiality, ensures freedom from harm and mistreatment, and enables informed choice and continuous support during childbirth*" (page 3).[6] It is a human rights approach to maternity care[7] and is recommended as the standard for all women.[8] Several RMC-promoting interventions have been implemented and have shown promising results.[9] For these results to be enduring and sustainable, the health providers will need to embrace and support the interventions. This can be achieved if they are ready for the change to an RMC practice.

Readiness for change measures the extent to which people or organisations are inclined to adopt a change that alters the "status quo".[10] It addresses the psychological and behavioural forms of readiness for change, that is the state of being willing and able to change.[11,12] Some authors also

For peer review only - http://bmjopen.bmj.com/site/about/guidelines.xhtml

describe it as having a structural component that addresses the presence or absence of financial, material, and human resources needed for a change, such as to RMC practice.[12] Readiness for change is a multilevel construct measured at individual and organisational levels. Organisational readiness for change is a multifaceted concept that consists of employees' change commitment (collective resolve) and change efficacy (perceived shared ability) to implement the change.[11] Individual readiness for change is an employee's confidence to manage the change or willingness to accept new roles and adopt new practices.[13] Readiness for change is different from preparedness as the latter addresses the set activities to implement the change[14] while readiness measures being both prepared and motivated to implement the change.

Readiness for change is a key determinant of implementation success.[15,16] The readiness for change theories have been applied in both health and non-health organisations, however, there are no previous studies on their application to RMC-promoting interventions. Readiness for change to RMC among community, facility and policy stakeholders was mentioned as being responsible for the positive results of an RMC project in Kenya.[17] However, readiness for change was not measured directly in that study.[17] Many RMC-promoting interventions have been conducted without prior assessment of the individual employee or organisational readiness for change.[18,19] If readiness is assessed and found wanting, efforts can be directed at improving it. If otherwise, this suggests the providers' willingness to accept the change irrespective of the work task demands brought by it.

The proposed theory of change for this study is that a high organisational and individual readiness for change would lead to the adoption and institutionalisation of RMC practice which should result in long-term outcomes such as increased health facility delivery. Adoption is the temporary altering of attitudes and behaviours to meet the change expectations. Institutionalisation occurs when the change becomes part of the organisational processes.[10] This is assuming all limiting barriers and contextual factors have been identified and addressed. The

For peer review only - http://bmjopen.bmj.com/site/about/guidelines.xhtml

barriers and contextual factors have been explored but the data are yet to be published. This study assessed health providers' organisational and individual readiness for change to RMC practice and their associated factors in Ibadan Metropolis, Nigeria.

Methods

Design, setting, and participants

This was a cross-sectional survey conducted from December 1, 2019, to May 31, 2020, in Ibadan Metropolis, Oyo state, Nigeria. Ibadan (the third largest city in Nigeria and the seventh in Africa) was selected, being a more cosmopolitan city. This study was conducted among public health care providers from the five Local Government Areas (LGA) in Ibadan Metropolis. There were 6 public secondary and 26 functional public primary health facilities in the five LGAs with a minimum of 12 deliveries per year at the time of conducting the study. Maternity care services, including delivery services, are offered in all facilities, with more specialised care at secondary health facilities. Doctors and nurses attend deliveries at both primary and secondary health facilities, while Community Health Officers (CHOs), Community Health Extension Workers (CHEWs) and Health Auxiliaries (HA) only attend deliveries at primary health facilities in the study state.

A two-stage cluster sampling technique was used to select the health facilities and providers in the study LGAs. One primary and one secondary health facility were selected in each LGA using simple random sampling, except in one LGA without a secondary health facility. This gave a total of nine health facilities studied (5 primary and 4 secondary health facilities). There were a total of 244 health providers (as the study population or sampling frame) who could attend deliveries in the study facilities (176 in the 4 secondary facilities and 68 in the 5 primary facilities).

A sample size of 210 health providers was calculated using the one-sample mean test[20] in Stata. This represented 86% of the study population. The parameters used were a change commitment

For peer review only - http://bmjopen.bmj.com/site/about/guidelines.xhtml

147 mean of 3.64±0.61 standard deviation (SD), based on a similar study in Switzerland, as a proxy
148 for organisational readiness.[21] The required precision was ±5% about the reference mean, with
149 90% power, and a design effect of 2[22] for the cluster sampling. The number of health providers
150 interviewed at each facility was allocated proportionately to the generated total number of
151 health providers per professional type at each health facility within the LGAs.

152 All the available and consenting health providers at each health facility were interviewed until
153 the required numbers of each professional type for each facility were reached. As the health
154 workers work in shifts (and thus may not have been working at the time of initial approach), if
155 the number to be interviewed was yet to be reached after interviewing all the available and
156 consenting health workers on the morning and afternoon shifts, the data collectors repeatedly
157 visited the facilities to recruit workers on shifts on later dates. We did not document the number
158 that did not consent, but the majority of those who were approached consented and were
159 interviewed.

160 Ethical approvals were obtained from the Human Research Ethics Committees of the University
161 of the Witwatersrand, Johannesburg (clearance Number M190658), and the Oyo State Ministry
162 of Health (Ref. Number AD/13/479/1386).

163 **Data collection**

164 Data collection was done using a 112-item tool with 9 sections developed in REDCap and
165 conducted within the health facility premises.[23] Two trained research assistants administered
166 the questionnaire. The tools were pre-tested among 12 health providers from one public
167 secondary health facility in Ibadan North-West LGA and one public primary health facility in
168 Ibadan North LGA, after a two-day training. Findings from the pre-test were used to improve
169 the data collection instruments. The first part of the instrument assessed health providers'
170 perceptions of women's rights during childbirth, their awareness of women's mistreatment

For peer review only

during childbirth in their health facilities, and their awareness of the RMC concept. A one-page brief on 'RMC during childbirth' was read to each respondent (see Additional file 1). The subsequent sections of the questionnaire evaluated providers' perceptions of individual and organisational readiness for change to RMC practice during childbirth, and possible associated factors, using standardised tools. (See Additional file 2 for the survey instrument)

The respondents' perceived organisational readiness and individual readiness for change to RMC practice were the outcome variables. Organisational readiness for change to RMC (ORC_{RMC}) was assessed using a standardised 12-item Organisational Readiness for Implementing Change (ORIC) tool[24] with 5 items measuring their change commitment and 7-items assessing their change efficacy, both on a 5-point Likert agreement scale.[24] The questions assessing organisational readiness were framed as, *"The health workers in this health facility are…"* Organisational readiness was determined as the mean score of the 12 items on the scale with a maximum score of 5. Individual readiness for change to RMC (IRC_{RMC}) was measured using a 6-item tool on a 5-point Likert agreement scale by Vakola et al.[13] Questions were framed as *"I am willing to…"*. IRC_{RMC} was determined as the mean score of the 6-item scale, also with a maximum score of 5. When reported as percentages, the mean scores were standardised and converted to it using the formula (Mean-1)/4*100.

For the predictors, we included factors well described in the implementation science literature and used standardised tools. The previously defined predictors of ORC_{RMC} by Weiner[11] include employee change valence (how much they value the change) and informational assessments (perceived adequacy of the resources available to implement the change). Previously defined predictors of IRC_{RMC} by Vakola et al[13] include employee job satisfaction and core self-evaluation (which assesses their self-esteem, locus of control, emotional stability and generalised self-efficacy).[25] We evaluated all of these predictors on both IRC_{RMC} and ORC_{RMC}.

For peer review only - http://bmjopen.bmj.com/site/about/guidelines.xhtml

In addition, we also proposed that individual provider characteristics such as being younger, having more years of experience, and having higher monthly income could positively influence IRC_{RMC} and ORC_{RMC}. We suggested that health providers' perceptions about women's rights during childbirth, their perceived availability and adequacy of resources for RMC implementation and differences in their workplace contexts might influence both IRC_{RMC} and $ORC_{RMC.}$ Additional file 3 summarises the study's analytical framework and Additional file 4 gives the list of standardised tools used to assess the analytical constructs, together with their reliability statistics in our study. The highest Cronbach's alpha was 0.949 for the organisational readiness for change tool, while the lowest was 0.575 for the tool assessing providers' perception of women's rights.

Data analysis

Data collected were uploaded to the University of the Witwatersrand data management system via REDCap. Only the first author had access to download and save on a passworded computer, then shared with the co-authors. The dataset has been shared with the Figshare repository.[26]

Data analysis was done using the Stata version 15 software. We adjusted for weighting and facility-level clustering in all analyses using the Stata 'svy' commands. The mean scores of the outcome and predictor variables were determined. Higher mean scores indicate higher IRC_{RMC} and ORC_{RMC}. Pearson's correlation was used to evaluate the relationship between IRC_{RMC} and $ORC_{RMC,}$ change efficacy and change commitment, and resource availability and adequacy.

Principal component analysis (PCA) was used to construct separate composite indices for the study-specific tools assessing providers' perceptions of women's rights, their awareness of mistreatment in their facilities, and the availability of resources for RMC practice. Details are provided in Additional file 5. The first components explained 17.9%, 23.2% and 16.5% of the

For peer review only - http://bmjopen.bmj.com/site/about/guidelines.xhtml

variance for each of these scales respectively. These PCA scores were then used in the bivariate and multiple regression analysis as potential predictors.

Simple linear regression was done to assess the bivariate relationship between the two numerical outcomes and the predictor variables. Predictors with a p-value ≤0.2 were included in the final multiple regression models for each outcome variable. All predictors were added simultaneously. Multicollinearity analysis was conducted after the regressions. Predictor variables with a high variance inflation factor (>10.0) were excluded from the model.

Patient and public involvement

A prior qualitative study of pregnant women's perceptions of RMC[27] informed this study, the study location, and many of the variables assessed. The women described their experience of childbirth care and queried the readiness of the health providers to provide such care.

Results

Socio-demographic profile

212 health providers finally completed the survey, slightly above the required sample size of 210 (with the slight oversampling due to separate data collection by two data collectors). The breakdown by their professional group as shown in Table 1. Their overall mean age was 44.0. The doctors were the youngest with a mean age (in years) of 38.9 while the health auxiliaries were the oldest with a mean age of 49.3. Overall, the respondents had an average of >15 years post-training work experience, which included an average of about 6 years working at the study facility.

Table 1: Providers' socio-demographic profile by provider type

Variables	Doctor n=38	Nurse n=128	CHEW/CHO n=29	Auxiliary n=18	Total n=212
**Age**					
Mean ± SD	38.9 ±9.9	44.6 ± 9.4	44.5 ± 9.7	49.3 ± 10.5	44.0 ± 9.9
Median (IQR)	40 (31- 46)	44 (39 - 52)	46 (39 - 50)	52 (40 - 56)	44 (38 - 52)
**Sex**					
Male	20 (52.3)	0 (0.0)	2 (8.5)	0 (0.0)	22 (10.4)

For peer review only - http://bmjopen.bmj.com/site/about/guidelines.xhtml

Female	18 (47.7)	127 (100.0)	26 (91.5)	18 (100.0)	190 (89.6)
**Type of health facility**					
Primary health facility	3 (8.7)	9 (7.1)	29 (100.0)	18 (100.0)	59 (27.9)
Secondary health facility	34 (91.3)	119 (92.9)	0 (0.00	0 (0.0)	153 (72.1)
**LGA**					
Ibadan North	23 (61.9)	61 (47.8)	3 (12.0)	2 (9.7)	89 (42.2)
Ibadan North East	5 (14.6)	14 (11.2)	5 (16.2)	2 (8.8)	26 12.3)
Ibadan North West	6 (15.6)	20 (15.9)	12 (41.6)	3 (15.0)	41 (19.3)
Ibadan South East	1 (2.9)	1 (0.8)	4 (14.8)	7 (41.8)	14 (6.6)
Ibadan South West	2 (5.1)	31 (24.3)	4 (15.3)	4 (24.7)	42 (19.7)
**Study Health facilities**					
Facility 1	1 (2.9)	2 (1.4)	3 (12.0)	2 (9.7)	8 (8.7)
Facility 2	23 (59.6)	59 (46.4)	0 (0.0)	0 (0.0)	82 (38.5)
Facility 3	0 (0.0)	2 (1.2)	5 (16.2)	2 (8.8)	9 (3.7)
Facility 4	5 (44.6)	13 (10.0)	0 (0.0)	0 (0.0)	18 (8.6)
Facility 5	1 (3.6)	1 (1.1)	12 (41.6)	3 (15.0)	17 (8.2)
Facility 6	5 (12.0)	19 (14.8)	0 (0.0)	0 (0.0)	23 (11.1)
Facility 7	0 (0.0)	3 (2.6)	5 (15.3)	4 (24.7)	12 (5.7)
Facility 8	2 (5.1)	28 (21.7)	0 (0.0)	0 (0.0)	30 (13.9)
Facility 9	1 (2.3)	1 (1.0)	4 (14.8)	7 (41.8)	13 (6.6)
**Years of experience**					
Mean ± SD	10.4 ± 7.7	18.2 ± 9.9	10.5 ± 7.5	13.6 ± 11.0	15.4 ± 9.9
Median (IQR)	10 (3 - 14)	18 (11 - 25)	8 (4 - 17)	9 (6 - 190)	14 (7 - 23)
**Years working in study facility**					
Mean ± SD	3.2 ± 3.5	8.2 ± 6.0	2.7 ± 1.9	3.7 ± 2.0	6.0 ± 5.6
Median (IQR)	2 (0.5 -5)	7 (4 - 11)	3 (1 - 4)	4 (3 - 5)	5 (2 - 10)
**Income (in USD)**					
Median (IQR)	658 (526 - 921)	500 (289 – 553)	270 (132 -395)	99 (26 – 191)	463(263 – 605)

Note: IQR – Interquartile range

RMC- women's rights and mistreatment and needed resources

Overall, 35.9% of the providers had heard of RMC. This consisted mainly of the doctors (60%) and the least (19.1%) being the health auxiliaries. Nonetheless, after RMC had been explained to them, 70% of all the providers agreed that RMC could be implemented in their facilities.

As shown in Figure 1, 72.9% of the health providers stated that women delivering in their facility were always denied a birth companion, 63.9% were aware of women not being allowed to decide their birth position, and 36.7% had witnessed restrictions on mobility during labour. Correspondingly, only 19.9% of health providers believed that women should always have the right to decide their birth position, 38.7% agreed that women could be mobile during labour, and 50.7% supported women having a birth companion (Figure 1). Only 20.4% accepted that women should always have unrestricted access to their hospital records.

Figure 2 indicates providers' perceptions of the availability of essential 18 WHO-recommended resources for implementing RMC. The least available resource was RMC educational materials

For peer review only

For peer review only - http://bmjopen.bmj.com/site/about/guidelines.xhtml

(7.7%), followed by guidelines (8.2%). Approximately 10-15% of the providers agreed to the availability of private spaces to support birth companions, in-service training on RMC, suggestion boxes, and adequately trained staff on RMC. However, 63.0% of them agreed to have curtains and screens for privacy during childbirth.

The mean scores for all the study scales are shown in Table 2. The health providers were well aware of the mistreatment of women during childbirth in their health facilities across the 12 items with a high mean score of 3.9±0.5 out of a maximum of 5. However, the mean score of 3.9±0.5 out of 5 also indicates high acceptance of the rights they believe women should always be granted during childbirth.

Individual and organisational readiness for change to RMC practice

In assessing organisational readiness for change to RMC (ORC_{RMC}), the health providers scored high on their commitment to the change and their change efficacy, which is their perceived ability to implement the change (Table 2). These two constructs were strongly positively correlated (rho: 0.830, 95% CI: 0.783-0.868, p<0.001). Combined, this gave a high mean organisational readiness for change (ORC_{RMC}) score of 4.01±0.9, which is 75.3% of the maximum obtainable mean score of 5. The health providers had even higher individual readiness for change to RMC (IRC_{RMC}), with a mean score of 4.23±0.6, 80.8% of the maximum. Organisational readiness was only moderately but significantly correlated with individual readiness for change to RMC (rho: 0.407, 95% CI: 0.29-0.51, p<0.001).

Table 2: Average provider perceptions for different study scales (n=212)

Analytical Category	Scale	Mean ± SD	95% CI
**Outcomes**	Change commitment	4.05 ± 1.0	3.8– 4.3
	Change efficacy	3.96 ± 0.9	3.6 – 4.3
	Organisational readiness for change (ORC_{RMC})	4.01 ± 0.9	3.7 – 4.3
	Individual readiness for change (IRC_{RMC})	4.23 ± 0.6	4.1 – 4.4
**Predictors**	Awareness of mistreatment during childbirth in their facilities	3.90 ± 0.5	3.7 – 4.1
	Women's rights during childbirth	3.85 ± 0.5	3.8 - 4.0
	Change valence	4.46 ± 0.8	4.3 – 4.6
	Informational assessments	3.30 ± 0.7	3.1 - 3.4
	Availability of resources to implement RMC in their facilities	2.70 ± 0.6	2.5 – 2.9

For peer review only - http://bmjopen.bmj.com/site/about/guidelines.xhtml

Core self-evaluation	4.34 ± 0.5	4.3 – 4.4
Job satisfaction	3.70 ± 0.6	3.6 – 3.8

Change valence and informational assessments

The health providers scored high on how much they value the change to RMC, with a mean of 4.46±0.8 out of 5 (Table 2). They, however, scored lower in their informational assessments ((3.30±0.7), which describes their perceptions on the adequacy of the available resources to implement the change to RMC practice in their facilities. The providers' mean score for the availability of the WHO-recommended resources to implement RMC was even lower (2.70±0.6), 42.5% of the maximum. There was a mild but significant positive relationship between their perceived availability and adequacy of the resources needed to implement RMC in their facilities, (rho: 0.263, 95% CI: 0.133-0.384, p=0.0001). Notwithstanding these perceived deficiencies, the health providers indicated relatively high levels of job satisfaction and core self-evaluation- that is, they had high self-esteem, locus of control, emotional stability and generalised self-efficacy (Table 2).

Factors associated with individual readiness for change (IRC_{RMC}) and organisational readiness for change (ORC_{RMC}) to RMC practice

Table 3 shows the bivariate and multiple regression analysis for IRC_{RMC}, while Table 4 shows the analysis for $ORC_{RMC.}$ The health providers' change valence and informational assessments were significantly associated with IRC_{RMC} in the multiple regression analysis, increasing IRC_{RMC} scores by (β: 0.40, 95% CI: 0.11–0.70, p=0.015) and (β: 0.07, 95% CI: 0.01–0.13, p=0.032) respectively. Doctors and nurses had significantly higher IRC_{RMC} than health assistants, in the bivariate analysis but this was no longer significant after adjusting for other covariates.

IRC_{RMC} varied significantly between health providers from different health facilities in the bivariate analysis but this was no longer the case in the multiple regression analysis. None of

For peer review only - http://bmjopen.bmj.com/site/about/guidelines.xhtml

the known predictors of individual readiness for change (providers' job satisfaction and core self-evaluation), nor the newly proposed ones (perceived rights of women, years of experience, income), was significantly associated with IRC_{RMC}.

Table 3: Analysis of factors associated with health providers' IRC_{RMC}

Covariates	Simple linear regression			Multiple linear regression		
	Crude Coeff.	95% CI	p-value	Adjusted Coeff.	95% CI	p-value
Health providers' age	-0.003	-0.01 - 0.007	0.497			
**Sex**						
Female	Ref	-	-	Ref	-	-
Male	-0.21	-0.02 – 0.44	0.065	-0.14	-0.84 – 1.13	0.743
**Study Local Government Area**						
Ibadan North	Ref	-	-			
Ibadan North East	0.02	-0.54 – 0.58	0.946			
Ibadan North West	-0.22	-0.47 – 0.03	0.078			
Ibadan South East	-0.45	-052 – 0.37	**<0.001**			
Ibadan South West	-0.09	-0.32 – 0.14	0.383			
**Health facility**						
Facility 1	0.19	0.10 – 0.19	<0.001	0.35	-0.01 – 0.70	0.053
Facility 2	Ref	-	-	Ref	-	-
Facility 3	-0.53	-0.53- -0.53	<0.001	-0.06	-0.39 – 0.27	0.675
Facility 4	0.29	0.29 – 0.29-	<0.001	0.07	-0.05 – 0.18	0.218
Facility 5	-0.37	0.37 - -0.37	<0.001	-0.23	-0.51 – 0.04	0.087
Facility 6	-0.06	-0.06 - -0.06	<0.001	0.04	-0.07 – 0.14	0.423
Facility 7	0.12	0.12 – 0.12	<0.001	0.22	-0.13 - 0.54	0.175
Facility 8	-0.10	-0.10 – -0.10	<0.001	0.0002	-0.09 – 0.09	0.970
Facility 9	-0.42	-0.42 - -0.42	<0.001	0.03	-0.30 – 0.35	0.844
**Providers' type of health facility**						
Primary health facility	Ref	-	-			
Secondary health facility	0.23	0.56 – 0.09	0.135			
**Professional cadre**						
Doctor	0.43	0.04 – 0.83	**0.036**	-0.13	-0.46 – 0.19	0.360
Nurse	0.37	0.05 – 0.70	**0.030**	Ref	-	-
CHEW/ CHO	0.08	-0.06 – 0.21	0.233	-0.19	-0.63 – 0.24	0.329
Health Assistant/ Aide	Ref	-	-	-0.16	-0.50 – 0.18	0.296
Monthly income (in USD/ 1000)	-2.36	-0.06 – 5.28	0.097	0.05	-0.03 - 0.13	0.187
Years of professional experience	0.01	0.004 – 0.03	0.106	0.004	-0.02 – 0.03	0.644
Years of experience in the health facility	0.004	-0.02 – 0.031	0.727			
Awareness of the mistreatment of women	0.01	-0.04 – 0.05	0.712			
Perceived women's rights during childbirth	0.04	-0.05 – 0.11	0.357			
**Ever heard of RMC (n=170)**						
Yes	-0.02	-0.33 – 0.30	0.883			
No	Ref	-	-			
**Perception of RMC being implementable**						
Agreed	0.10	-0.34 – 0.53	0.620			
Indifferent	Ref	-	-			
Disagreed	0.06	-0.46 – 0.58	0.794			
Change valence (value for RMC practice)	0.45	0.19– 0.71	**0.005**	0.40	0.11 – 0.70	**0.015**
RMC Informational assessment	0.07	0.15 - 0.42	**0.001**	0.07	0.01 – 0.13	**0.032**
Provider perceptions on available resources	0.03	-0.02 – 0.09	0.182			
Provider job satisfaction	0.010	-0.03 – 0.22	0.105	0.004	-0.13 – 0.14	0.953
Provider core self-evaluation	0.25	0.01 – 0.50	0.055	0.09	-0.22– 0.39	0.513
*Male # Doctor				0.15	-1.01 – 1.31	0.765

For peer review only - http://bmjopen.bmj.com/site/about/guidelines.xhtml

Constant	1.76	1.37 – 2..14	**<0.001**

n=212; R^2= 0.4363; p<0.001

Note: Ref: means the reference category; 95% CI: 95% Confidence Interval; Predictors with p-value ≤0.2 from the simple linear regression analysis were included in the multiple regression model; The Mean variance inflation factor vif for the multiple regression model is =2.33, significant p-values in bold. Male # Doctor- Interaction between gender and profession

Change valence and informational assessments were also significantly associated with ORC_{RMC} (Table 4). A unit increase in the health providers' change valence and informational assessments increased their perceived ORC_{RMC} by (β: 0.47, 95% CI: 0.21–0.74, p=0.004) and (β: 0.43, 95% CI: 0.22-0.63, p=0.002) units respectively, after adjusting for other covariates. Also, each additional 10 years of work experience significantly increased ORC_{RMC} by (β: 0.08, 95% CI: 0.01–0.2, p=0.024) and each $1000 increase in providers' monthly income increased their perceived ORC_{RMC} by (β: 0.08, 95% CI:0.02–0.15, p=0.021). There were significant varied associations (positively or negatively) between the health providers' facility of practice and their ORC_{RMC} in relation to the reference facility. The only exception was for Facility 4, a secondary health facility in one of the LGAs.

Table 4: Analysis of factors associated with health providers' ORC_{RMC}

Covariates	Simple linear regression			Multiple regression		
	Crude Coeff.	95% CI	p-value	Adjusted Coeff.	95% CI	p-value
Health providers' age	-0.01	-0.02 – 0.02	0.916			
**Sex**						
Female	Ref	-	-	Ref	-	-
Male	-0.21	-0.09 – 0.50	0.146	0.15	-0.11 – 0.41	0.213
**Study Local Government Area**						
Ibadan North	Ref	-	-			
Ibadan North East	0.34	-0.26 – 0.94	0.226			
Ibadan North West	-0.22	-0.42 – 0.02	**0.034**			
Ibadan South East	-0.46	-0.59 – 0.33	**<0.001**			
Ibadan South West	-0.27	-1.21 – 0.66	0.510			
**Health facility in LGA**						
Facility 1	0.43	0.43 – 0.43	**<0.001**	0.38	0.30 – 0.46	**<0.001**
Facility 2	Ref	-	-	-	-	-
Facility 3	-0.23	-0.23 - -0.23	**<0.001**	0.24	0.12 – 0.35	**0.002**
Facility 4	0.65	0.65 – 0.65	**<0.001**	0.16	-0.03 – 0.35	0.087
Facility 5	-0.09	-0.09 - -0.09	**<0.001**	-0.29	-0.40 - -0.19	**<0.001**
Facility 6	-0.24	-0.24 - -0.24	**<0.001**	-0.11	-0.16 - -0.07	**0.001**
Facility 7	0.63	0.63 – 0.63	**<0.001**	0.56	0.54 – 0.57	**<0.001**
Facility 8	-0.54	-0.54 - -0.54	**<0.001**	-0.41	-0.47- -0.36	**<0.001**
Facility 9	-0.41	-0.41 - -0.41	**<0.001**	0.11	0.02 – 0.20	**0.024**

For peer review only

For peer review only - http://bmjopen.bmj.com/site/about/guidelines.xhtml

**Providers' type of health facility**						
Primary health facility	Ref	-	-			
Secondary health facility	0.09	-0.68– 0.50	0.717			
**Professional cadre**						
Doctor	0.31	-0.50 – 1.12	0.391			
Nurse	-0.07	-0.88 – 0.75	0.857			
CHEW/ CHO	0.09	-0.38 – 0.56	0.667			
Health Assistant/ Aide	Ref	-	-			
Monthly income (in USD/ 1000)	0.26	-0..05- 0.56	0.083	0.08	0.02– 0.15	**0.021**
Years of professional experience /10 years	0.05	0.02 – 0.3	**0.034**	0.08	0.01 – 0.2	**0.024**
Years of experience in the health facility	-0.004	-0.03 – 0.02	0.678			
Awareness of the mistreatment of women	0.02	-0.06 – 0.09	0.650			
Perceived women's rights during childbirth	0.02	-0.12 – 0.16	0.767			
**Ever heard of RMC (n=170)**						
Yes	0.10	-0.27 – 0.46	0.553			
No	Ref	-	-			
**Perceptions of RMC being implementable**						
Agreed	0.60	-0.02 – 1.23	0.056	0.19	-0.08– 0.45	0.148
Indifferent	Ref	-	-	Ref	-	-
Disagreed	-0.09	-0.76 – 0.58	0.765	-0.12	-0.60 – 0.36	0.570
Change valence (value for RMC practice)	0.74	0.47 – 1.01	**<0.001**	0.47	0.21 – 0.74	**0.004**
RMC Informational assessment	0.72	0.40 – 1.05	**0.001**	0.43	0.22 – 0.63	**0.002**
Provider perceptions on available resources	-0.002	-0.25 – 0.25	0.984			
Provider job satisfaction	0.23	-0.08 – 0.55	0.125	0.05	-0.10 – 0.20	0.477
Provider core self-evaluation	0.15	-0.38 – 0.68	0.521			
Constant				0.06	-1.28 – 1.41	0.915

n=212; R^2= 0.6016; p<0.001

Note: Ref: means the reference category; CI: 95% Confidence Interval; Predictors with a p-value ≤0.2 from the bivariate analysis (simple linear regression) were included in the multiple regression model; The Mean variance inflation factor vif for the multiple regression model is =1.55, Significant p-values in bold.

Discussion

This is the first study to explore individual and organisational readiness for change to RMC practice, and the associated predictors. The health providers had a high level of awareness of the mistreatment of women but also a high general acceptance of women's rights during childbirth. However, there were some rights, such as being allowed a birth companion, that only a few providers regarded as essential, and these were then seldom practised. Nonetheless, the health providers scored high in their perceived IRC_{RMC} and ORC_{RMC}. IRC_{RMC} and ORC_{RMC} were only moderately correlated in this analysis. Higher change valence and informational assessment of the adequacy of resources increased not only ORC, as has been found previously,[24,28] but also IRC_{RMC}. Job satisfaction and the providers' core self-evaluation, which have been shown to influence IRC,[13,29] had no statistically significant effect on IRC_{RMC} in this study. The provider's years of work experience, their monthly income (individual

For peer review only

For peer review only - http://bmjopen.bmj.com/site/about/guidelines.xhtml

characteristics) and their health facility of practice (a workplace characteristic) significantly influenced ORC_{RMC}.

This study has provided an understanding of the state of readiness for change to RMC practice, eliminating it as a possible implementation problem for RMC practice in the study setting. We have established that IRC_{RMC} and ORC_{RMC} have a positive influence on each other. This study has also further confirmed the critical role of change valence and informational assessments in increasing both organisational and individual readiness for change to RMC practice. These findings have programmatic and policy implications for the designing of RMC implementation programs. The effect of employees' perceived value for newly introduced programs may also be evaluated on the program intervention and implementation outcomes.

The brevity of the organisational readiness for implementing change (ORIC) tool used to assess ORC_{RMC} among the health care providers studied was also beneficial. This is in contrast to other instruments assessing organisational readiness for change with a much higher number of constructs and variables.[12,30] The ORIC tool is a standardised instrument that has been validated among health worker populations in Western countries[24,31,32] and only in South Africa[33], with a similar population as found in our study. All categories of health providers involved in maternal care across cadres within the primary and secondary health facilities were studied. This may facilitate stakeholder engagement during the RMC implementation process and possible early adoption of the change.

The study findings however failed to establish a significant relationship between the providers' readiness for a change to RMC and their perceptions of women's rights during childbirth. Respectful maternity care is premised on the fundamental human rights of women to receive dignified care.[34] It would have been expected that provider perceptions of women's rights would be positively associated with their readiness for change. The relationship was in the

For peer review only - http://bmjopen.bmj.com/site/about/guidelines.xhtml

correct direction but not statistically significant. The provider's low perceptions of resource availability to implement RMC did also not significantly reduce their IRC_{RMC} and ORC_{RMC}.

This study had some limitations. It was a relatively small study and its geographical extent was limited to one Metro in Nigeria which may not be representative of similar facilities and providers in other regions of Nigeria. Tertiary health facilities were not included because there was only one tertiary health facility serving populations across the five LGAs studied. Social desirability bias may have influenced some of the providers' responses positively to the availability of resources and their perception of women's rights during childbirth. To mitigate this, the data collectors stressed the academic purpose of the research to the providers when obtaining informed consent. Limited awareness of RMC, as found in this study, may affect an accurate assessment of readiness for change. We attempted to address this by educating the providers on RMC concepts before assessing their readiness for change to RMC practice.

Health providers cannot truly be ready to implement RMC if they do not support certain women's rights during childbirth. This would result in persistent mistreatment and may prevent a positive change to RMC practice. The most common forms of mistreatment to women during childbirth in the study health facilities were being denied birth companions, not being allowed to decide on a birth position, and being denied mobility in labour. All three forms of mistreatment were also reported by Tanzanian women in a qualitative study of the perspectives of mothers and fathers on mistreatment during childbirth.[35] Several other studies have reported these forms of mistreatment experienced by women during childbirth.[36–39] According to the WHO,[40,41] having a birth companion during labour provides emotional support, reduces labour pain and strengthens the woman's capability to deliver. The WHO has also recommended that women are supported to deliver in their preferred birth position because alternative birth positions, such as standing to deliver, are safe and may result in shorter labour from better foetal alignment.[38,40] It has also been reported that mobility during the first stage of labour is safe.[38]

For peer review only - http://bmjopen.bmj.com/site/about/guidelines.xhtml

Denying women autonomy, or not respecting women's choices during childbirth without a justifiable medical reason, constitutes mistreatment that negatively affects their overall childbirth experience.[42]

The health providers perceived that women should always have the right to full information about their care and to receive their care in privacy. Unfortunately, many may not practice it for several reasons, including unconscious behaviour, an abusive work culture, and perceived excessive workload among others.[43] About 33% of maternity care providers in Western Kenya attested that they do not often give explanations before conducting procedures on women during childbirth, and 73% do not wait to obtain consent before conducting these examinations.[43] This is similar to the inconsistent support for women's right to autonomy found among Australian midwives and doctors.[44] They confirmed their support for women's autonomy, but override women's decisions sometimes on safety reasons, claiming full accountability for every pregnancy outcome. Women should be included when safety decisions are being made during childbirth. When this is not done, women may conclude it is an abuse of their rights. Tanzanian women related their abusive maternity care experiences as a deviation from their basic human rights.[45] Hence, advocating for women's rights among health providers should be a key component of RMC-promoting interventions.

Nonetheless, the health providers scored high in their perceived IRC_{RMC} and ORC_{RMC}. Few studies had reported the overall organisational readiness for implementing change (ORIC) in health programmes as mean scores using the ORIC tool. Many either report the mean change commitment and change efficacy as individual scores,[21] or as total scores.[31] The ORC_{RMC} score in our study was higher than the average of the change commitment and change efficacy scores found when the nurse-reported ORC for policy change in acute care hospitals in Switzerland was assessed.[21] There was no comparable study of individual readiness for change using the same instrument applied in the health industry. A scoping review to explore the nature and

For peer review only - http://bmjopen.bmj.com/site/about/guidelines.xhtml

extent of literature published on individual readiness for change in the health sector yielded no study found in health.[46]

IRC_{RMC} and ORC_{RMC} in our study were significantly positively correlated. Thus, a positive increase in IRC_{RMC} by strengthening its facilitating factors should also reflect in increased ORC_{RMC}. This is similar to the postulations by Weiner in his theory where he stated that "*Organisational readiness is likely to be highest when organisational members not only want to implement an organisational change but also feel confident that they can do so*" (page 3).[11]

Weiner theorised that organisational readiness was most strongly influenced by change valence and informational assessments.[11] The health providers' change valence positively influenced both their IRC_{RMC} and ORC_{RMC} significantly in our study. Change valence also positively and significantly influenced organisational readiness for change amongst employees of a private hospital changing to a tertiary hospital.[47] It also strongly correlated with individual readiness for change in the automobile industry.[48] There have been limited assessments of individual readiness for change in health-related industries.

Informational assessment is the perceived adequacy of the available resources such as the equipment, expertise, skills, and time needed to implement the change. Informational assessments also significantly influenced both IRC_{RMC} and ORC_{RMC} in this analysis. Informational assessment of their perceived resource adequacy was found to be positively and significantly correlated with their perceived resource availability in this study. This suggests that if providers' perception of resource availability is high, they would be readier for a change to RMC practice. However, the providers had a low perception of the availability of recommended resources for RMC implementation in our study setting. This may have explained their fairly low perceived resource adequacy.

For peer review only - http://bmjopen.bmj.com/site/about/guidelines.xhtml

Thus, additional resource requirements are critical drivers of RMC implementation.[11] For example, only 9% of the health providers agreed that facilities to support birth companions were available. This would include a private space achievable with the use of curtains. In an observational study of childbirths across four countries, Nigeria had the lowest proportion of women (6.9%) in which curtains were used to ensure privacy.[49] This is a challenge that may prevent Nigerian women from receiving RMC as there is limited funding to the Nigerian health system to provide these essential RMC resources. There is a need to identify cost-effective strategies to address these system challenges.

ORC_{RMC} was found to be significantly higher among health providers with longer years of work experience. They are a population to target in RMC-promoting interventions. The nurses' years of work experience also positively influenced their change commitment, one of the measures of organisational readiness, in Switzerland's acute care hospitals.[21] The providers' workplace setting, as indicated by their health facility of practice significantly influenced their perceived $ORC_{RMC.}$ This was significantly positive for most of the primary health care facilities across the LGAs and was significantly negative for two of the secondary health facilities studied. Interestingly, both the primary and secondary health facilities in the Ibadan North-West LGA were significantly associated with a decreased $ORC_{RMC.}$ According to the literature, the workplace contextual fit is critical to providers' readiness for change to RMC as it informs the adaptability of the local context to the globally defined RMC practice, the quality of the implementation, and whether expected RMC implementation outcomes will be achieved.[50–52] There is the need to qualitatively explore which contextual factors within the health facilities are the most critical barriers to a successful implementation of RMC practice during childbirth.

Conclusions

The three most common forms of mistreatment during childbirth noted by health providers corresponded with the low recognition of these as rights that women should always receive.

For peer review only - http://bmjopen.bmj.com/site/about/guidelines.xhtml

Our study confirmed the relevance of the organisational and individual readiness for change constructs to the RMC literature and should prompt more studies on this topic. It is noteworthy that the health providers in our study perceived themselves and their organisations to be ready for a change to RMC practice. It would be important to verify in future research if readiness for change significantly facilitated the implementation of RMC interventions. The main influencing factors of both IRC_{RMC} and ORC_{RMC} scores in our analysis were a high valuation of the change (change valence) and the perceived adequacy of resources necessary to implement the change. Longer serving providers may be a readier population to target during RMC implementation, as champions to lead a change to RMC practice. Workplace contexts could significantly influence ORC_{RMC} and should be explored before the implementation of RMC interventions.

** ** **

Ethics approval and consent to participate: The research was conducted following the Declaration of Helsinki. Ethical approvals were obtained from the Human Research Ethics Committees (HRECs) of the University of the Witwatersrand, Johannesburg (clearance Number M190658), and the Oyo State Ministry of Health (Ref. Number AD/13/479/1386). Written consent to continue the interview was obtained from the respondents. The consent form explained the purpose of the research, and the respondents were asked if they agree to continue with the research or not. The form was filled out using the REDCap software. For anonymity, the facilities were referred to by numbers rather than names. The primary and secondary health facilities were designated with odd and even numbers respectively. Subsequent numbers in sequence are located in the same LGA. Respondents were given a small jotter with brief information on respectful maternity care that costs 0.32USD at ₦380=1USD each on completion of the survey in appreciation of their time and to further educate them. There were

For peer review only

For peer review only - http://bmjopen.bmj.com/site/about/guidelines.xhtml

no inducements given before participation. A transparent and complete reporting of the research was done guided by both the STROBE and CROSS checklists.

Data availability statement

The dataset generated and analysed in the current study is available from the Figshare database (DOI: 10.6084/m9.figshare.19757329).

Competing interests

The authors declare that they have no competing interests.

Funding

This research was supported by the Consortium for Advanced Research Training in Africa (CARTA). CARTA is jointly led by the African Population and Health Research Centre and the University of the Witwatersrand and funded by the Carnegie Corporation of New York (Grant No G-19-57145), Sida (Grant No:54100113), Uppsala Monitoring Center, Norwegian Agency for Development Cooperation (Norad), and by the Wellcome Trust [reference no. 107768/Z/15/Z] and the UK Foreign, Commonwealth & Development Office (No Grant Number), with support from the Developing Excellence in Leadership, Training and Science in Africa (DELTAS Africa) programme (No Grant Number). The statements made and views expressed are solely the responsibility of the Fellow. For open access, the author has applied a CC BY public copyright licence to any 'author accepted' manuscript version arising from this submission. The funders only provided the funding for the research. They played no role in the conceptualisation, design and conduct of the research nor the data analysis, interpretation and development of the manuscript for publication.

Contributors

OTE conceptualised the study, designed the study, acquired and adapted the tools to the study and obtained the ethical approvals. She was the principal investigator who conducted the data collection and supervised the research assistants. She analysed the data and wrote the first draft

For peer review only - http://bmjopen.bmj.com/site/about/guidelines.xhtml

and final manuscripts for publication. SM contributed to the design of the study and the finalisation of the tools. She also significantly contributed to the revision of the draft manuscript and approved the final manuscript for publication. DB contributed significantly to the design of the study, the finalisation of the tools and the data analysis. He significantly revised and contributed significant intellectual content to the draft manuscript and approved the final version of the manuscript for publication.

Acknowledgements

We acknowledge the contributions of Dr Tunde Adedokun, Dr Joshua Akinyemi and Professor Folusho Owotade for their inestimable advice and support with the data analysis. We also recognise and appreciate all the health providers who completed the survey despite their hectic schedules. SM acknowledges the funding support by the South African Medical Research Council's Mid-Career Scientist Award.

References

1. National Population Commission (NPC) [NG] and ICF International. Nigeria Demographic and Health Survey 2013 [Internet]. Abuja, Nigeria and Rockville, Maryland, USA; 2014 [cited 2017 Jun 12]. Available from: https://dhsprogram.com/pubs/pdf/FR293/FR293.pdf

2. Bohren MA, Hunter EC, Munthe-kaas HM, Souza JP, Vogel JP. Facilitators and barriers to facility-based delivery in low- and middle-income countries: a qualitatve evidence synthesis. Reprod Health. 2014;11(71):1–17.

3. Campbell OMR, Calvert C, Testa A, Strehlow M, Benova L, Keyes E, et al. The scale, scope, coverage, and capability of childbirth care [Internet]. Vol. 388, The Lancet. Elsevier Ltd; 2016 [cited 2018 May 9]. p. 2193–208. Available from: http://dx.doi.org/10.1016/S0140-6736(16)31528-8

4. Ogunlaja A, Fehintola O, Ogunlaja I, Popoola G, Idowu A, Awotunde O, et al. " Respectful Maternity Care " or " Disrespect and Abuse during Maternity Care "; Experience of Pregnant Women in Ogbomoso , South West Nigeria. Rwanda Med J. 2017;74(3):6–9.

5. Ishola F, Owolabi O, Filippi V. Disrespect and abuse of women during childbirth in Nigeria: A systematic review. PLoS One [Internet]. 2017 [cited 2018 May 21];12(3):1–17. Available from: http://dx.doi.org/10.1371/journal.pone.0174084

6. World Health Organization. WHO recommendations: Intrapartum care for a positive childbirth experience [Internet]. Geneva, Switzerland; 2018 [cited 2018 Aug 8]. 1–200 p. Available from: https://www.who.int/reproductivehealth/publications/intrapartum-care-guidelines/en/

For peer review only - http://bmjopen.bmj.com/site/about/guidelines.xhtml

7. Williams CR, Meier BM. Ending the abuse: the human rights implications of obstetric violence and the promise of rights-based policy to realise respectful maternity care. Sex Reprod Heal Matters. 2019 Jan 1;27(1).

8. Denny E. Mini commentary: Respectful maternity care needs to be the standard for all women worldwide. BJOG [Internet]. 2018 [cited 2018 May 10];125(8):943. Available from: https://pubmed.ncbi.nlm.nih.gov/29230930/

9. Downe S, Lawrie TA, Finlayson K, Oladapo OT. Effectiveness of respectful care policies for women using routine intrapartum services : a systematic review. Reprod Health. 2018;15(23):1–13.

10. Holt DT, Armenakis AA, Harris SG, Feild HS. Toward a Comprehensive Definiton of Readiness for Change: A Review of Research and Instrumentation. Res Organ Chang Dev [Internet]. 2015 [cited 2019 May 23];16:289–336. Available from: https://www.emeraldinsight.com/doi/abs/10.1016/S0897-3016%2806%2916009-7

11. Weiner BJ. A theory of organizational readiness for change. Implement Sci [Internet]. 2009 [cited 2018 Aug 9];4(67):1–9. Available from: http://www.implementationscience.com/content/4/1/67

12. Lehman WEK, Greener JM, Simpson DD. Assessing organizational readiness for change. J Subst Abuse Treat. 2002;22(4):197–209.

13. Vakola M. What's in there for me? Individual readiness to change and the perceived impact of organizational change. Leadersh Organ Dev J. 2014;35(3):195–209.

14. Toddjasper. EM Defined: Preparedness versus Readiness [Internet]. Emergency Mamagement, Homeland Security, Preparedness. 2012 [cited 2018 Oct 15]. Available from: https://toddjasper.com/2012/02/19/em-defined-preparedness-vs-resdiness/

15. Miake-Lye IM, Delevan DM, Ganz DA, Mittman BS, Finley EP. Unpacking organizational readiness for change: An updated systematic review and content analysis of assessments. BMC Health Serv Res. 2020;20(1):1–13.

16. Haffar M, Al-Karaghouli W, Irani Z, Djebarni R, Gbadamosi G. The influence of individual readiness for change dimensions on quality management implementation in Algerian manufacturing organisations. Int J Prod Econ [Internet]. 2019 [cited 2021 Aug 31];207:247–60. Available from: http://dx.doi.org/10.1016/j.ijpe.2016.08.024

17. Warren CE, Ndwiga C, Sripad P, Medich M, Njeru A, Maranga A, et al. Sowing the seeds of transformative practice to actualize women's rights to respectful maternity care: Reflections from Kenya using the consolidated framework for implementation research. BMC Womens Health. 2017;17(1):1–18.

18. Ratcliffe HL, Sando D, Lyatuu GW, Emil F, Mwanyika-sando M, Chalamilla G, et al. Mitigating disrespect and abuse during childbirth in Tanzania : an exploratory study of the effects of two facility-based interventions in a large public hospital. Reprod Health [Internet]. 2016 [cited 2018 Sep 27];13(79):1–13. Available from: http://dx.doi.org/10.1186/s12978-016-0187-z

19. Kujawski SA, Freedman LP, Ramsey K, Mbaruku G, Mbuyita S, Moyo W, et al. Community and health system intervention to reduce disrespect and abuse during childbirth in Tanga Region, Tanzania: A comparative before-and-after study. PLoS Med. 2017;14(7):1–16.

20. Stata Software. Power and Sample size: Power onemean — Power analysis for a one-

For peer review only - http://bmjopen.bmj.com/site/about/guidelines.xhtml

sample mean test [Internet]. 2019. p. 1–15. Available from: file:///E:/PhD Folder/Respectful maternity care/Very Important for Current PHD Research/psspoweronemean (1).pdf

21. Sharma N, Geest S De, Herrnschmidt J, Claes V, Bachnick S, Simon M. Organizational readiness for implementing change in acute care hospitals : An analysis of a cross - sectional , multicentre study. J Adv Nurs. 2018;74(12):2798–808.

22. Glen S. Design Effect: Definition, Examples [Internet]. Statistics How To. 2015 [cited 2019 Apr 1]. Available from: https://www.statisticshowto.datasciencecentral.com/design-effect/

23. Hameed W, Uddin M, Avan BI. Are underprivileged and less empowered women deprived of respectful maternity care: Inequities in childbirth experiences in public health facilities in Pakistan. PLoS One. 2021;16(4 April):1–17.

24. Shea CM, Jacobs SR, Esserman DA, Bruce K, Weiner BJ. Organizational readiness for implementing change : a psychometric assessment of a new measure. Implement Sci. 2014;9(7):1–15.

25. Judge TA, Erez A, Bono J., Thoresen CJ. The Core Self-Evaluation Scale (CSES): Development of a Measure. Pers Psychol. 2003;56:303–31.

[Dataset] [26]. Esan OT, Maswime S, Blaauw D. Data from: Health providers' readiness for change to a respectful maternity care practice in Ibadan, Nigeria [Internet]. Figshare Data Repository; 2022. Available from: https://figshare.com/s/4c3a01159121780b77da

27. Esan OT, Maswime S, Blaauw D. A qualitative inquiry into pregnant women's perceptions of respectful maternity care during childbirth in Ibadan Metropolis, Nigeria. Sex Reprod Heal Matters. 2022;30(1):1–17.

28. Phillips JE. Effects of Change Valence and Informational Assessments on Organizational Readiness for Change T [Internet]. Walden University, Cape Town; 2017 [cited 2018 Sep 27]. Available from: https://scholarworks.waldenu.edu/dissertations/4016/

29. Win NN, Chotiyaputta V. Measuring Employee Readiness To Change : a Case Study of an Organization in Myanmar. Panyapiwat J. 2018;10(3):110–24.

30. Helfrich CD, Li Y, Sharp ND, Sales AE. Organizational readiness to change assessment (ORCA): Development of an instrument based on the Promoting Action on Research in Health Services (PARIHS) framework. Implement Sci. 2009;4(38):1–13.

31. Geerligs L, Shepherd HL, Butow P, Shaw J, Masya L, Cuddy J, et al. What factors influence organisational readiness for change? Implementation of the Australian clinical pathway for the screening, assessment and management of anxiety and depression in adult cancer patients (ADAPT CP). Support Care Cancer. 2021;29(6):3235–44.

32. Adelson P, Yates R, Fleet JA, McKellar L. Measuring organizational readiness for implementing change (ORIC) in a new midwifery model of care in rural South Australia. BMC Health Serv Res. 2021;21(1):1–6.

33. Leslie HH, West R, Twine R, Masilela N, Steward WT, Kahn K, et al. Measuring Organizational Readiness for Implementing Change in Primary Care Facilities in Rural Bushbuckridge, South Africa. Int J Heal Policy Manag [Internet]. 2020;11(7):912–8.

For peer review only - http://bmjopen.bmj.com/site/about/guidelines.xhtml

Available from: https://doi.org/10.34172/ijhpm.2020.223

34. White Ribbon Alliance. Respectful Maternity Care: The Universal Rights of Childbearing Women [Internet]. The White Ribbon Alliance for Safe Motherhood. 2011 [cited 2021 Feb 1]. Available from: https://www.who.int/woman_child_accountability/ierg/reports/2012_01S_Respectful_Maternity_Care_Charter_The_Universal_Rights_of_Childbearing_Women.pdf

35. Mselle LT, Kohi TW, Dol J. Humanizing birth in Tanzania: A qualitative study on the (mis) treatment of women during childbirth from the perspective of mothers and fathers. BMC Pregnancy Childbirth. 2019;19(1):1–11.

36. Balde MD, Nasiri K, Mehrtash H, Soumah AM, Bohren MA, Diallo BA, et al. Labour companionship and women's experiences of mistreatment during childbirth: Results from a multi-country community-based survey. BMJ Glob Heal. 2020;5(e003564):1–10.

37. Afulani P, Kusi C, Kirumbi L, Walker D. Companionship during facility-based childbirth: results from a mixed-methods study with recently delivered women and providers in Kenya. BMC Pregnancy Childbirth 2018 181 [Internet]. 2018 May 10 [cited 2021 Oct 10];18(1):1–28. Available from: https://bmcpregnancychildbirth.biomedcentral.com/articles/10.1186/s12884-018-1806-1

38. Lawrence A, Lewis L, Hofmeyr GJ, Dowswell T, Styles C. Maternal positions and mobility during first stage labour. Cochrane Database Syst Rev. 2013;(8).

39. Lugina H, Mlay R, Smith H. Mobility and maternal position during childbirth in Tanzania: an exploratory study at four government hospitals. BMC Pregnancy Childbirth 2004 41 [Internet]. 2004 Feb 19 [cited 2021 Oct 10];4(1):1–10. Available from: https://bmcpregnancychildbirth.biomedcentral.com/articles/10.1186/1471-2393-4-3

40. World Health Organization. Standards for improving quality of maternal and newborn care in health facilities [Internet]. Geneva,, Switzerland: WHO Press; 2016 [cited 2018 Oct 16]. 84 p. Available from: https://www.who.int/maternal_child_adolescent/documents/improving-maternal-newborn-care-quality/en/

41. WHO | Why having a companion during labour and childbirth may be better for you. WHO [Internet]. 2019 [cited 2021 Oct 10]; Available from: http://www.who.int/reproductivehealth/companion-during-labour-childbirth/en/

42. Mirzaee Rabor F, Taghipour A, Najmabadi KM, Fattahi Masoum SH, Pour MF. Respect to women's autonomy in childbirth: A qualitative study. Iran Red Crescent Med J. 2017;19(12).

43. Afulani PA, Afulani PA, Buback L, Kelly AM, Kirumbi L, Cohen CR, et al. Providers' perceptions of communication and women's autonomy during childbirth: A mixed methods study in Kenya. Reprod Health. 2020 Jun 3;17(1).

44. Edwards N. Maternity care providers' perceptions of women's autonomy and the law. AIMS J. 2015;27(1):22.

45. Miltenburg AS, Lambermon F, Hamelink C, Meguid T. Maternity care and Human Rights: what do women think? BMC Int Health Hum Rights [Internet]. 2016 [cited

For peer review only - http://bmjopen.bmj.com/site/about/guidelines.xhtml

2018 Jun 16];16(17):1–10. Available from: http://dx.doi.org/10.1186/s12914-016-0091-1

46. Beasley L, Grace S, Horstmanshof L. Assessing individual readiness for change in healthcare: a review of measurement scales. J Health Organ Manag [Internet]. 2021 Jan 1 [cited 2021 Mar 10];35(8):1062–79. Available from: https://doi.org/10.1108/JHOM-10-2020-0414

47. Trisnawati A, Damayanti N, Novita RD. How Change Valence Impacts Readiness to Change In Teaching Hospital. Eur J Mol Clin Med. 2020;07(05):310–6.

48. AbuTahoun TNB, Khan N. The Management Role in Promoting Individual Readiness for Change. Int Conf Manag Inf Syst [Internet]. 2019 [cited 2021 Jul 28];29:217–22. Available from: https://search.ebscohost.com/login.aspx?direct=true&db=buh&AN=141539752&site=ehost-live

49. Asefa A, McPake B, Langer A, Bohren MA, Morgan A. Imagining maternity care as a complex adaptive system: understanding health system constraints to the promotion of respectful maternity care. Sex Reprod Heal Matters [Internet]. 2020 [cited 2021 Oct 11];28(1):456–74. Available from: https://doi.org/10.1080/26410397.2020.1854153

50. Edwards N, Barker PM. The importance of context in implementation research. J Acquir Immune Defic Syndr. 2014;67:S157–62.

51. Horner R, Blitz C, Ross SW. The Importance Of Contextual Fit When Implementing Evidence-Based Interventions [Internet]. ASPE Issue Brief. 2014 [cited 2021 Nov 27]. Available from: http://aspe.hhs.gov/hsp/14/IWW/ib_Contextual.pdf

52. Kok BC De, Uny I, Immamura M, Bell J, Geddes J, Phoya A. From Global Rights to Local Relationships : Exploring Disconnects in Respectful Maternity Care in Malawi. Qual Health Res. 2020;20(3):341–335.

Figure 1: Forms of mistreatment and perceived providers' rights to women during childbirth

Figure 2: Provider perceptions on availability of WHO-recommended resources for RMC implementation

For peer review only

For peer review only - http://bmjopen.bmj.com/site/about/guidelines.xhtml

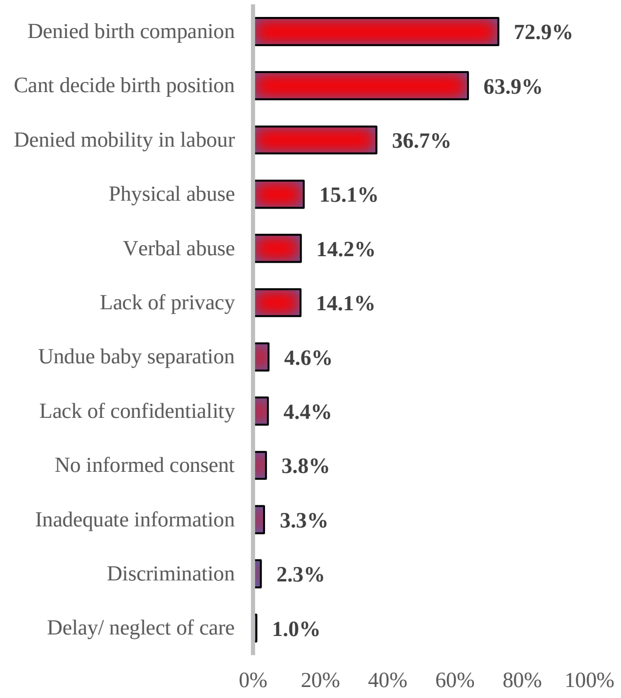

Forms of mistreatment noted by providers in their health facilities (n=212)

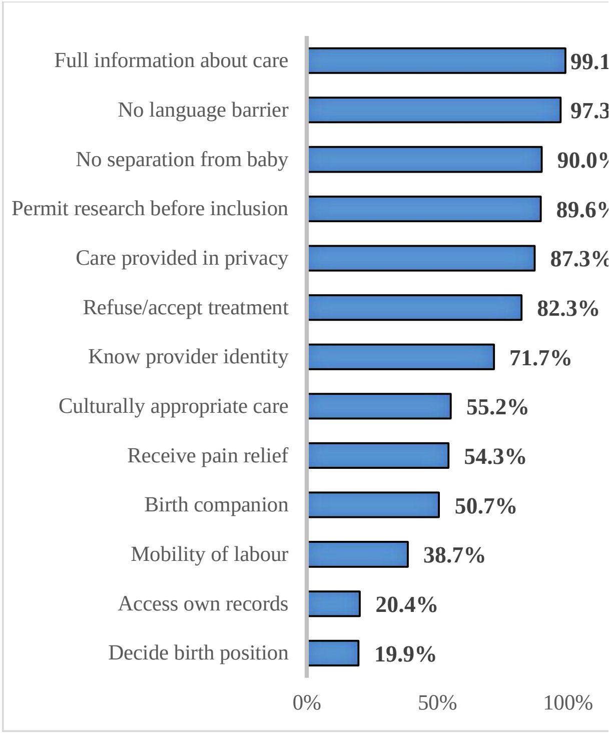

Provider’s perceived rights women should always have during childbirth (n=212)

For peer review only

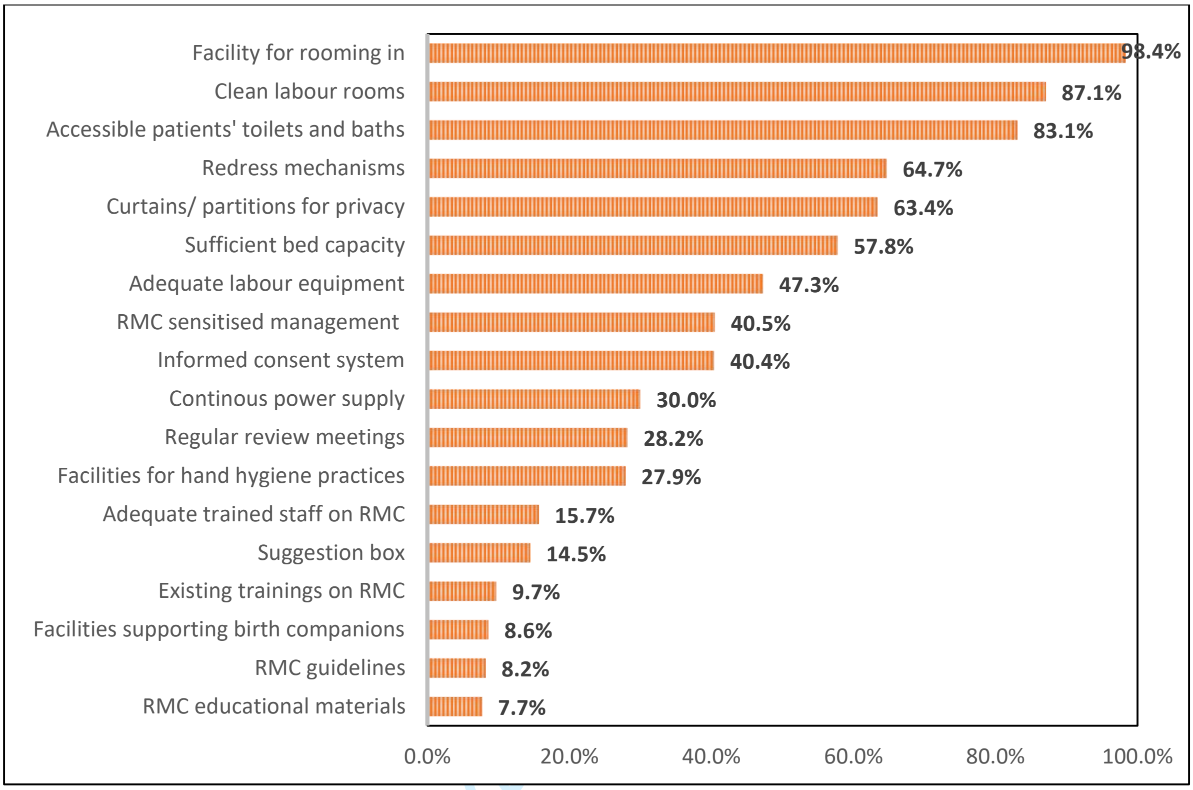
Facility for rooming in
98.4%
Clean labour rooms
87.1%
Accessible patients' toilets and baths
83.1%
Redress mechanisms
64.7%
Curtains/ partitions for privacy
63.4%
Sufficient bed capacity
57.8%
Adequate labour equipment
47.3%
RMC sensitised management
40.5%
Informed consent system
40.4%
Continous power supply
30.0%
Regular review meetings
28.2%
Facilities for hand hygiene practices
27.9%
Adequate trained staff on RMC
15.7%
Suggestion box
14.5%
Existing trainings on RMC
9.7%
Facilities supporting birth companions
8.6%
RMC guidelines
8.2%
RMC educational materials
7.7%
0.0%
20.0%
40.0%
60.0%
80.0%
100.0%

Additional file 1

What is Respectful Maternity Care?

Respectful Maternity Care (RMC) is a human rights approach to childbirth care practice. It is a new strategy for caring for women in labour which we are yet to commence implementing in this health facility. We are interested in knowing how health facilities offering childbirth
services, their managers and individual workers are READY to integrate respectful maternity care into their routine childbirth services.

What is Respectful Maternity care?
Simply, it means the following

1. The preferences of the client must be respected, and she must be involved in the decision making regarding her health.
2. She must be allowed a companion during birth as recommended by the WHO
3. She must be free to move about during labour if she so wishes even in the second stage before the urge to deliver and not restricted to one position.
4. If classified as a low risk pregnant woman, she should be allowed oral fluids or food while in labour as evidence has shown no negative outcomes following this.
5. Her privacy must be ensured by providing one private cubicle or space per woman in labour and information about her should not be shared openly.
6. If she prefers to deliver her child squatting, the health care provider must be willing to support her in the decision.
7. Equitable services must be delivered to her regardless of her personal characteristics.
8. When she calls for help during labour, she must not be denied nor neglected.
9. If she is unable to pay her bills, a consensus must be reached with her on how to pay rather than detaining her illegally for the inability to pay.
10. Overall, she must receive the utmost respectful and dignified care, that she deserves as her fundamental human rights.

For peer review only

Rmc Readiness Assessment Of Health Care Providers

Record ID	__________________
Longitude	__________________
Latitude	__________________
Time of start of interview	__________________
1. RMC Record ID (For example Adeoyo/HW/001)	__________________
2. Which Local Government Area (LGA) is this facility situated	◯ Ibadan North<br>◯ Ibadan North East<br>◯ Ibadan North West<br>◯ Ibadan South East<br>◯ Ibadan South West
3. Level of Public Health Facility	◯ Primary ◯ Secondary
4. Date of Birth (Example Day-Month-Year	__________________
Today's date	__________________
5. Age	__________________
6. Highest Level of education completed	◯ Secondary education<br>◯ School of Health Technology<br>◯ Nursing School<br>◯ Nurse/Midwifery School<br>◯ Medical School<br>◯ Master's program / Part 1 Fellowship Exams<br>◯ Ph.D. program/ Part 2 Fellowship Exams
7. Professional type	◯ Specialist Consultant<br>◯ Resident doctor<br>◯ Medical Officer<br>◯ House Officer<br>◯ Nurse<br>◯ Nurse/Midwife<br>◯ CHO<br>◯ CHEW<br>◯ Health Assistant/ Nurse Auxilliary/ Nurse Aide
In which sub-unit are you working in this health facility or do you work across all the units?	◯ I work in the antenatal unit only<br>◯ I work in the labour room only<br>◯ I work in the post-natal unit only<br>◯ I work in all the units

For peer review only

8. Your total income in a month (from all sources) may fall within which of these ranges	◯ < ₦30,000 (83.3USD)<br>◯ ₦30, 000 - ₦50,000 (138.9 USD)<br>◯ ₦51,000 - ₦70,000 (194.4 USD)<br>◯ ₦71, 000 - ₦90,000 (250 USD)<br>◯ ₦91,000 - ₦110,000 (305.5 USD)<br>◯ ₦110,000- ₦200,000 (555.5 USD)<br>◯ ₦200,000 - ₦400,000 (1,111 USD)<br>◯ More than ₦400,000 (1,111 USD)
9. Specifically, these total Income from (all sources) per month will be about ___ in Naira (write only in figures)	__________________________________
10. The year you completed pre-service training (graduated from school) for your current profession	__________________________________
11. Years of professional experience after pre-service training	__________________________________
12. The year you started working in this facility as one of the following (nurse/midwife//CHO/CHEW)	__________________________________
13. Years of experience working in this facility as one of the following (nurse/midwife/doctor/CHO/CHEW)	__________________________________
14. Have you ever been promoted in your current job?	◯ Yes ◯ No
15. In which year were you last promoted in your current job (OR Moved to the next level or higher level for doctors?	__________________________________
16. No of years ago you were last promoted	__________________________________
17. The current level of the respondent in the Oyo State civil service employment (based on ranks like level 12. Write only the figure, eg. 12)	__________________________________
18. Sex of the health care provider	◯ Male ◯ Female

Which of these do you think women should have as a Fundamental Human Right to during childbirth and in what frequency? If they should not have a right to it, please, select "Never". If they should, then select best of the other options. (A right is something they can demand for if not given)

	Always (Nigbogbo igba)	Very frequently (Ni lemolemo)	Occasionally (Ni eknankan)	Rarely (Ko wopo rara)	Never (Ko sele ri)

For peer review only

1. Right to know / ask about the professional identity and qualifications of those involved with her care	◯	◯	◯	◯	◯
2. Right to communicate with caregivers and receive all care in privacy	◯	◯	◯	◯	◯
3. Right to full and clear information about what is being done for her and their benefits, risks and costs (on the procedures, drugs, tests and treatments offered to her)	◯	◯	◯	◯	◯
4. Right to accept or refuse procedures, drugs, tests and treatments, and to have her choices honored	◯	◯	◯	◯	◯
5. Right to be informed if her caregivers wish to enroll her or her infant in a research study	◯	◯	◯	◯	◯
6. Right to unrestricted access to her file and all available records about her pregnancy, labor, birth, postpartum	◯	◯	◯	◯	◯
7. Right to receive maternity care that is appropriate to her cultural and religious background.	◯	◯	◯	◯	◯
8. Right to receive information in a language in which she can communicate (like getting her an interpreter) where necessary	◯	◯	◯	◯	◯
9. Right to have a family member of her choice present during all the aspects/ stages of her labour and childbirth (having a birth companion)	◯	◯	◯	◯	◯
10. Right to freedom of movement during labor, not hindered by tubes, wires or other apparatus	◯	◯	◯	◯	◯

For peer review only

11. Right to virtually uninterrupted contact with her newborn from the moment of birth, as long as she and her baby are healthy and do not need care that requires separation	○	○	○	○	○
12. Right to receive a pain relief during labour with information on the type of pain relief, and the risks and benefits	○	○	○	○	○
13. Right to deciding the position of birth (Squatting)	○	○	○	○	○

Has any of the following ever been done to women who have come to deliver in this hospital in the past 1 year to the best of your knowledge (not necessarily by you)? Kindly select the appropriate response

	Always	Very frequently	Occasionally	Rarely	Never
1. Physical abuse (slapping or hitting pinching, beating or attempts made to)	○	○	○	○	○
2. Verbal abuse (saying sentences to pregnant women in labour that may sound abusive like "was I there when you were getting impregnated""?)	○	○	○	○	○
3. Not providing information on the care to be provided or the procedure to be done before doing it	○	○	○	○	○
4. Not obtaining informed consent or a go ahead before procedures or examinations are done (even if information was provided about it)	○	○	○	○	○
5. Lack of privacy (not screening during examinations)	○	○	○	○	○
6. Lack of confidentiality (discussing patients' details openly or patients' files kept indiscriminately)	○	○	○	○	○
7. Discrimination based on age, economic / financial status, ethnicity, religion	○	○	○	○	○

For peer review only

For peer review only - http://bmjopen.bmj.com/site/about/guidelines.xhtml

8. A birth companion of her choice NOT allowed to be present with the woman all through the stages during labour	◯	◯	◯	◯	◯
9. Women not allowed to move about during labour but must lie down in one position	◯	◯	◯	◯	◯
10. Women not given an option to choose a position of birth (like squatting to deliver) or denial of their choice	◯	◯	◯	◯	◯
11. Unnecessary separation of mother and newborn after the birth	◯	◯	◯	◯	◯
12. Leaving the woman alone or unattended or delaying attending to the woman after being called / sent for	◯	◯	◯	◯	◯

13. Other types of similar actions done to women during childbirth in this health facility, please, specify ______________________

How long are women kept on admission at the facility after an uncomplicated delivery on the average before discharge home?
- ◯ Less than 6 hours
- ◯ 6-12 hours
- ◯ More than 12 hours to a maximum of 24 hours
- ◯ More than 24 hours
- ◯ Don't know

Have you ever heard of Respectful Maternity Care (RMC) before? ◯ Yes ◯ No

Readiness Assessment for a Change to a Respectful Maternity Care Practice

Respectful Maternity Care (RMC) is a human rights approach to childbirth care practice. It is a new strategy for caring for women in labour which we are yet to commence implementing in this health facility. We are interested in knowing how health facilities offering childbirth services,
their managers and individual workers are READY to integrate respectful maternity care into their routine childbirth services.
What is Respectful Maternity care?
Simply, it means the following
1. The preferences of the client must be respected, and she must be involved in the decision making regarding her health.
2. She must be allowed a companion during birth as recommended by the WHO
3. She must be free to move about during labour if she so wishes even in the second stage before the urge to deliver and not restricted to one position.
4. If classified as a low risk pregnant woman, she should be allowed oral fluids or food while in labour as evidence has shown no negative outcomes following this.
5. Her privacy must be ensured by providing one private cubicle or space per woman in labour and information about her should not be shared openly.

For peer review only

6. If she prefers to deliver her child squatting, the health care provider must be willing to support her in the decision.
7. Equitable services must be delivered to her regardless of her personal characteristics.
8. When she calls for help during labour, she must not be denied nor neglected.
9. If she is unable to pay her bills, a consensus must be reached with her on how to pay rather than detaining her illegally for the inability to pay.
10. Overall, she must receive the utmost respectful and dignified care, that she deserves as her fundamental human rights.

Respectful Maternity Care can be implemented in this health facility	◯ Strongly disagree<br>◯ Somewhat disagree<br>◯ Neither agree nor disagree<br>◯ Somewhat agree<br>◯ Strongly agree

Organizational Readiness for a change to RMC Practice

Please select one option for each of these sentences below that best describes how much you think your health facility management and staff are READY to integrate respectful maternity care (RMC) practice into the routine childbirth/ delivery care services in this facility (Please, tick one of these 5 options: Strongly Disagree=D, Somewhat Disagree= SWD, Neither Agree nor Disagree =NA/D, Somewhat Agree= SWA, Strongly Agree= A)

	Strongly Disagree	Somewhat disagree	Neither agree nor disagree	Somewhat agree	Strongly Agree
6. Health workers in this health facility want to (will not mind to) implement this change to RMC practice.	◯	◯	◯	◯	◯
2. Health workers in this health facility are committed to implementing this change to RMC practice	◯	◯	◯	◯	◯
9. Health workers in this health facility are determined to implement this change to RMC practice.	◯	◯	◯	◯	◯
4. Health workers in this health facility will do whatever it takes to implement this change to RMC practice.	◯	◯	◯	◯	◯
11. Health workers in this health facility are self-motivated to implement this change to RMC practice.	◯	◯	◯	◯	◯

For peer review only

1. Health workers in this health facility feel confident that the facility management can get people invested in implementing this change to RMC practice.	○	○	○	○	○
5. Health workers in this health facility feel confident that the facility managers can support people to adjust to this change to RMC practice.	○	○	○	○	○
7. Health workers in this health facility feel confident that they can keep pushing to implementing this change to RMC practice.	○	○	○	○	○
3. Health workers in this health facility feel confident that they can keep track of progress in implementing this change to RMC practice.	○	○	○	○	○
10. Health workers in this health facility feel confident that they can coordinate tasks so that implementation goes smoothly to RMC practice.	○	○	○	○	○
8. Health workers in this health facility feel confident that they can handle the challenges that might arise in implementing this change to RMC practice.	○	○	○	○	○
12. Health workers in this health facility feel confident that they can manage the politics of implementing this change to RMC practice.	○	○	○	○	○

The score for commitment to change ________________________________

The MEAN score for Change commitment ________________________________

The score for Change Efficacy ________________________________

The MEAN Score for Change Efficacy ________________________________

For peer review only

Informational assessment- Regarding a change to a respectful maternity care practice being integrated into your routine childbirth care, please, select the most appropriate option to the sentences below

	Strongly disagree	Somewhat disagree	Neither agree nor disagree	Somewhat agree	Strongly agree
1. Health workers in this health facility believe we have the equipment we need to implement this change to a RMC practice	○	○	○	○	○
2. Health workers in this health facility believe we have the expertise we need to implement this change to RMC practice	○	○	○	○	○
3. Health workers in this health facility believe we have the time we need to implement this change to a RMC practice	○	○	○	○	○
4. Health workers in this health facility believe we have the skills we need to implement this change to a RMC practice	○	○	○	○	○
5. Health workers in this health facility believe we have the resources we need to implement this change to a RMC practice	○	○	○	○	○
6. Health workers in this health facility know how much time it will take to implement this change	○	○	○	○	○
7. Health workers in this health facility know what resources we will need to implement this change	○	○	○	○	○
8. Health workers in this health facility know what each of us has to do to implement this change Given	○	○	○	○	○

For peer review only

Change Valence Assessment-
Please select the most appropriate option for each of these statements below regarding a change to integrate respectful maternity care (RMC) practice into the routine childbirth/ delivery care services in this facility.

	Strongly disagree	Somewhat disagree	Neither agree nor disagree	Somewhat agree	Strongly agree

1. Health workers in this health facility feel that a change to a respectful maternity care is compatible with our values	○	○	○	○	○
2. Health workers in this health facility feel that we need to implement this change to RMC practice	○	○	○	○	○
3. Health workers in this health facility believe this change to RMC practice will benefit our community	○	○	○	○	○
4. Health workers in this health facility believe this change to RMC practice will make things better	○	○	○	○	○
5. Health workers in this health facility believe this change to RMC practice is a good idea	○	○	○	○	○
6. Health workers in this health facility value this change to a RMC practice	○	○	○	○	○

Individual Readiness for Change Assessment Tool

The options below are assessing your own personal readiness for your health facility to integrate respectful maternity care practices into your routine childbirth care. Select one option each

	Strongly disagree	Disagree	Slightly disagree	Do not disagree nor agree	Slightly Agree	Agree	Strongly Agree
1. When changes such as a change to RMC practice is about to occur in our health facility, I believe that I am ready to cope with them.	○	○	○	○	○	○	○
2. I usually try to convince people in my health facility to accept the change such as a change to RMC practice	○	○	○	○	○	○	○
3. When changes occur in my health facility, I tend to complain about them rather than deal with them*	○	○	○	○	○	○	○

For peer review only

4. I believe that I am more ready to accept a change to RMC practice than my colleagues.	○	○	○	○	○	○	○
5. I don't worry about changes in my health facility because I believe that there is always a way to cope with them	○	○	○	○	○	○	○
6. When changes such as a change to RMC practice occur in my company, I always have the intention to support them.	○	○	○	○	○	○	○

Concerning the availability of the resources needed to integrate a respectful maternity care practice into the routine childbirth care in this facility, please select the best option

	Disagree	Somewhat disagree	Neither agree nor disagree	Somewhat agree	Agree
1. We have adequate number of trained and competent skilled birth attendants (e.g. nurse, doctors and nurse auxiliaries like CHO/CHEWS) that can provide RMC care to all women	○	○	○	○	○
2. The Management of our health facility are very familiar and well sensitized to the provision of a respectful maternity care practice	○	○	○	○	○
3. Regular practical, in-service training on the provision of respectful maternity care is being done for the staff in this health facility	○	○	○	○	○
4. There are written, up-to-date guidelines on the provision of a respectful maternity care during childbirth in this health facility	○	○	○	○	○
5. There are established and standard informed consent system in place and forms to be used for procedures requiring such (for example episiotomy	○	○	○	○	○

For peer review only

6. There are health education materials on respectful maternity care in pictures and the languages of the communities served in this health facility

7. Rooming-in (Nursing baby by the mother's side) is practiced in this facility to allow women and their babies to remain together

8. There are clean, appropriately illuminated, well ventilated labour, and childbirth areas

9. There are clean and accessible toilets and bathrooms for use by women in labour/ post delivery

10. There is safe drinking water, and a hand washing station, with soap and water (preferably running water) or alcohol-based hand rubs

11. Curtains, screens, partitions are available and are being used to maintain privacy for women during ;labour and childbirth

12. There are sufficient bed capacity (needed number of beds for facility)

13. Facilities for companions of women in labour, including a private space (or partitioned with curtain) that can allow for the woman and her companion are available

14. Basic and adequate equipment for labor and childbirth that is available in sufficient quantities at all times in the labor and childbirth areas

15. Continuous energy/ power supply in the labor, childbirth and neonatal areas

For peer review only

16. Staff meetings are held regularly to review our childbirth practices if respectful (RMC practices) or not in this health facility	◯	◯	◯	◯	◯
17. There is a suggestion box for service users (clients/ patients) and providers to submit complaints to the management	◯	◯	◯	◯	◯
18. Establishment of accountability mechanisms for redress in the event of mistreatment or violations (eg. disciplinary committee to handle report of mistreatment of women or providers)	◯	◯	◯	◯	◯

These last set of questions help us to self-evaluate ourselves as a person and may elicit the factors that are associated with our readiness for a change to a respectful maternity care

Health care providers' Core Self-Evaluation Personality Traits- Kindly select the most appropriate option to each of the sentences below as it describes you as a person

	Strongly disagree	Somewhat disagree	Neither agree nor disagree	Somewhat agree	Strongly agree
1. I am confident I'll get the success I deserve in life.	◯	◯	◯	◯	◯
2. Sometimes I feel miserable*	◯	◯	◯	◯	◯
3. When I try, I generally succeed	◯	◯	◯	◯	◯
4. Sometimes when I fail I feel worthless*	◯	◯	◯	◯	◯
5. I complete tasks successfully	◯	◯	◯	◯	◯
6. Sometimes, I do not feel I am in control of my work*	◯	◯	◯	◯	◯
7. Overall, I am satisfied with myself	◯	◯	◯	◯	◯
8. I am filled with doubts about my competence*	◯	◯	◯	◯	◯
9. I determine / choose what will happen in my life	◯	◯	◯	◯	◯
10. do not feel in control of my success in my career*	◯	◯	◯	◯	◯

For peer review only

11. I am capable of coping with most of my problems	○	○	○	○	○
12. There are times when things look pretty bleak and hopeless to me*	○	○	○	○	○

Health care providers' Job satisfaction- Regarding the extent to which you are satisfied with your job, kindly select the most appropriate option for the sentences below

	Strongly disagree	Somewhat disagree	Neither agree nor disagree	Somewhat Agree	Strongly agree
1. My salary is fair compared to other staff in other southwest states with the same level of responsibility	○	○	○	○	○
2. My benefits are fair compared to other staff at my level	○	○	○	○	○
3. My job description is clear to me, accurate and up to date	○	○	○	○	○
4. My supervisor and I have agreed on the priorities of my job	○	○	○	○	○
5. I get clear feedback from my supervisors about how well I am performing on my job	○	○	○	○	○
6. My annual performance appraisal is based on the priorities in my workplan (my actual performance)	○	○	○	○	○
7. My supervisor seeks my input when faced with a challenge or problem	○	○	○	○	○
8. The organization (the management of this facility) acknowledges and values my work	○	○	○	○	○
9. The organization (the State Ministry of Health) provide me with the essential coaching and training to do my job.	○	○	○	○	○
10. The organization works (as much as possible) to provide me with opportunities for career growth.	○	○	○	○	○

Any other important comments or observations ______________________________

Time to END of interview ______________________________

For peer review only

Name of Interviewer	◯ Bukola<br>◯ Dr. Esan<br>◯ Any other

For peer review only

Additional file 3

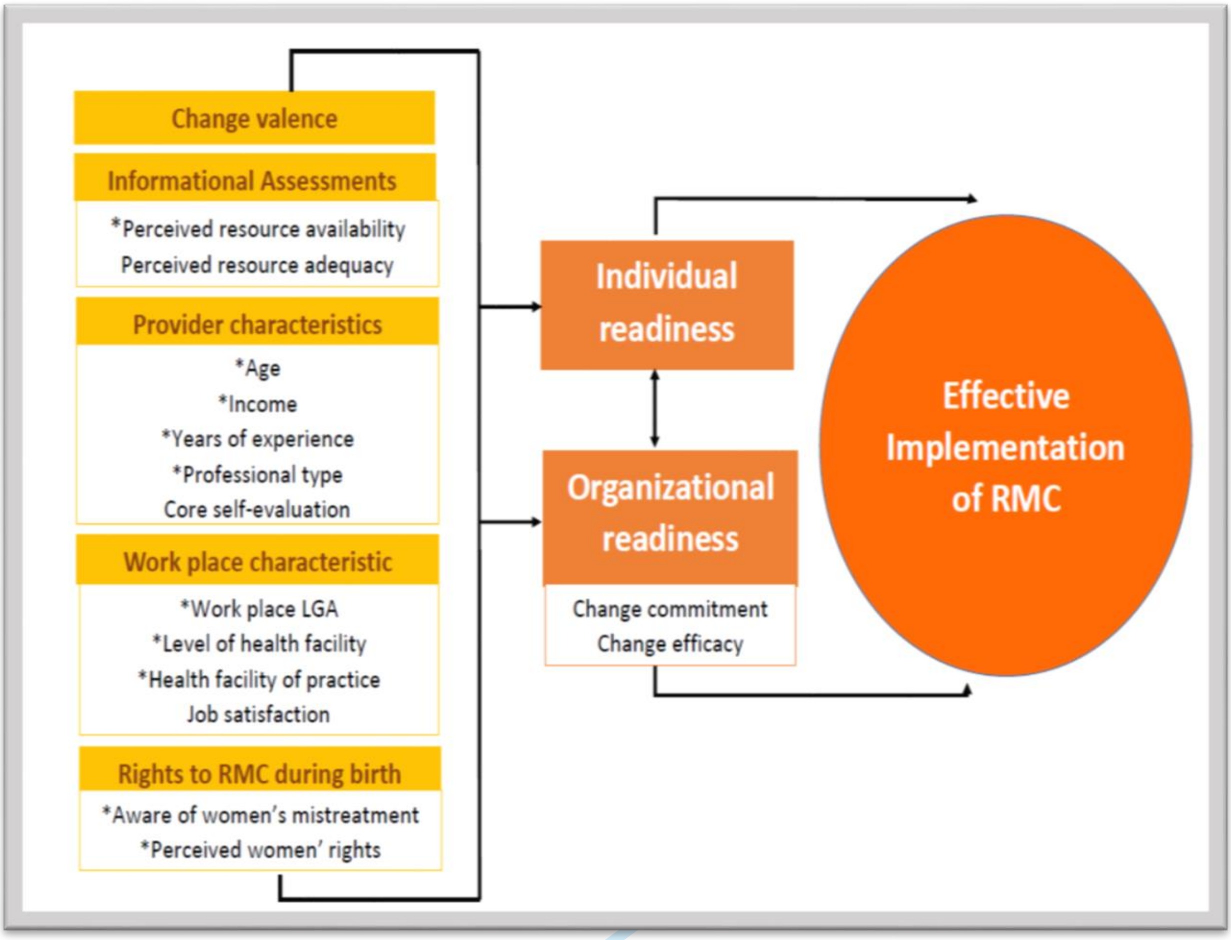

Study Analytical Frameworks (Note: *proposed as predictor variables)

Additional file 4: Breakdown of tools in the health provider survey instrument

	Name of Tool and Sections	Source	Items	Response type	Alpha coeff.
1	*Organisational Readiness for Implementing change (ORIC)	Shea et al [19]	12	Likert scale 1-5	0.949
2	*Individual readiness for change	Vakola et al[8]	6	Likert scale 1-7	0.733
3	Socio-demographic characteristics	Adapted from the literature	15		
4	Perception on women's rights during childbirth	Childbirth Connection[20]	13	Likert scale 1-5	0.575
5	Provider awareness of mistreatment in their own facility	Maternal & Child Health program[21]	12	Likert scale 1-5	0.638
6	Change valence	Shea et al[19]	6	Likert scale 1-5	0.902
7	Informational assessments	Phillip[22]	8	Likert scale 1-5	0.648
8	Perception on RMC resource availability	WHO Recommendation for labour[1]	18	Likert scale 1-5	0.669
10	Core self-evaluation tool	Judge et al[18]	12	Likert scale 1-5	0.598
11	Employee job satisfaction tool	Management Sciences for Health[23]	10	Likert scale 1-5	0.603
	Total		112		

*Outcome variables; WHO: World Health Organisation

For peer review only

Additional file 5: Summarised PCA Findings

1. Principal Component Analsysis Results for the scale assessing Health Provider's perception of women's rights during childbirth.

Test	Measure	Statistics
Kaiser-Meyer-Olkin (KMO)	Sampling adequacy	[a]0.620
Bartlett's	Sphericity	$\chi^2 = 229.126$
		df = 78
		$p < 0.001$*

Total Variance explained

Component	Eigenvalues	% of variance	Cumulative %
1	2.326	17.89	17.89
2	1.448	11.14	29.04

* Statistically significant [a] Sample is adequate (No of observations -203)

Principal component eignevectors for 4 of 13 components

Variable	Comp1	Comp2	Comp3	Comp4	Comp5
right_Know~r	0.3444	-0.0563	0.0809	0.4687	0.1214
right_priv~y	0.2609	-0.5047	0.0694	0.1062	0.1655
right_info~n	0.1528	-0.1522	0.1208	0.0868	0.6739
right_refu~t	0.1797	0.3353	-0.2941	0.3634	0.0657
right_rese~t	0.2260	0.4095	-0.3549	0.1507	0.2613
right_acce~s	0.2397	-0.2237	-0.3648	-0.3109	0.1185
right_cult~e	0.3100	-0.3255	0.0846	0.2425	-0.4273
right_nola~r	0.0559	0.2120	0.7022	-0.0018	0.2357
right_comp~p	0.3543	0.0971	0.3081	-0.0594	-0.2283
right_mobi~r	0.3228	-0.0195	-0.0627	-0.3274	0.1690
right_noba~n	0.1915	0.4536	0.1623	-0.2516	-0.0634
right_pain~f	0.4288	0.1441	-0.0478	0.0854	-0.3068
right_birt~n	0.3157	-0.0396	-0.0281	-0.5196	0.0064

Correlation matrix showing the total, mean scores and pca predicted scores (final rights) for health provider's perception and frequency of women's rights during childbirth.

	Total scores _perceived women's rights	Mean scores _perceived women's rights	No of items agreed to_ _perceived women's rights	Pca scores _perceived women's rights
Total scores _	1.000			
Mean scores	1.000	1.000		
No of items agreed	0.9157	0.9157	1.000	
Pca scores	0.9919	0.9919	0.9073	1.000

For peer review only

2. **Principal Component Analsysis Results for the scale assessing Health Provider's awareness of the frequency of mistreatment f o women during childbith at their own health facilities**

Test	Measure	Statistics
Kaiser-Meyer-Olkin (KMO)	Sampling adequacy	[a]0.720
Bartlett's	Sphericity	χ^2 = 357.784
		df = 66
		p < 0.001*

Total Variance explained

Component	Eigenvalues	% of variance	Cumulative %
1	2.783	23.19	23.19
2	1.440	12.00	35.19

* Statistically significant [a] Sample is adequate (No of observations -211)

Principal component eignevectors for 4 of 12 components

Variable	Comp1	Comp2	Comp3	Comp4
physical_a~e	0.1505	-0.1880	0.5230	0.3352
verbal_abuse	0.3260	-0.3530	0.3263	0.1119
lack_of_in~n	0.4237	-0.0759	-0.2696	0.2439
no_informe~t	0.2234	0.1038	-0.4436	0.4205
lack_of_pr~y	0.3758	0.2156	0.0596	-0.2068
lack_confi~y	0.4621	0.0288	-0.1338	-0.1383
discrimina~n	0.4057	-0.1312	-0.1291	-0.0910
no_birth_c~n	0.1262	0.3171	0.2598	0.2618
no_movt_in~r	0.0401	0.4763	0.3472	-0.1348
no_choice_~n	0.1061	0.5535	0.1156	0.3253
separation~y	0.1021	-0.3402	0.3182	0.0749
abandonmen~e	0.2883	0.0975	0.0988	-0.6087

Correlation matrix showing the total scores, mean scores and pca predicted scores (final mistreatment) for health provider's awareness of the frequency of women's mistreatment during childbirth at their facilities

	Total scores _perceived women's rights	Mean scores _perceived women's rights	No of items agreed to_ _perceived women's rights	Pca scores _perceived women's rights
Total scores _	1.000			
Mean scores	1.000	1.000		
No of items agreed	-0.7437	-0.7437	1.000	
Pca scores	0.8658	0.8658	-0.4638	1.000

For peer review only - http://bmjopen.bmj.com/site/about/guidelines.xhtml

For peer review only

3. Principal component analysis results for the scale assessing health provider's perception of resource availability for the implementation of RMC as recommended by the World Health Organisation

Test	Measure	Statistics
Kaiser-Meyer-Olkin (KMO)	Sampling adequacy	[a]0.640
Bartlett's	Sphericity	$\chi^2 = 557.535$
		df = 153
		$p < 0.001$*

Total Variance explained

Component	Eigenvalues	% of variance	Cumulative %
1	2.968	16.49	16.49
2	2.242	12.46	28.95

* Statistically significant [a] Sample is adequate (No of observations -173)

Principal component eignevectors for 6 of 18 components

Variable	Comp1	Comp2	Comp3	Comp4	Comp5	Comp6
adequate_s~f	0.1937	-0.3240	0.2060	-0.0295	0.1330	-0.1381
mgt_sensit~d	0.1834	0.1923	-0.4623	-0.0580	0.1970	-0.0811
regular_rm~g	0.3397	-0.0619	-0.3633	-0.2583	-0.1123	-0.0819
written_gu~s	0.3728	-0.0247	0.0683	-0.3796	-0.1623	0.0967
informed_c~t	0.0625	0.5407	0.1832	-0.1032	0.0221	0.0626
rmc_educat~s	0.2679	0.0166	0.3492	-0.3203	-0.1146	0.1170
rooming_in	-0.0196	0.2111	-0.0005	0.0803	-0.0010	0.6030
clean_priv~e	0.2048	0.2019	-0.1084	0.3604	-0.2740	0.0965
clean_bath~s	0.2307	0.1783	0.0003	0.3818	-0.3967	0.0724
safe_water~e	0.0780	0.1868	0.1777	0.2380	0.0806	-0.6417
curtains_a~s	0.3270	0.1311	0.1233	0.1952	-0.1454	-0.2312
adequate_b~s	0.2094	-0.1672	0.2842	0.2932	0.4128	0.1370
space_woma~s	0.1107	-0.3020	0.3363	-0.0534	-0.3750	-0.0117
adequate_l~t	0.1908	-0.3234	-0.0153	0.3417	0.2592	0.2372
power_supp~r	0.3325	-0.0968	-0.0659	0.0334	-0.0543	0.1038
rmc_practi~w	0.2486	-0.2531	-0.4220	0.0271	0.0132	-0.0762
suggestion~x	0.2576	0.2220	0.1264	-0.2739	0.4053	-0.0240
redress_co~e	0.2430	0.2129	0.0257	0.1119	0.2920	0.0707

Correlation matrix showing the total scores, mean scores and principal component analysis predicted scores (final res) predicted scores for health provider's perception on the availability of resources needed to implement respectful maternity care as recommended by the World Health Organisation

	Total scores _resource availability	Mean scores _ resource availability	No of items agreed to_ _ resource availability	Pca scores _ resource availability
Total scores _	1.000			
Mean scores	1.000	1.000		
No of items agreed	0.9206	0.9206	1.000	
Pca scores	0.9576	0.9576	0.8657	1.000

For peer review only - http://bmjopen.bmj.com/site/about/guidelines.xhtml

STROBE Statement—Checklist of items that should be included in reports of ***cross-sectional studies***

Title: Organisational and individual readiness for change to respectful maternity care practice and associated factors in Ibadan, Nigeria: a cross-sectional study

	Item No	Recommendation		Page
**Title and abstract**	1	(*a*) Indicate the study's design with a commonly used term in the title or the abstract	✓	1
		(*b*) Provide in the abstract an informative and balanced summary of what was done and what was found	✓	2
**Introduction**				
Background/rationale	2	Explain the scientific background and rationale for the investigation being reported	✓	4
Objectives	3	State specific objectives, including any pre-specified hypotheses	✓	5
**Methods**				
Study design	4	Present key elements of study design early in the paper	✓	5
Setting	5	Describe the setting, locations, and relevant dates, including periods of recruitment, exposure, follow-up, and data collection	✓	5
Participants	6	(*a*) Give the eligibility criteria, and the sources and methods of selection of participants	✓	5-6
Variables	7	Clearly define all outcomes, exposures, predictors, potential confounders, and effect modifiers. Give diagnostic criteria, if applicable	✓	6-7
Data sources/ measurement	8*	For each variable of interest, give sources of data and details of methods of assessment (measurement). Describe comparability of assessment methods if there is more than one group	✓	*Additional file 2*
Bias	9	Describe any efforts to address potential sources of bias (Adjusted for clustering effect)	✓	7
Study size	10	Explain how the study size was arrived at	✓	5
Quantitative variables	11	Explain how quantitative variables were handled in the analyses. If applicable, describe which groupings were chosen and why	✓	7-8
Statistical methods	12	(*a*) Describe all statistical methods, including those used to control for confounding	✓	8
		(*b*) Describe any methods used to examine subgroups and interactions	✓	8
		(*c*) Explain how missing data were addressed	No missing data	
		(*d*) If applicable, describe analytical methods taking account of sampling strategy	✓	7
		(*e*) Describe any sensitivity analyses	Not applicable	
**Results**				
Participants	13*	(a) Report numbers of individuals at each stage of study—eg numbers potentially eligible, examined for eligibility, confirmed eligible, included in the study, completing follow-up, and analysed	✓	8-9

For peer review only - http://bmjopen.bmj.com/site/about/guidelines.xhtml

		(b) Give reasons for non-participation at each stage	Not applicable	
		(c) Consider use of a flow diagram	Not applicable	
Descriptive data	14*	(a) Give characteristics of study participants (eg demographic, clinical, social) and information on exposures and potential confounders	✓	8-9
		(b) Indicate number of participants with missing data for each variable of interest	Not applicable	
Outcome data	15*	Report numbers of outcome events or summary measures	✓	10
Main results	16	(*a*) Give unadjusted estimates and, if applicable, confounder-adjusted estimates and their precision (eg, 95% confidence interval). Make clear which confounders were adjusted for and why they were included	✓	11
		(*b*) Report category boundaries when continuous variables were categorized	✓	9 & 11
		(*c*) If relevant, consider translating estimates of relative risk into absolute risk for a meaningful time period		Not applicable
Other analyses	17	Report other analyses done—eg analyses of subgroups and interactions, and sensitivity analyses	Not applicable	
**Discussion**				
Key results	18	Summarise key results with reference to study objectives	✓	16
Limitations	19	Discuss limitations of the study, taking into account sources of potential bias or imprecision. Discuss both direction and magnitude of any potential bias	✓	16-17
Interpretation	20	Give a cautious overall interpretation of results considering objectives, limitations, multiplicity of analyses, results from similar studies, and other relevant evidence	✓	17-21
Generalisability	21	Discuss the generalisability (external validity) of the study results	✓	17
**Other information**				
Funding	22	Give the source of funding and the role of the funders for the present study and, if applicable, for the original study on which the present article is based	✓	22

*Give information separately for exposed and unexposed groups.

For peer review only

Checklist for Reporting Of Survey Studies (CROSS)

Section/topic	Item	Item description	Reported on page #
**Title and abstract**			
Title and abstract	1a	State the word "survey" along with a commonly used term in title or abstract to introduce the study's design.	1, 2
	1b	Provide an informative summary in the abstract, covering background, objectives, methods, findings/results, interpretation/discussion, and conclusions.	2
**Introduction**			
Background	2	Provide a background about the rationale of study, what has been previously done, and why this survey is needed.	4, 5
Purpose/aim	3	Identify specific purposes, aims, goals, or objectives of the study.	5
**Methods**			
Study design	4	Specify the study design in the methods section with a commonly used term (e.g., cross-sectional or longitudinal).	6
Data collection methods	5a	Describe the questionnaire (e.g., number of sections, number of questions, number and names of instruments used).	7-8
	5b	Describe all questionnaire instruments that were used in the survey to measure particular concepts. Report target population, reported validity and reliability information, scoring/classification procedure, and reference links (if any).	7-8
	5c	Provide information on pretesting of the questionnaire, if performed (in the article or in an online supplement). Report the method of pretesting, number of times questionnaire was pre-tested, number and demographics of participants used for pretesting, and the level of similarity of demographics between pre-testing participants and sample population.	7
	5d	Questionnaire if possible, should be fully provided (in the article, or as appendices or as an online supplement).	7
Sample characteristics	6a	Describe the study population (i.e., background, locations, eligibility criteria for participant inclusion in survey, exclusion criteria).	7
	6b	Describe the sampling techniques used (e.g., single stage or multistage sampling, simple random sampling, stratified sampling, cluster sampling, convenience sampling). Specify the locations of sample participants whenever clustered sampling was applied.	6
	6c	Provide information on sample size, along with details of sample size calculation.	6
	6d	Describe how representative the sample is of the study population (or target population if possible), particularly for population-based surveys.	6
Survey	7a	Provide information on modes of questionnaire administration, including the type and number of contacts, the location where the survey was conducted (e.g., outpatient	7-8

For peer review only

administration		room or by use of online tools, such as SurveyMonkey).	
	7b	Provide information of survey's time frame, such as periods of recruitment, exposure, and follow-up days.	6
	7c	Provide information on the entry process:<br>–>For non-web-based surveys, provide approaches to minimize human error in data entry.<br>–>For web-based surveys, provide approaches to prevent "multiple participation" of participants.	8
Study preparation	8	Describe any preparation process before conducting the survey (e.g., interviewers' training process, advertising the survey).	7
Ethical considerations	9a	Provide information on ethical approval for the survey if obtained, including informed consent, institutional review board [IRB] approval, Helsinki declaration, and good clinical practice [GCP] declaration (as appropriate).	7
	9b	Provide information about survey anonymity and confidentiality and describe what mechanisms were used to protect unauthorized access.	7
Statistical analysis	10a	Describe statistical methods and analytical approach. Report the statistical software that was used for data analysis.	8
	10b	Report any modification of variables used in the analysis, along with reference (if available).	8
	10c	Report details about how missing data was handled. Include rate of missing items, missing data mechanism (i.e., missing completely at random [MCAR], missing at random [MAR] or missing not at random [MNAR]) and methods used to deal with missing data (e.g., multiple imputation).	*NA
	10d	State how non-response error was addressed.	*NA
	10e	For longitudinal surveys, state how loss to follow-up was addressed.	*NA
	10f	Indicate whether any methods such as weighting of items or propensity scores have been used to adjust for non-representativeness of the sample. **(Adjustment for clustering was done using the svy set command)**	9
	10g	Describe any sensitivity analysis conducted.	*NA
**Results**			
Respondent characteristics	11a	Report numbers of individuals at each stage of the study. Consider using a flow diagram, if possible.	*NA
	11b	Provide reasons for non-participation at each stage, if possible.	*NA
	11c	Report response rate, present the definition of response rate or the formula used to calculate response rate.	9

For peer review only

	11d	Provide information to define how unique visitors are determined. Report number of unique visitors along with relevant proportions (e.g., view proportion, participation proportion, completion proportion).	*NA
Descriptive results	12	Provide characteristics of study participants, as well as information on potential confounders and assessed outcomes.	10
	13a	Give unadjusted estimates and, if applicable, confounder-adjusted estimates along with 95% confidence intervals and p-values.	14 & 16
Main findings	13b	For multivariable analysis, provide information on the model building process, model fit statistics, and model assumptions (as appropriate).	14 & 16
	13c	Provide details about any sensitivity analysis performed. If there are considerable amount of missing data, report sensitivity analyses comparing the results of complete cases with that of the imputed dataset (if possible).	*NA
**Discussion**			
Limitations	14	Discuss the limitations of the study, considering sources of potential biases and imprecisions, such as non-representativeness of sample, study design, important uncontrolled confounders.	18
Interpretations	15	Give a cautious overall interpretation of results, based on potential biases and imprecisions and suggest areas for future research.	18-22
Generalizability	16	Discuss the external validity of the results.	18
**Other sections**			
Role of funding source	17	State whether any funding organization has had any roles in the survey's design, implementation, and analysis.	23
Conflict of interest	18	Declare any potential conflict of interest.	23
Acknowledgements	19	Provide names of organizations/persons that are acknowledged along with their contribution to the research.	24

***NA- Not applicable**
